# Supplementary material for: Stigmatization from Work-Related COVID-19 Exposure: A Systematic Review with Meta-Analysis
Source: Int J Environ Res Public Health. 2021 Jun 8;18(12):6183. doi: 10.3390/ijerph18126183 (PMC8226580; doi:10.3390/ijerph18126183)
Supplement: Supplementary file 1 [file ijerph-18-06183-s001.zip › Supplementary_ijerph-1235285/Table_S1.pdf]

EMBASE (via Ovid)

#	Search term
1	bully*.tw. or bullied.tw. or exp bullying/ or discriminat*.tw. or exp social discrimination/ or prejudice.tw. or exp prejudice/ or stigma*.tw. or stereotyp*.tw. or exp stereotyping/ or ignoran*.tw. or hostil*.tw. or exp hostility/ or intoleran*.tw. or marginali*.tw. or exp social exclusion/ or exp social attitude/ or exp social distance/ or exp social rejection/ or mob*.tw. or mobbing.tw.
2	exp respiratory tract infection/ or respiratory tract infection.tw. or (respiratory and (infect* or illness or symptom* or virus* or syndrome)).tw. or bronchit*.tw. or exp bronchitis/ or common cold.tw. or exp common cold/ or coryza.tw. or flu.tw. or laryngit*.tw. or exp laryngitis/ or pharyngit*.tw. or nasopharyngit*.tw. or rhinopharyngit*.tw. or exp pharyngitis/ or cough.tw. or exp coughing/ or tonsillit*.tw. or exp tonsillitis/ or pneumon*.tw. or bronchopneumon*.tw. or pleuropneumon*.tw. or exp pneumonia/ or sinusit*.tw. or rhinosinusit*.tw. or nasosinusit*.tw. or exp sinusitis/ or ((acute or viral or virus* or bacter*) and rhinit*).tw. or (throat* and (infect* or inflam*)).tw.
3	(coronavir* or corona virus* or betacoronavir* or covid19 or covid 19 or nCoV or CoV 2 or CoV2 or sarscov2 or 2019nCoV or 2019 novel coronavirus* or 2019 novel CoV or wuhan virus*).mp. or ((wuhan or hubei or huanan) and (severe acute respiratory or pneumonia*) and outbreak*).mp. or exp Coronavirus infection/ or exp Coronavirus/ or exp Betacoronavirus/
4	exp severe acute respiratory syndrome/ or exp SARS coronavirus/ or SARS.tw. or severe acute respiratory syndrome.tw.
5	exp Middle East respiratory syndrome coronavirus/ or MERS.tw. or middle east respiratory syndrome.tw.
6	exp influenza/ or exp "influenza A (H1N1)"/ or exp "Influenza A virus (H1N1)"/ or exp "Influenza A virus (H7N9)"/ or influenza.tw. or H1N1.tw. or H7N9.tw.
7	or/2-6
8	(profession* or job or occupation* or work* or employ*).tw.
9	1 and 7 and 8

PsycInfo (via EBSCOhost)

#	Search term
1	(TX bully*) OR (TX bullied) OR (DE “Bullying”) OR (TX discriminat*) OR (DE “Social discrimination”) OR (TX prejudice) OR (DE “Prejudice”) OR (TX stigma*) OR (TX stereotyp*) OR (DE “Stereotyped Attitudes”) OR (TX ignoran*) OR (TX hostil*) OR (DE “Hostility”) OR (TX intoleran*) OR (TX marginali*) OR (DE “Marginalization”) OR (DE “Social Perception”) OR (DE “Social Acceptance”) OR (TX mob*) OR (DE “mobbing”)
2	(DE “Respiratory Tract Disorders”) OR (TX “respiratory tract infection”) OR (TX (respiratory AND (infect* OR illness OR symptom* OR virus* OR syndrome)) OR (TX bronchit*) OR (TX “common cold”) OR (TX coryza) OR (TX flu) OR (TX laryngit*) OR (DE “Laryngeal Disorders”) OR (TX pharyngit*) OR (TX nasopharyngit*) OR (TX rhinopharyngit*) OR (DE “Pharyngeal Disorders”) OR (TX cough) OR (TX tonsillit*) OR (TX pneumon*) OR (TX bronchopneumon*) OR (TX pleuropneumon*) OR (DE “Pneumonia”) OR (TX sinusit*) OR (TX rhinosinusit*) OR (TX nasosinusit*) OR (TX (acute OR viral OR virus* OR bacter*) AND rhinit*) OR (TX (throat* AND (infect* OR inflam*))
3	(TX coronavir*) OR (TX corona virus*) OR (TX betacoronavir*) OR (TX “covid 19”) OR (TX nCoV) OR (TX “CoV 2”) OR (TX CoV2) OR (TX sarscov2) OR (TX 2019nCoV) OR (TX “2019 novel coronavirus*”) OR (TX “2019 novel CoV”) OR (TX “wuhan virus*”) OR (TX (wuhan

	OR hubei OR huanan) AND (severe acute respiratory OR pneumonia*) AND (outbreak*)) OR (DE “Coronavirus”)
4	(DE “Severe Acute Respiratory Syndrome”) OR (TX SARS) OR (TX “Severe Acute Respiratory Syndrome”)
5	(DE “Middle East Respiratory Syndrome”) OR (TX MERS) OR (TX “Middle East Respiratory Syndrome”)
6	(DE “Swine Influenza”) OR (DE “Influenza”) OR (TX influenza) OR (TX H1N1) OR (TX H7N9)
7	S2 OR S3 OR S4 OR S5 OR S6
8	(TX profession*) OR (TX job) OR (TX occupation*) OR (TX work*) OR (TX employ*)
9	S1 AND S7 AND S8

MEDLINE (via PubMed)

#	**Search term**
1	bully*[tw] OR bullied[tw] OR bullying[mh] OR discriminat*[tw] OR “social discrimination”[mh] OR prejudice[tw] OR prejudice[mh] OR stigma*[tw] OR stereotyp*[tw] OR stereotyping[mh] OR ignoran*[tw] OR hostil*[tw] OR hostility[mh] OR intoleran*[tw] OR marginali*[tw] OR “social marginalization”[mh] OR “social perception”[mh] OR attitude[mh] OR “social distance”[mh] OR "rejection, psychology"[mh] OR mob*[tw] OR mobbing[tw]
2	"respiratory tract infections"[mh] OR "respiratory tract infections"[tw] OR (respiratory[tw] AND (infect*[tw] OR illness[tw] OR symptom*[tw] OR virus*[tw] OR syndrome[tw])) OR bronchitis[mh] OR bronchit*[tw] OR “common cold”[mh] OR “common cold”[tw] OR coryza[tw] OR flu[tw] OR laryngitis[mh] OR laryngit*[tw] OR pharyngitis[mh] OR pharyngit*[tw] OR nasopharyngit*[tw] OR rhinopharyngit*[tw] OR cough[mh] OR cough[tw] OR tonsillitis[mh] OR tonsillit*[tw] OR pneumonia[mh] OR pneumon*[tw] OR bronchopneumon*[tw] OR pleuropneumon*[tw] OR sinusitis[mh] OR sinusit*[tw] OR rhinosinusit*[tw] OR nasosinusit*[tw] OR ((acute[tw] OR viral[tw] OR virus*[tw] OR bacter*[tw]) AND (rhinit*[tw])) OR ((throat*[tw]) AND (infect*[tw] OR inflam*[tw]))
3	(coronavirus OR “corona virus” OR coronavirinae OR coronaviridae OR betacoronavirus OR covid19 OR “covid 19” OR nCoV OR “CoV 2” OR CoV2 OR sarscov2 OR 2019nCoV OR “novel CoV” OR “wuhan virus”) OR ((wuhan OR hubei OR huanan) AND (“severe acute respiratory” OR pneumonia) AND (outbreak)) OR “Coronavirus”[Mesh] OR “Coronavirus Infections”[Mesh] OR “COVID-19” [Supplementary Concept] OR “severe acute respiratory syndrome coronavirus 2” [Supplementary Concept] OR “Betacoronavirus”[Mesh]
4	“sars virus”[mh] OR "severe acute respiratory syndrome"[mh] OR SARS[tw] OR "severe acute respiratory syndrome"[tw]
5	MERS[tw] OR "middle east respiratory syndrome coronavirus"[mh] OR "middle east respiratory syndrome”[tw]
6	“influenza, human"[mh] OR influenza[tw] OR "influenza a virus, h1n1 subtype"[mh] OR H1N1[tw] OR "Influenza A Virus, H7N9 Subtype"[mh] OR H7N9[tw]
7	#2 OR #3 OR #4 OR #5 OR #6
8	profession*[tw] OR job[tw] OR occupation*[tw] OR work*[tw] OR employ*[tw]
9	**#1 AND #7 AND #8**

Preprint Server - ZB MED

#	Search term
	((((((((((((bully*) OR bullied*) OR discriminat*) OR predjudice) OR stigma*) OR stereotyp*) OR ignoran*) OR hostil*) OR intoleran*) OR marginali*) OR attitude) OR social distance) OR rejection) OR mobbing

CINAHL

#	Search term
1	(TX bully*) OR (TX bullied) OR (DE "Bullying") OR (TX discriminat*) OR (DE "Social discrimination") OR (TX prejudice) OR (DE "Prejudice") OR (TX stigma*) OR (TX stereotyp*) OR (DE "Stereotyped Attitudes") OR (TX ignoran*) OR (TX hostil*) OR (DE "Hostility") OR (TX intoleran*) OR (TX marginali*) OR (DE "Marginalization") OR (DE "Social Perception") OR (DE "Social Acceptance")
2	(DE "Respiratory Tract Disorders") OR (TX "respiratory tract infection") OR (TX (respiratory) AND (infect* OR illness OR symptom* OR virus* OR syndrome)) OR (TX bronchit*) OR (TX "common cold") OR (TX coryza) OR (TX flu) OR (TX laryngit*) OR (DE "Laryngeal Disorders") OR (TX pharyngit*) OR (TX nasopharyngit*) OR (TX rhinopharyngit*) OR (DE "Pharyngeal Disorders") OR (TX cough) OR (TX tonsillit*) OR (TX pneumon*) OR (TX bronchopneumon*) OR (TX pleuropneumon*) OR (DE "Pneumonia") OR (TX sinusit*) OR (TX rhinosinusit*) OR (TX nasosinusit*) OR (TX (acute OR viral OR virus* OR bacter*) AND rhinit*) OR (TX (throat* AND (infect* OR inflam*))
3	(TX coronavir*) OR (TX corona virus*) OR (TX betacoronavir*) OR (TX "covid 19") OR (TX nCoV) OR (TX "CoV 2") OR (TX CoV2) OR (TX sarscov2) OR (TX 2019nCoV) OR (TX "2019 novel coronavirus*") OR (TX "2019 novel CoV") OR (TX "wuhan virus*") OR (TX (wuhan OR hubei OR huanan) AND (severe acute respiratory OR pneumonia*) AND (outbreak*)) OR (DE "Coronavirus")
4	(DE "Severe Acute Respiratory Syndrome") OR (TX SARS) OR (TX "Severe Acute Respiratory Syndrome")
5	(DE "Middle East Respiratory Syndrome") OR (TX MERS) OR (TX "Middle East Respiratory Syndrome")
6	(DE "Swine Influenza") OR (DE "Influenza") OR (TX influenza) OR (TX H1N1) OR (TX H7N9)
7	S2 OR S3 OR S4 OR S5 OR S6
8	(DE "health personnel") OR (TX "health personnel") OR (TX healthcare AND TX workers) OR (TX "healthcare workers") OR (DE "nurses") OR (TX nurses) OR (TX nurse) OR (DE "physicians") OR (TX physicians) OR (DE "Medical Staff, Hospital") OR (TX "healthcare professionals") OR (TX "healthcare staff") OR (TX "medical staff") OR (TX "care workers") OR (TX "care personnel") OR (TX "nursing staff") OR (TX "nursing personnel") OR (TX caregivers) OR (TX carer)
9	S1 AND S7 AND S8
