# Supplementary material for: Stigmatization from Work-Related COVID-19 Exposure: A Systematic Review with Meta-Analysis
Source: Int J Environ Res Public Health. 2021 Jun 8;18(12):6183. doi: 10.3390/ijerph18126183 (PMC8226580; doi:10.3390/ijerph18126183)
Supplement: Supplementary file 1 [file ijerph-18-06183-s001.zip › Supplementary_ijerph-1235285/Table_S2.pdf]

Excluded references (n = 154)

Editorials, comments, letters, narrative reviews (n = 68)

1. Aacharya RP, Shah A. Ethical dimensions of stigma and discrimination in Nepal during COVID-19 pandemic. Ethics Med Public Health. 2020 Jul-Sep;14:100536. doi: 10.1016/j.jemep.2020.100536. Epub 2020 May 27. PMID: 32835054; PMCID: PMC7250747.
2. Adiamah A, Moody N, Blackburn L, Dickson E, Thompson A, Reilly JJ, Saunders J, Brooks A; On behalf of the The ICON Trauma Study Group. ICON Trauma (Impact of COVID-19 on Major Trauma workload) Study. Br J Surg. 2020 Sep;107(10):e412-e413. doi: 10.1002/bjs.11855. Epub 2020 Aug 4. PMID: 32749673; PMCID: PMC7436320.
3. Agyemang-Duah W, Morgan AK, Oduro Appiah J, Peprah P, Fordjour AA. Re-integrating Older Adults Who Have Recovered from the Novel Coronavirus into Society in the Context of Stigmatization: Lessons for Health and Social Actors in Ghana. J Gerontol Soc Work. 2020 Aug-Oct;63(6-7):691-693. doi: 10.1080/01634372.2020.1779163. Epub 2020 Jul 16. PMID: 32672136.
4. Ali SH. Stigmatized ethnicity, public health, and globalization. Can Ethn Stud. 2008;40(3):43-64. doi: 10.1353/ces.2008.0002. PMID: 21847845.
5. Anmella G, Fico G, Roca A, Gómez-Ramiro M, Vázquez M, Murru A, Pacchiarotti I, Verdolini N, Vieta E. Unravelling potential severe psychiatric repercussions on healthcare professionals during the COVID-19 crisis. J Affect Disord. 2020 Aug 1;273:422-424. doi: 10.1016/j.jad.2020.05.061. Epub 2020 May 16. PMID: 32425275; PMCID: PMC7228876.
6. Bell CA, Crabtree SA, Hall EL, Sandage SJ. Research in counselling and psychotherapy Post-COVID-19. Couns Psychother Res. 2020 Jul 3:10.1002/capr.12334. doi: 10.1002/capr.12334. Epub ahead of print. PMID: 32837330; PMCID: PMC7362162.
7. Bhattacharya, K., & Bhattacharya, N. (2020). Health care workers facing Social Ostracism during COVID-19. JIMA, 2020; 118(08), 22-24.
8. Brito-Brito PR, Fernández-Gutiérrez DÁ, Cuéllar-Pompa L. Manejo emocional de la crisis sanitaria ante el coronavirus: una revisión narrativa [Emotional management of the health crisis by coronavirus: A narrative review]. Enferm Clin. 2021 Feb;31:S107-S111. Spanish. doi: 10.1016/j.enfcli.2020.05.010. Epub 2020 May 18. PMID: 32425490; PMCID: PMC7231732.
9. Brown MM. Don't be the "Fifth Guy": Risk, Responsibility, and the Rhetoric of Handwashing Campaigns. J Med Humanit. 2019 Jun;40(2):211-224. doi: 10.1007/s10912-017-9470-4. PMID: 28853016; PMCID: PMC7088277.
10. Bruns DP, Kraguljac NV, Bruns TR. COVID-19: Facts, Cultural Considerations, and Risk of Stigmatization. J Transcult Nurs. 2020 Jul;31(4):326-332. doi: 10.1177/1043659620917724. Epub 2020 Apr 21. PMID: 32316872; PMCID: PMC7324134.
11. Chakraborty, N. (2020). The COVID-19 pandemic and its impact on mental health. Prog Neurol Psychiatry 2020; *24*(2), 21-24. doi: 10.1002/pnp.666.
12. Chander R, Murugesan M, Ritish D, Damodharan D, Arunachalam V, Parthasarathy R, Raj A, Sharma MK, Manjunatha N, Bada Math S, Kumar CN. Addressing the mental health concerns of migrant workers during the COVID-19 pandemic: An experiential account. Int J Soc Psychiatry. 2020 Jun 29:20764020937736. doi: 10.1177/0020764020937736. Epub ahead of print. PMID: 32594816.
13. Chandratre S. Medical Students and COVID-19: Challenges and Supportive Strategies. J Med Educ Curric Dev. 2020 Jun 24;7:2382120520935059. doi: 10.1177/2382120520935059. PMID: 32637642; PMCID: PMC7315659.
14. Chersich MF, Gray G, Fairlie L, Eichbaum Q, Mayhew S, Allwood B, English R, Scorgie F, Luchters S, Simpson G, Haghighi MM, Pham MD, Rees H. COVID-19 in Africa: care and protection for frontline healthcare workers. Global Health. 2020 May 15;16(1):46. doi: 10.1186/s12992-020-00574-3. PMID: 32414379; PMCID: PMC7227172.
15. Cho HJ, Feldman LS, Keller S, Hoffman A, Pahwa AK, Krouss M. Choosing Wisely in the COVID-19 Era: Preventing Harm to Healthcare Workers. J Hosp Med. 2020 Jun;15(6):360-362. doi: 10.12788/jhm.3457. PMID: 32490804; PMCID: PMC7289510.
16. Chopra KK, Arora VK. Covid-19 and social stigma: Role of scientific community. Indian J Tuberc. 2020 Jul;67(3):284-285. doi: 10.1016/j.ijtb.2020.07.012. Epub 2020 Jul 15. PMID: 32825854; PMCID: PMC7362777.
17. Dhai A, Veller M, Ballot D, Mokhachane M. Pandemics, professionalism and the duty of care: Concerns from the coalface. S Afr Med J. 2020 May 14;110(6):450-452. PMID: 32880548.

18. Dubey S, Biswas P, Ghosh R, Chatterjee S, Dubey MJ, Chatterjee S, Lahiri D, Lavie CJ. Psychosocial impact of COVID-19. Diabetes Metab Syndr. 2020 Sep-Oct;14(5):779-788. doi: 10.1016/j.dsx.2020.05.035. Epub 2020 May 27. PMID: 32526627; PMCID: PMC7255207.
19. Dudley MJ. Reaching Invisible and Unprotected Workers on Farms during the Coronavirus Pandemic. J Agromedicine. 2020 Oct;25(4):427-429. doi: 10.1080/1059924X.2020.1815625. Epub 2020 Sep 7. PMID: 32894685.
20. Elbeddini A, Wen CX, Tayefehchamani Y, To A. Mental health issues impacting pharmacists during COVID-19. J Pharm Policy Pract. 2020 Jul 22;13:46. doi: 10.1186/s40545-020-00252-0. PMID: 32704376; PMCID: PMC7374072.
21. El-Hage W, Hingray C, Lemogne C, Yrondi A, Brunault P, Bienvenu T, Etain B, Paquet C, Gohier B, Bennabi D, Birmes P, Sauvaget A, Fakra E, Prieto N, Bulteau S, Vidailhet P, Camus V, Leboyer M, Krebs MO, Aouizerate B. Les professionnels de santé face à la pandémie de la maladie à coronavirus (COVID-19) : quels risques pour leur santé mentale ? [Health professionals facing the coronavirus disease 2019 (COVID-19) pandemic: What are the mental health risks?]. Encephale. 2020 Jun;46(3S):S73-S80. French. doi: 10.1016/j.encep.2020.04.008. Epub 2020 Apr 22. PMID: 32370984; PMCID: PMC7174182.
22. Galbraith N, Boyda D, McFeeters D, Hassan T. The mental health of doctors during the COVID-19 pandemic. BJPsych Bull. 2021 Apr;45(2):93-97. doi: 10.1192/bjb.2020.44. PMID: 32340645; PMCID: PMC7322151.
23. Glover RE, van Schalkwyk MCI, Akl EA, Kristjannson E, Lotfi T, Petkovic J, Petticrew MP, Pottie K, Tugwell P, Welch V. A framework for identifying and mitigating the equity harms of COVID-19 policy interventions. J Clin Epidemiol. 2020 Dec;128:35-48. doi: 10.1016/j.jclinepi.2020.06.004. Epub 2020 Jun 9. PMID: 32526461; PMCID: PMC7280094.
24. Grover S, Singh P, Sahoo S, Mehra A. Stigma related to COVID-19 infection: Are the Health Care Workers stigmatizing their own colleagues? Asian J Psychiatr. 2020 Oct;53:102381. doi: 10.1016/j.ajp.2020.102381. Epub 2020 Aug 27. PMID: 32882670; PMCID: PMC7451190.
25. Gunnell D, Appleby L, Arensman E, Hawton K, John A, Kapur N, Khan M, O'Connor RC, Pirkis J; COVID-19 Suicide Prevention Research Collaboration. Suicide risk and prevention during the COVID-19 pandemic. Lancet Psychiatry. 2020 Jun;7(6):468-471. doi: 10.1016/S2215-0366(20)30171-1. Epub 2020 Apr 21. PMID: 32330430; PMCID: PMC7173821.
26. Janeway D. The Role of Psychiatry in Treating Burnout Among Nurses During the Covid-19 Pandemic. J Radiol Nurs. 2020 Sep;39(3):176-178. doi: 10.1016/j.jradnu.2020.06.004. Epub 2020 Jul 23. PMID: 32837392; PMCID: PMC7377731.
27. Leszcz M, Maunder R, Hunter J. Psychological support for health care workers during the COVID-19 pandemic. CMAJ. 2020 Jun 15;192(24):E660. doi: 10.1503/cmaj.75864. PMID: 32540910; PMCID: PMC7828846.
28. Lynch D, O'Brien M, O'Byrne L, Gavin B, McNicholas F. Impact and Mechanism of Covid-19 on Mental Health and Wellbeing. Ir Med J. 2020 Jun 11;113(6):100. PMID: 32816435.
29. Kallakuri S, Maulik PK. Challenges facing individuals and researchers: suicide in India in the COVID-19 pandemic. Lancet Psychiatry. 2020 Aug;7(8):e49. doi: 10.1016/S2215-0366(20)30254-6. PMID: 32711712; PMCID: PMC7377764.
30. Kang L, Li Y, Hu S, Chen M, Yang C, Yang BX, Wang Y, Hu J, Lai J, Ma X, Chen J, Guan L, Wang G, Ma H, Liu Z. The mental health of medical workers in Wuhan, China dealing with the 2019 novel coronavirus. Lancet Psychiatry. 2020 Mar;7(3):e14. doi: 10.1016/S2215-0366(20)30047-X. Epub 2020 Feb 5. PMID: 32035030; PMCID: PMC7129673.
31. Kantamneni N. The impact of the COVID-19 pandemic on marginalized populations in the United States: A research agenda. J Vocat Behav. 2020 Jun;119:103439. doi: 10.1016/j.jvb.2020.103439. Epub 2020 May 8. PMID: 32390658; PMCID: PMC7205696.
32. Karim MR, Islam MT, Talukder B. COVID-19's impacts on migrant workers from Bangladesh: In search of policy intervention. World Dev. 2020 Dec;136:105123. doi: 10.1016/j.worlddev.2020.105123. Epub 2020 Aug 1. PMID: 32834394; PMCID: PMC7395589.
33. Kaufman KR, Petkova E, Bhui KS, Schulze TG. A global needs assessment in times of a global crisis: world psychiatry response to the COVID-19 pandemic. BJPsych Open. 2020 Apr 6;6(3):e48. doi: 10.1192/bjo.2020.25. PMID: 32250235; PMCID: PMC7211994.

34. Kontoangelos K, Economou M, Papageorgiou C. Mental Health Effects of COVID-19 Pandemia: A Review of Clinical and Psychological Traits. Psychiatry Investig. 2020 Jun;17(6):491-505. doi: 10.30773/pi.2020.0161. Epub 2020 Jun 15. PMID: 32570296; PMCID: PMC7324731.
35. Kumar S, Rathore P, Shweta, Krishnapriya V, Thankachan A, Haokip N, Kumar B, Bhatnagar S. Challenges Encountered While Providing Holistic Care to a Cluster of COVID-19 Patients. Indian J Palliat Care. 2020 Jun;26(Suppl 1):S53-S55. doi: 10.4103/IJPC.IJPC_147_20. Epub 2020 Jun 30. PMID: 33088088; PMCID: PMC7535016.
36. Krystal JH. Responding to the hidden pandemic for healthcare workers: stress. Nat Med. 2020 May;26(5):639. doi: 10.1038/s41591-020-0878-4. Erratum in: Nat Med. 2020 Dec;26(12):1951. PMID: 32350461.
37. Lotta G, Wenham C, Nunes J, Pimenta DN. Community health workers reveal COVID-19 disaster in Brazil. Lancet. 2020 Aug 8;396(10248):365-366. doi: 10.1016/S0140-6736(20)31521-X. Epub 2020 Jul 10. PMID: 32659212; PMCID: PMC7351420.
38. Mahmoudi M, Keashly L. COVID-19 pandemic may fuel academic bullying. Bioimpacts. 2020;10(3):139-140. doi: 10.34172/bi.2020.17. Epub 2020 Jun 27. PMID: 32793435; PMCID: PMC7416009.
39. Major A, Hlubocky FJ. Mental health of health care workers during the COVID-19 pandemic and evidence-based frameworks for mitigation: A rapid review. 2021. medRxiv. doi: 10.1101/2021.01.03.21249166.
40. Morioka S, Saito S, Hayakawa K, Takasaki J, Suzuki T, Ide S, Nakamura K, Moriyama Y, Akiyama Y, Miyazato Y, Nomoto H, Nakamoto T, Ota M, Sakamoto K, Katsuno T, Kusaba Y, Ishikane M, Kinoshita N, Yamamoto K, Ujiie M, Kutsuna S, Hashimoto M, Suzuki M, Izumi S, Katagiri D, Hojo M, Ohmagari N. Psychiatric burdens or stress during hospitalization and concerns after discharge in patients with severe acute respiratory syndrome coronavirus-2 isolated in a tertiary care hospital. Psychiatry Res. 2020 Apr 29;289:113040. doi: 10.1016/j.psychres.2020.113040. Epub ahead of print. PMID: 33242806.
41. Mukherjee S. Disparities, desperation, and divisiveness: Coping with COVID-19 in India. Psychol Trauma. 2020 Sep;12(6):582-584. doi: 10.1037/tra0000682. Epub 2020 Aug 10. PMID: 32772533.
42. Mukhtar S. Psychological health during the coronavirus disease 2019 pandemic outbreak. Int J Soc Psychiatry. 2020 Aug;66(5):512-516. doi: 10.1177/0020764020925835. Epub 2020 May 21. PMID: 32434402; PMCID: PMC7405632.
43. Nuckchady DC. Stigma Linked to COVID-19. Int Neuropsychiatr Dis J. 2021; *15*(1): 29-38. https://doi.org/10.9734/indj/2021/v15i130144
44. Ogunleye OO, Basu D, Mueller D, Sneddon J, Seaton RA, Yinka-Ogunleye AF, Wamboga J, Miljković N, Mwita JC, Rwegerera GM, Massele A, Patrick O, Niba LL, Nsaikila M, Rashed WM, Hussein MA, Hegazy R, Amu AA, Boahen-Boaten BB, Matsebula Z, Gwebu P, Chirigo B, Mkhabela N, Dlamini T, Sithole S, Malaza S, Dlamini S, Afriyie D, Asare GA, Amponsah SK, Sefah I, Oluka M, Guantai AN, Opanga SA, Sarele TV, Mafisa RK, Chikowe I, Khuluza F, Kibuule D, Kalemeera F, Mubita M, Fadare J, Sibomana L, Ramokgopa GM, Whyte C, Maimela T, Hugo J, Meyer JC, Schellack N, Rampamba EM, Visser A, Alfadl A, Malik EM, Malande OO, Kalungia AC, Mwila C, Zaranyika T, Chaibva BV, Olaru ID, Masuka N, Wale J, Hwenda L, Kamoga R, Hill R, Barbui C, Bochenek T, Kurdi A, Campbell S, Martin AP, Phuong TNT, Thanh BN, Godman B. Response to the Novel Corona Virus (COVID-19) Pandemic Across Africa: Successes, Challenges, and Implications for the Future. Front Pharmacol. 2020 Sep 11;11:1205. doi: 10.3389/fphar.2020.01205. PMID: 33071775; PMCID: PMC7533592.
45. Oladeru OT, Tran NT, Al-Rousan T, Williams B, Zaller N. A call to protect patients, correctional staff and healthcare professionals in jails and prisons during the COVID-19 pandemic. Health Justice. 2020 Jul 2;8(1):17. doi: 10.1186/s40352-020-00119-1. PMID: 32617825; PMCID: PMC7331488.
46. Otu A, Charles CH, Yaya S. Mental health and psychosocial well-being during the COVID-19 pandemic: the invisible elephant in the room. Int J Ment Health Syst. 2020 May 28;14:38. doi: 10.1186/s13033-020-00371-w. PMID: 32514302; PMCID: PMC7257210.
47. Patel MP, Kute VB, Agarwal SK; COVID-19 Working Group of Indian Society of Nephrology. "Infodemic" COVID 19: More Pandemic than the Virus. Indian J Nephrol. 2020 May-Jun;30(3):188-191. doi: 10.4103/ijn.IJN_216_20. Epub 2020 Jun 10. PMID: 33013069; PMCID: PMC7470201.
48. Petzold MB, Plag J, Ströhle A. Umgang mit psychischer Belastung bei Gesundheitsfachkräften im Rahmen der Covid-19-Pandemie [Dealing with psychological distress by healthcare professionals

during the COVID-19 pandemia]. Nervenarzt. 2020 May;91(5):417-421. German. doi: 10.1007/s00115-020-00905-0. PMID: 32221635; PMCID: PMC7100457.
49. Phougeishangbam, P. D., & Mayanglambam, P. Impact of COVID 19 on Health Care Personnel. Indian J Public Health Res Dev. 2020; 11(9), 79-83.
50. Ransing R, Ramalho R, de Filippis R, Ojeahere MI, Karaliuniene R, Orsolini L, Pinto da Costa M, Ullah I, Grandinetti P, Gashi Bytyçi D, Grigo O, Mhamunkar A, El Hayek S, Essam L, Larnaout A, Shalbafan M, Nofal M, Soler-Vidal J, Pereira-Sanchez V, Adiukwu F. Infectious disease outbreak related stigma and discrimination during the COVID-19 pandemic: Drivers, facilitators, manifestations, and outcomes across the world. Brain Behav Immun. 2020 Oct;89:555-558. doi: 10.1016/j.bbi.2020.07.033. Epub 2020 Jul 27. PMID: 32731007; PMCID: PMC7384410.
51. Reza-Paul S, Lazarus L, Haldar P, Reza Paul M, Lakshmi B, Ramaiah M, Pasha A, Hafeez Ur Rahman S, Venukumar KT, Venugopa MS, Rewari BB, Lorway R. Community action for people with HIV and sex workers during the COVID-19 pandemic in India. WHO South East Asia J Public Health. 2020 Sep;9(2):104-106. doi: 10.4103/2224-3151.294302. PMID: 32978341.
52. Reyes AT. A Mindfulness Mobile App for Traumatized COVID-19 Healthcare Workers and Recovered Patients: A Response to "The Use of Digital Applications and COVID-19". Community Ment Health J. 2020 Oct;56(7):1204-1205. doi: 10.1007/s10597-020-00690-9. Epub 2020 Aug 9. PMID: 32772205; PMCID: PMC7415191.
53. Rosen B, Preisman M, Hunter J, Maunder R. Applying Psychotherapeutic Principles to Bolster Resilience Among Health Care Workers During the COVID-19 Pandemic. Am J Psychother. 2020 Dec 1;73(4):144-148. doi: 10.1176/appi.psychotherapy.20200020. Epub 2020 Sep 28. PMID: 32985915.
54. Rzymski P, Nowicki M. Preventing COVID-19 prejudice in academia. Science. 2020 Mar 20;367(6484):1313. doi: 10.1126/science.abb4870. PMID: 32193314.
55. Semo BW, Frissa SM. The Mental Health Impact of the COVID-19 Pandemic: Implications for Sub-Saharan Africa. Psychol Res Behav Manag. 2020 Sep 3;13:713-720. doi: 10.2147/PRBM.S264286. PMID: 32982500; PMCID: PMC7508558.
56. Shabani M, Goffin T, Mertes H. Reporting, recording, and communication of COVID-19 cases in workplace: data protection as a moving target. J Law Biosci. 2020 Apr 22;7(1):lsaa008. doi: 10.1093/jlb/lsaa008. PMID: 32728456; PMCID: PMC7188145.
57. Sharma V, Scott J, Kelly J, VanRooyen MJ. Prioritizing vulnerable populations and women on the frontlines: COVID-19 in humanitarian contexts. Int J Equity Health. 2020 May 13;19(1):66. doi: 10.1186/s12939-020-01186-4. PMID: 32404178; PMCID: PMC7218503.
58. Sheather J, Hartwell A, Norcliffe-Brown D. Serious violations of health workers' rights during pandemic. BMJ. 2020 Jul 14;370:m2824. doi: 10.1136/bmj.m2824. PMID: 32665310.
59. Shigemura J, Kurosawa M. Mental health impact of the COVID-19 pandemic in Japan. Psychol Trauma. 2020 Jul;12(5):478-479. doi: 10.1037/tra0000803. Epub 2020 Jun 11. PMID: 32525392.
60. Sinanović O, Muftić M, Sinanović S. COVID-19 Pandemia: Neuropsychiatric Comorbidity and Consequences. Psychiatr Danub. 2020 Summer;32(2):236-244. doi: 10.24869/psyd.2020.236. PMID: 32796792.
61. Souadka A, Essangri H, Benkabbou A, Amrani L, Majbar MA. COVID-19 and Healthcare worker's families: behind the scenes of frontline response. EClinicalMedicine. 2020 May 3;23:100373. doi: 10.1016/j.eclinm.2020.100373. PMID: 32368726; PMCID: PMC7196421.
62. Tanoue Y, Nomura S, Yoneoka D, Kawashima T, Eguchi A, Shi S, Harada N, Miyata H. Mental health of family, friends, and co-workers of COVID-19 patients in Japan. Psychiatry Res. 2020 Sep;291:113067. doi: 10.1016/j.psychres.2020.113067. Epub 2020 May 6. PMID: 32535504; PMCID: PMC7202826.
63. Troisi, A. (2020). Fear of COVID-19: Insights from evolutionary behavioral science. Clin. Neuropsychiatry. 2020; 2(17):72-75. doi:10.36131/CN20200207.
64. Vega, M. Y. (2016). Combating stigma and fear: Applying psychosocial lessons learned from the HIV epidemic and SARS to the current Ebola crisis. In J. Kuriansky (Ed.), The psychosocial aspects of a deadly epidemic: What Ebola has taught us about holistic healing (pp. 271–286). Praeger
65. Voo TC, Clapham H, Tam CC. Ethical Implementation of Immunity Passports During the COVID-19 Pandemic. J Infect Dis. 2020 Aug 4;222(5):715-718. doi: 10.1093/infdis/jiaa352. PMID: 32582943; PMCID: PMC7337820.

66. Wang J, Lee YF, Zhou M. What is the best timing for health care workers infected with COVID-19 to return to work? Am J Infect Control. 2020 Sep;48(9):1128-1129. doi: 10.1016/j.ajic.2020.06.217. Epub 2020 Jul 3. PMID: 32628983; PMCID: PMC7836938.
67. Widjaja FF, Shatri H, Putranto R. Health Issues Among Healthcare Workers During COVID-19 Pandemic: a Psychosomatic Approach. Acta Med Indones. 2020 Apr;52(2):172-176. PMID: 32778632.
68. Zheng W. Mental health and a novel coronavirus (2019-nCoV) in China. J Affect Disord. 2020 May 15;269:201-202. doi: 10.1016/j.jad.2020.03.041. Epub 2020 Mar 21. PMID: 32339137; PMCID: PMC7174828.

Other new respiratory virus diseases (n=34)

1. Aiello A, Khayeri MY, Raja S, Peladeau N, Romano D, Leszcz M, Maunder RG, Rose M, Adam MA, Pain C, Moore A, Savage D, Schulman RB. Resilience training for hospital workers in anticipation of an influenza pandemic. J Contin Educ Health Prof. 2011 Winter;31(1):15-20. doi: 10.1002/chp.20096. PMID: 21425355.
2. Almutairi AF, Adlan AA, Balkhy HH, Abbas OA, Clark AM. "It feels like I'm the dirtiest person in the world.": Exploring the experiences of healthcare providers who survived MERS-CoV in Saudi Arabia. J Infect Public Health. 2018 Mar-Apr;11(2):187-191. doi: 10.1016/j.jiph.2017.06.011. Epub 2017 Jul 1. PMID: 28676285; PMCID: PMC7102804.
3. Bai Y, Lin CC, Lin CY, Chen JY, Chue CM, Chou P. Survey of stress reactions among health care workers involved with the SARS outbreak. Psychiatr Serv. 2004 Sep;55(9):1055-7. doi: 10.1176/appi.ps.55.9.1055. PMID: 15345768.
4. Barello S, Falcó-Pegueroles A, Rosa D, Tolotti A, Graffigna G, Bonetti L. The psychosocial impact of flu influenza pandemics on healthcare workers and lessons learnt for the COVID-19 emergency: a rapid review. Int J Public Health. 2020 Sep;65(7):1205-1216. doi: 10.1007/s00038-020-01463-7. Epub 2020 Sep 4. PMID: 32888048; PMCID: PMC7472941.
5. Bhaumik S, Moola S, Tyagi J, Nambiar D, Kakoti M. Community health workers for pandemic response: a rapid evidence synthesis. BMJ Glob Health. 2020 Jun;5(6):e002769. doi: 10.1136/bmjgh-2020-002769. PMID: 32522738; PMCID: PMC7292038.
6. Brooks SK, Dunn R, Amlôt R, Rubin GJ, Greenberg N. A Systematic, Thematic Review of Social and Occupational Factors Associated With Psychological Outcomes in Healthcare Employees During an Infectious Disease Outbreak. J Occup Environ Med. 2018 Mar;60(3):248-257. doi: 10.1097/JOM.0000000000001235. PMID: 29252922.
7. Cabello I R, Echavez JFM, Serrano-Ripoll, MJ, Fraile-Navarro Dd, Roque MAF, Moreno GP, et al. Impact of viral epidemic outbreaks on mental health of healthcare workers: a rapid systematic review. 2020; Medrxiv. doi:10.1101/2020.04.02.20048892
8. Chew QH, Wei KC, Vasoo S, Sim K. Psychological and Coping Responses of Health Care Workers Toward Emerging Infectious Disease Outbreaks: A Rapid Review and Practical Implications for the COVID-19 Pandemic. J Clin Psychiatry. 2020 Oct 20;81(6):20r13450. doi: 10.4088/JCP.20r13450. PMID: 33084255.
9. Choi JS, Kim JS. Factors influencing emergency nurses' ethical problems during the outbreak of MERS-CoV. Nurs Ethics. 2018 May;25(3):335-345. doi: 10.1177/0969733016648205. Epub 2016 May 23. PMID: 27216828.
10. Chong MY, Wang WC, Hsieh WC, Lee CY, Chiu NM, Yeh WC, Huang OL, Wen JK, Chen CL. Psychological impact of severe acute respiratory syndrome on health workers in a tertiary hospital. Br J Psychiatry. 2004 Aug;185:127-33. doi: 10.1192/bjp.185.2.127. PMID: 15286063.
11. De Brier N, Stroobants S, Vandekerckhove P, De Buck E. Factors affecting mental health of health care workers during coronavirus disease outbreaks (SARS, MERS & COVID-19): A rapid systematic review. PLoS One. 2020 Dec 15;15(12):e0244052. doi: 10.1371/journal.pone.0244052. PMID: 33320910; PMCID: PMC7737991.
12. DiGiovanni C, Conley J, Chiu D, Zaborski J. Factors influencing compliance with quarantine in Toronto during the 2003 SARS outbreak. Biosecur Bioterror. 2004;2(4):265-72. doi: 10.1089/bsp.2004.2.265. PMID: 15650436.
13. Goulia P, Mantas C, Dimitroula D, Mantis D, Hyphantis T. General hospital staff worries, perceived sufficiency of information and associated psychological distress during the A/H1N1 influenza

pandemic. BMC Infect Dis. 2010 Nov 9;10:322. doi: 10.1186/1471-2334-10-322. PMID: 21062471; PMCID: PMC2990753.

14. Grace SL, Hershenfield K, Robertson E, Stewart DE. The occupational and psychosocial impact of SARS on academic physicians in three affected hospitals. Psychosomatics. 2005 Sep-Oct;46(5):385-91. doi: 10.1176/appi.psy.46.5.385. PMID: 16145182; PMCID: PMC7118753.
15. Kisely S, Warren N, McMahon L, Dalais C, Henry I, Siskind D. Occurrence, prevention, and management of the psychological effects of emerging virus outbreaks on healthcare workers: rapid review and meta-analysis. BMJ. 2020 May 5;369:m1642. doi: 10.1136/bmj.m1642. PMID: 32371466; PMCID: PMC7199468.
16. Koh D, Lim MK, Chia SE, Ko SM, Qian F, Ng V, Tan BH, Wong KS, Chew WM, Tang HK, Ng W, Muttakin Z, Emmanuel S, Fong NP, Koh G, Kwa CT, Tan KB, Fones C. Risk perception and impact of Severe Acute Respiratory Syndrome (SARS) on work and personal lives of healthcare workers in Singapore: what can we learn? Med Care. 2005 Jul;43(7):676-82. doi: 10.1097/01.mlr.0000167181.36730.cc. PMID: 15970782.
17. Koh Y, Hegney DG, Drury V. Comprehensive systematic review of healthcare workers' perceptions of risk and use of coping strategies towards emerging respiratory infectious diseases. Int J Evid Based Healthc. 2011 Dec;9(4):403-19. doi: 10.1111/j.1744-1609.2011.00242.x. PMID: 22093389.
18. Maunder R. The experience of the 2003 SARS outbreak as a traumatic stress among frontline healthcare workers in Toronto: lessons learned. Philos Trans R Soc Lond B Biol Sci. 2004 Jul 29;359(1447):1117-25. doi: 10.1098/rstb.2004.1483. PMID: 15306398; PMCID: PMC1693388.
19. Maunder R, Hunter J, Vincent L, Bennett J, Peladeau N, Leszcz M, Sadavoy J, Verhaeghe LM, Steinberg R, Mazzulli T. The immediate psychological and occupational impact of the 2003 SARS outbreak in a teaching hospital. CMAJ. 2003 May 13;168(10):1245-51. PMID: 12743065; PMCID: PMC154178.
20. Muhidin S, Vizheh M, Moghadam ZB. Anticipating COVID-19-related stigma in survivors and health-care workers: Lessons from previous infectious diseases outbreaks - An integrative literature review. Psychiatry Clin Neurosci. 2020 Nov;74(11):617-618. doi: 10.1111/pcn.13140. Epub 2020 Sep 25. PMID: 32889754.
21. Paiano M, Jaques AE, Nacamura PAB, Salci MA, Radovanovic CAT, Carreira L. Mental health of healthcare professionals in China during the new coronavirus pandemic: an integrative review. Rev Bras Enferm. 2020 Sep 18;73(suppl 2):e20200338. English, Portuguese. doi: 10.1590/0034-7167-2020-0338. PMID: 32965402.
22. Park JS, Lee EH, Park NR, Choi YH. Mental Health of Nurses Working at a Government-designated Hospital During a MERS-CoV Outbreak: A Cross-sectional Study. Arch Psychiatr Nurs. 2018 Feb;32(1):2-6. doi: 10.1016/j.apnu.2017.09.006. Epub 2017 Sep 5. PMID: 29413067; PMCID: PMC7127092.
23. Reynolds DL, Garay JR, Deamond SL, Moran MK, Gold W, Styra R. Understanding, compliance and psychological impact of the SARS quarantine experience. Epidemiol Infect. 2008 Jul;136(7):997-1007. doi: 10.1017/S0950268807009156. Epub 2007 Jul 30. PMID: 17662167; PMCID: PMC2870884.
24. Robertson E, Hershenfield K, Grace SL, Stewart DE. The psychosocial effects of being quarantined following exposure to SARS: a qualitative study of Toronto health care workers. Can J Psychiatry. 2004 Jun;49(6):403-7. doi: 10.1177/070674370404900612. PMID: 15283537.
25. Röhr S, Müller F, Jung F, Apfelbacher C, Seidler A, Riedel-Heller SG. Psychosoziale Folgen von Quarantänemaßnahmen bei schwerwiegenden Coronavirus-Ausbrüchen: ein Rapid Review [Psychosocial Impact of Quarantine Measures During Serious Coronavirus Outbreaks: A Rapid Review]. Psychiatr Prax. 2020 May;47(4):179-189. German. doi: 10.1055/a-1159-5562. Epub 2020 Apr 27. PMID: 32340047; PMCID: PMC7295307.
26. Salazar de Pablo G, Vaquerizo-Serrano J, Catalan A, Arango C, Moreno C, Ferre F, Shin JI, Sullivan S, Brondino N, Solmi M, Fusar-Poli P. Impact of coronavirus syndromes on physical and mental health of health care workers: Systematic review and meta-analysis. J Affect Disord. 2020 Oct 1;275:48-57. doi: 10.1016/j.jad.2020.06.022. Epub 2020 Jun 25. PMID: 32658823; PMCID: PMC7314697.
27. Schoch-Spana M, Bouri N, Rambhia KJ, Norwood A. Stigma, health disparities, and the 2009 H1N1 influenza pandemic: how to protect Latino farmworkers in future health emergencies. Biosecur Bioterror. 2010 Sep;8(3):243-54. doi: 10.1089/bsp.2010.0021. PMID: 20825335.
28. Serrano-Ripoll MJ, Meneses-Echavez JF, Ricci-Cabello I, Fraile-Navarro D, Fiol-deRoque MA, Pastor-Moreno G, Castro A, Ruiz-Pérez I, Zamanillo Campos R, Gonçalves-Bradley DC. Impact of viral

epidemic outbreaks on mental health of healthcare workers: a rapid systematic review and meta-analysis. J Affect Disord. 2020 Dec 1;277:347-357. doi: 10.1016/j.jad.2020.08.034. Epub 2020 Aug 23. PMID: 32861835; PMCID: PMC7443314.

29. Shiao JS, Koh D, Lo LH, Lim MK, Guo YL. Factors predicting nurses' consideration of leaving their job during the SARS outbreak. Nurs Ethics. 2007 Jan;14(1):5-17. doi: 10.1177/0969733007071350. PMID: 17334166.
30. Sritharan J, Jegathesan T, Vimaleswaran D, Sritharan A. Mental Health Concerns of Frontline Workers During the COVID-19 Pandemic: A Scoping Review. Glob J Health Sci. 2020;12(11). doi: 10.5539/gjhs.v12n11p89.
31. Tam CW, Pang EP, Lam LC, Chiu HF. Severe acute respiratory syndrome (SARS) in Hong Kong in 2003: stress and psychological impact among frontline healthcare workers. Psychol Med. 2004 Oct;34(7):1197-204. doi: 10.1017/s0033291704002247. PMID: 15697046.
32. Tng XJJ, Chew QH, Sim K. Psychological sequelae within different populations during the COVID-19 pandemic: a rapid review of extant evidence. Singapore Med J. 2020 Jul 30. doi: 10.11622/smedj.2020111. Epub ahead of print. PMID: 32729312.
33. Verma S, Mythily S, Chan YH, Deslypere JP, Teo EK, Chong SA. Post-SARS psychological morbidity and stigma among general practitioners and traditional Chinese medicine practitioners in Singapore. Ann Acad Med Singap. 2004 Nov;33(6):743-8. PMID: 15608831.
34. Yiwen K, Hegney D, Drury V. A comprehensive systematic review of healthcare workers' perceptions of risk from exposure to emerging acute respiratory infectious diseases and the perceived effectiveness of strategies used to facilitate healthy coping in acute hospital and community healthcare settings. JBI Libr Syst Rev. 2010;8(23):917-971. doi: 10.11124/01938924-201008230-00001. PMID: 27819952.

Outcome (n = 30)

1. Adesegun OA, Binuyo T, Adeyemi O, Ehioghae O, Rabor DF, Amusan O, Akinboboye O, Duke OF, Olafimihan AG, Ajose O, Idowu AO, Abiodun O. The COVID-19 Crisis in Sub-Saharan Africa: Knowledge, Attitudes, and Practices of the Nigerian Public. Am J Trop Med Hyg. 2020 Nov;103(5):1997-2004. doi: 10.4269/ajtmh.20-0461. PMID: 32975179; PMCID: PMC7646756.
2. Alahmad B, Kurdi H, Colonna K, Gasana J, Agnew J, Fox MA. COVID-19 stressors on migrant workers in Kuwait: cumulative risk considerations. BMJ Glob Health. 2020 Jul;5(7):e002995. doi: 10.1136/bmjgh-2020-002995. PMID: 32641292; PMCID: PMC7348320.
3. Alhaj AK, Al-Saadi T, Mohammad F, Alabri S. Neurosurgery Residents' Perspective on COVID-19: Knowledge, Readiness, and Impact of this Pandemic. World Neurosurg. 2020 Jul;139:e848-e858. doi: 10.1016/j.wneu.2020.05.087. Epub 2020 May 16. PMID: 32426064; PMCID: PMC7229445.
4. Aziminia N, Khani A, Smith C, Bakhai A, Lisk C. Hospital trainees' worries, perceived sufficiency of information and reported psychological health during the COVID-19 pandemic. Acute Med. 2021;20(1):25-36. PMID: 33749692.
5. Ben Abdelaziz A, Benzarti S, Achouri MY, Nouira S, Mlouki I, Yahia F, Ben Abdelaziz A, Barhoumi T, Quessar A, Ben Salem K. Counter-COVID- 19 pandemic strategy in the Maghreb Central. Qualitative study of the perceptions of health professionals. Tunis Med. 2020 Apr;98(4):266-282. PMID: 32395789.
6. Ben Abdelaziz A, Benzarti S, Nouira S, Mlouki I, Achouri MY, Ben Abdelaziz I, Yahia F, Barhoumi T, Soulimane A. Attitudes of health professionals towards the response to the COVID-19 pandemic in Maghreb. Tunis Med. 2020 May;98(5):324-333. PMID: 32548842.
7. Caruana EJ, Patel A, Kendall S, Rathinam S. Impact of coronavirus 2019 (COVID-19) on training and well-being in subspecialty surgery: A national survey of cardiothoracic trainees in the United Kingdom. J Thorac Cardiovasc Surg. 2020 Oct;160(4):980-987. doi: 10.1016/j.jtcvs.2020.05.052. Epub 2020 Jun 1. PMID: 32605730; PMCID: PMC7262521.
8. Chan, K. L., Chau, W. W., Kuriansky, J., Dow, E., Zinsou, J. C., Leung, J., & Kim, S. (2016). The psychosocial and interpersonal impact of the SARS epidemic on Chinese health professionals: Implications for epidemics, including Ebola. In J. Kuriansky (Ed.), The psychosocial aspects of a deadly epidemic: What Ebola has taught us about holistic healing (pp. 271–286). Praeger, 287-317.
9. Chen CS, Wu HY, Yang P, Yen CF. Psychological distress of nurses in Taiwan who worked during the outbreak of SARS. Psychiatr Serv. 2005 Jan;56(1):76-9. doi: 10.1176/appi.ps.56.1.76. PMID: 15637196.

10. Chung BP, Wong TK, Suen ES, Chung JW. SARS: caring for patients in Hong Kong. J Clin Nurs. 2005 Apr;14(4):510-7. doi: 10.1111/j.1365-2702.2004.01072.x. PMID: 15807759.
11. Fan J, Hu K, Li X, Jiang Y, Zhou X, Gou X, Li X. A qualitative study of the vocational and psychological perceptions and issues of transdisciplinary nurses during the COVID-19 outbreak. Aging (Albany NY). 2020 Jul 3;12(13):12479-12492. doi: 10.18632/aging.103533. Epub 2020 Jul 3. PMID: 32620713; PMCID: PMC7377893.
12. Das, A. K., Srivastava, A. K., Ghosh, S., Bhargava, R., Gupt R. K., & Singh, R. R. (2020). The adequacy of health system measures in reducing vulnerability to COVID 19 among the health care providers working in primary health care in Rajasthan, India A Cross sectional Study. Medrxiv. doi:10.1101/2020.07.21.20149443.
13. Fuchs A, Abegglen S, Berger-Estilita J, Greif R, Eigenmann H. Distress and resilience of healthcare professionals during the COVID-19 pandemic (DARVID): study protocol for a mixed-methods research project. BMJ Open. 2020 Jul 31;10(7):e039832. doi: 10.1136/bmjopen-2020-039832. PMID: 32737101; PMCID: PMC7397979.
14. Hamed A, Latif, Harris M, Patel S, Patel M. et al. (2020). The impact of keeping a religious beard in the COVID-19 pandemic: an online cross sectional survey study exploring experiences of male medical healthcare professionals. medRxiv. doi:10.1101/2020.11.01.20224022.
15. Huxley B, Majerova J, Kovacova M. Prolonged Stress, Anxiety, and Depression in Frontline Medical Staff Working with COVID-19 Patients. Psychosociological Issues in Human Resource Management. 2020; 8(2): 77-86. doi:10.22381/PIHRM8220208.
16. Labrague LJ. Psychological resilience, coping behaviours and social support among health care workers during the COVID-19 pandemic: A systematic review of quantitative studies. J Nurs Manag. 2021 Apr 12. doi: 10.1111/jonm.13336. Epub ahead of print. PMID: 33843087.
17. Mamun MA, Ullah I. COVID-19 suicides in Pakistan, dying off not COVID-19 fear but poverty? - The forthcoming economic challenges for a developing country. Brain Behav Immun. 2020 Jul;87:163-166. doi: 10.1016/j.bbi.2020.05.028. Epub 2020 May 11. PMID: 32407859; PMCID: PMC7212955.
18. Mira JJ, Vicente MA, Lopez-Pineda A, Carrillo I, Guilabert M, Fernández C, Pérez-Jover V, Martin Delgado J, Pérez-Pérez P, Cobos Vargas A, Astier-Peña MP, Martínez-García OB, Marco-Gómez B, Abad Bouzán C. Preventing and Addressing the Stress Reactions of Health Care Workers Caring for Patients With COVID-19: Development of a Digital Platform (Be + Against COVID). JMIR Mhealth Uhealth. 2020 Oct 5;8(10):e21692. doi: 10.2196/21692. PMID: 32936769; PMCID: PMC7537725.
19. Nelson, S., & Byng, A. (2015). The SARS pandemic in Toronto, Canada, 2003. In: "Nurses and Disasters". In A. W. Keeling & B. M. Wall (Eds.), Nurses and Disasters. Global, Hostorical Case Studies. Springer Publishing Company. doi: 10.1891/9780826126733.0009
20. Ng BH, Nuratiqah NA, Faisal AH, Soo CI, Low HJ, Najma K, Periyasamy P, Mustafa N, Andrea YLB. A descriptive study of the psychological experience of health care workers in close contact with a person with COVID-19. Med J Malaysia. 2020 Sep;75(5):485-489. PMID: 32918414.
21. Palacios-Ceña D, Fernández-de-Las-Peñas C, Florencio LL, de-la-Llave-Rincón AI, Palacios-Ceña M. Emotional Experience and Feelings during First COVID-19 Outbreak Perceived by Physical Therapists: A Qualitative Study in Madrid, Spain. Int J Environ Res Public Health. 2020 Dec 27;18(1):127. doi: 10.3390/ijerph18010127. PMID: 33375405; PMCID: PMC7795029.
22. Pedrozo-Pupo JC, Pedrozo-Cortés MJ, Campo-Arias A. Perceived stress associated with COVID-19 epidemic in Colombia: an online survey. Cad Saude Publica. 2020;36(5):e00090520. doi: 10.1590/0102-311x00090520. Epub 2020 Jun 1. PMID: 32490918.
23. Phua DH, Tang HK, Tham KY. Coping responses of emergency physicians and nurses to the 2003 severe acute respiratory syndrome outbreak. Acad Emerg Med. 2005 Apr;12(4):322-8. doi: 10.1197/j.aem.2004.11.015. PMID: 15805323.
24. Poonian J, Walsham N, Kilner T, Bradbury E, Brooks K, West E. Managing healthcare worker well-being in an Australian emergency department during the COVID-19 pandemic. Emerg Med Australas. 2020 Aug;32(4):700-702. doi: 10.1111/1742-6723.13547. Epub 2020 Jun 25. PMID: 32386263; PMCID: PMC7272835.
25. Sethi BA, Sethi A, Ali S, Aamir HS. Impact of Coronavirus disease (COVID-19) pandemic on health professionals. Pak J Med Sci. 2020 May;36(COVID19-S4):S6-S11. doi: 10.12669/pjms.36.COVID19-S4.2779. PMID: 32582306; PMCID: PMC7306959.

26. Shechter A, Diaz F, Moise N, Anstey DE, Ye S, Agarwal S, Birk JL, Brodie D, Cannone DE, Chang B, Claassen J, Cornelius T, Derby L, Dong M, Givens RC, Hochman B, Homma S, Kronish IM, Lee SAJ, Manzano W, Mayer LES, McMurry CL, Moitra V, Pham P, Rabbani L, Rivera RR, Schwartz A, Schwartz JE, Shapiro PA, Shaw K, Sullivan AM, Vose C, Wasson L, Edmondson D, Abdalla M. Psychological distress, coping behaviors, and preferences for support among New York healthcare workers during the COVID-19 pandemic. Gen Hosp Psychiatry. 2020 Sep-Oct;66:1-8. doi: 10.1016/j.genhosppsych.2020.06.007. Epub 2020 Jun 16. PMID: 32590254; PMCID: PMC7297159.
27. Silczuk A. Threatening increase in alcohol consumption in physicians quarantined due to coronavirus outbreak in Poland: the ALCOVID survey. J Public Health (Oxf). 2020 Aug 18;42(3):461-465. doi: 10.1093/pubmed/fdaa110. PMID: 32643751; PMCID: PMC7454802.
28. Wang Y, Duan Z, Peng K, Li D, Ou J, Wilson A, Wang N, Si L, Chen R. Acute stress disorder among frontline health professionals during the COVID-19 outbreak: A structural equation modelling investigation. Psychosom Med. 2020 Aug 17. doi: 10.1097/PSY.0000000000000851. Epub ahead of print. PMID: 32815855.
29. Wang LQ, Zhang M, Liu GM, Nan SY, Li T, Xu L, Xue Y, Zhang M, Wang L, Qu YD, Liu F. Psychological impact of coronavirus disease (2019) (COVID-19) epidemic on medical staff in different posts in China: A multicenter study. J Psychiatr Res. 2020 Oct;129:198-205. doi: 10.1016/j.jpsychires.2020.07.008. Epub 2020 Jul 14. PMID: 32763586; PMCID: PMC7834267.
30. Xie H, Cheng X, Song X, Wu W, Chen J, Xi Z, Shou K. Investigation of the Psychological disorders in the healthcare nurses during a coronavirus disease 2019 outbreak in China. Medicine (Baltimore). 2020 Aug 21;99(34):e21662. doi: 10.1097/MD.0000000000021662. PMID: 32846780; PMCID: PMC7447415.

Population (n = 10)

1. Alkhamees AA, Alrashed SA, Alzunaydi AA, Almohimeed AS, Aljohani MS. The psychological impact of COVID-19 pandemic on the general population of Saudi Arabia. Compr Psychiatry. 2020 Oct;102:152192. doi: 10.1016/j.comppsych.2020.152192. Epub 2020 Jul 12. PMID: 32688022; PMCID: PMC7354380.
2. Brondani M, Donnelly L. The HIV and SARS-CoV-2 Parallel in Dentistry from the Perspectives of the Oral Health Care Team. JDR Clin Trans Res. 2021 Jan;6(1):40-46. doi: 10.1177/2380084420961089. Epub 2020 Sep 18. PMID: 32942933; PMCID: PMC7502681.
3. Duan W, Bu H, Chen Z. COVID-19-related stigma profiles and risk factors among people who are at high risk of contagion. Soc Sci Med. 2020 Dec;266:113425. doi: 10.1016/j.socscimed.2020.113425. Epub 2020 Oct 7. PMID: 33059301; PMCID: PMC7540249.
4. Earnshaw VA, Brousseau NM, Hill EC, Kalichman SC, Eaton LA, Fox AB. Anticipated stigma, stereotypes, and COVID-19 testing. Stigma and Health. 2020;5(4):390-393. doi: 10.1037/sah0000255
5. Gardner PJ, Moallef P. Psychological impact on SARS survivors: Critical review of the English language literature. Canadian Psychology/Psychologie canadienne. 2015;56(1):123-135. doi: 10.1037/a0037973.
6. Hao F, Tam W, Hu X, Tan W, Jiang L, Jiang X, Zhang L, Zhao X, Zou Y, Hu Y, Luo X, McIntyre RS, Quek T, Tran BX, Zhang Z, Pham HQ, Ho CSH, Ho RCM. A quantitative and qualitative study on the neuropsychiatric sequelae of acutely ill COVID-19 inpatients in isolation facilities. Transl Psychiatry. 2020 Oct 19;10(1):355. doi: 10.1038/s41398-020-01039-2. PMID: 33077738; PMCID: PMC7570419.
7. Lee S, Chan LY, Chau AM, Kwok KP, Kleinman A. The experience of SARS-related stigma at Amoy Gardens. Soc Sci Med. 2005 Nov;61(9):2038-46. doi: 10.1016/j.socscimed.2005.04.010. PMID: 15913861; PMCID: PMC7116975.
8. Siu JY. The SARS-associated stigma of SARS victims in the post-SARS era of Hong Kong. Qual Health Res. 2008 Jun;18(6):729-38. doi: 10.1177/1049732308318372. PMID: 18503014.
9. Sorokin MY, Kasyanov ED, Rukavishnikov GV, Makarevich OV, Neznanov NG, Morozov PV, Lutova NB, Mazo GE. Stress and Stigmatization in Health-Care Workers during the COVID-19 Pandemic. Indian J Psychiatry. 2020 Sep;62(Suppl 3):S445-S453. doi: 10.4103/psychiatry.IndianJPsychiatry_870_20. Epub 2020 Sep 28. PMID: 33227056; PMCID: PMC7659791.

10. Sun N, Wei L, Wang H, Wang X, Gao M, Hu X, Shi S. Qualitative study of the psychological experience of COVID-19 patients during hospitalization. J Affect Disord. 2021 Jan 1;278:15-22. doi: 10.1016/j.jad.2020.08.040. Epub 2020 Aug 24. PMID: 32949869; PMCID: PMC7444461.

No thematic reference (n = 5)

1. Albott CS, Wozniak JR, McGlinch BP, Wall MH, Gold BS, Vinogradov S. Battle Buddies: Rapid Deployment of a Psychological Resilience Intervention for Health Care Workers During the COVID-19 Pandemic. Anesth Analg. 2020 Jul;131(1):43-54. doi: 10.1213/ANE.0000000000004912. PMID: 32345861; PMCID: PMC7199769.
2. Blank, C. Pharmacists feel overworked, face more discrimination: Survey results reveal improvements and challenges in the workplace. Drug Topics. 2020;164(6).
3. Janyam S, Phuengsamran D, Pangnongyang J, Saripra W, Jitwattanapataya L, Songsamphan C, Benjarattanaporn P, Gopinath D. Protecting sex workers in Thailand during the COVID-19 pandemic: opportunities to build back better. WHO South East Asia J Public Health. 2020 Sep;9(2):100-103. doi: 10.4103/2224-3151.294301. PMID: 32978340.
4. Teo P, Yeoh BS, Ong SN. SARS in Singapore: surveillance strategies in a globalising city. Health Policy. 2005 Jun;72(3):279-91. doi: 10.1016/j.healthpol.2004.11.004. Epub 2004 Dec 7. PMID: 15862636; PMCID: PMC7132468.
5. Abdel Wahed WY, Hefzy EM, Ahmed MI, Hamed NS. Assessment of Knowledge, Attitudes, and Perception of Health Care Workers Regarding COVID-19, A Cross-Sectional Study from Egypt. J Community Health. 2020 Dec;45(6):1242-1251. doi: 10.1007/s10900-020-00882-0. PMID: 32638199; PMCID: PMC7340762.

Not available (n= 3)

1. Beyan AC, Demiral Y, Erdal S. Occupational diseases as a metaphor: do not cough! Eur Respir J. 2015;52(suppl 62).
2. MenkinSmith L, Catchpole K, Reves JG. Adapting Ebola training to educate healthcare workers during the SARS-2-CoV pandemic. Am J Disaster Med. 2020 Spring;15(2):137-140. doi: 10.5055/ajdm.2020.0363. PMID: 32804393.
3. Xue Y. A comprehensive systematic review of healthcare workers' perceptions of risk from exposure to emerging acute respiratory infectious diseases and the perceived effectiveness of strategies used to facilitate healthy coping in acute hospital and community healthcare settings. Journal of Advanced Nursing. 2011;67(2):270-272.

Exposition (n= 2)

1. Abuhammad S, H Alzoubi K, Khabour O. Fear of COVID-19 and stigmatization towards infected people among Jordanian people. Int J Clin Pract. 2021 Apr;75(4):e13899. doi: 10.1111/ijcp.13899. Epub 2020 Dec 18. PMID: 33280204; PMCID: PMC7883188.
2. Baldassarre A, Giorgi G, Alessio F, Lulli LG, Arcangeli G, Mucci N. Stigma and Discrimination (SAD) at the Time of the SARS-CoV-2 Pandemic. Int J Environ Res Public Health. 2020 Aug 31;17(17):6341. doi: 10.3390/ijerph17176341. PMID: 32878180; PMCID: PMC7503800.

Others (n= 2)

Poster presentation:

1. Farag E, nour M, Marufu O, Sikkema R, Al-Romaihi H, Al Thani M, Al-Marri S, El-Sayed A, Reusken CBEM, Al-Hajri M, Koopmans MPG. The hidden epidemic: MERS-CoV-related stigma observations from the field, Qatar 2012-2015. Int J Infect Dis. 2016 Apr;45:332. doi: 10.1016/j.ijid.2016.02.719. Epub 2016 Mar 29. PMCID: PMC7129574.

Duplicate:

2. Choi JS, Kim JS. Factors influencing emergency nurses' ethical problems during the outbreak of MERS-CoV. Nurs Ethics. 2018 May;25(3):335-345. doi: 10.1177/0969733016648205. Epub 2016 May 23. PMID: 27216828.
