# Supplementary material for: Stigmatization from Work-Related COVID-19 Exposure: A Systematic Review with Meta-Analysis
Source: Int J Environ Res Public Health. 2021 Jun 8;18(12):6183. doi: 10.3390/ijerph18126183 (PMC8226580; doi:10.3390/ijerph18126183)
Supplement: Supplementary file 1 [file ijerph-18-06183-s001.zip › Supplementary_ijerph-1235285/Table_S3.pdf]

General	Study	Population	Exposure	Outcome	Results Stigmatization as outcome:<br><br>Descriptive summary (qualitative/ exploratory studies)	Results Stigmatization as outcome:<br><br>Quantitative (meta-analytical) results (prevalence/ Incidence, Relative Risk, Odds Ratio)	Effect of stigmatization on health<br><br>Outcome:	Results Effect of stigmatization on health:<br><br>Relative risk (RR)/ Odds Ratio (OR)/ or descriptive summary	Strengths (+), weaknesses (-), confounding, other bias, over- or under-estimation of potential effects
**#<br>First Author, Year**	**Search period:<br>Inclusion criteria:<br>Number of (all) included studies in review: n=<br>Number of studies on stigmatization due to COVID-19 included: n= (total, cohort, case-control, cross-sectional)**	**Job description:<br>Inclusion criteria:<br>Reference group:**	*Refers to the circumstances when stigmatization was studied (lockdown, social isolation)*	*Describe main topic of review*<br><br>**Form of stigmatization**<br><br>*Describe the form of stigmatization and how it was measured*	*Include a descriptive summary of important results if appropriate*	*Describe the prevalence/ incidence of stigmatization*	*Describe the health outcome and how it was measured*	*Include (if possible) the risk estimates for the effect of stigmatization on health outcome*<br>*If there is no risk estimate, give a descriptive summary*	
#7183<br>Banerjee, 2020	**Search period:** till June 2020<br>**Inclusion/ criteria:**<br>- sample sizes ≥10 participants<br>- studied prevalence/ surveys of psychological or psychosocial problems in South-Asian (World Psychi-	**Job description:** HCWs and general population<br>**Inclusion criteria:** working in close proximity with infected patients, aged 18–60 years	Not specified<br><br>(during outbreak of COVID-19)	Psychosocial health and well-being<br>**Form of stigmatization**<br>Stigma and discrimination (not specified)	“Frontline workers reported guilt, stigma, anxiety, and poor sleep quality, which were related to the lack of availability of adequate personal protective equipment, increased workload, and discrimination	-	Stress	“Stigma and discrimination against the frontline workers were identified as important factors contributing to their stress.”<br><br>See extraction of Chatterjee et al. 2020	+ Funding stated (none)<br>+ No conflict of interest declared

General	Study	Population	Exposure	Outcome	Results Stigmatization as outcome:<br><br>Descriptive summary (qualitative/ exploratory studies)	Results Stigmatization as outcome:<br><br>Quantitative (meta-analytical) results (prevalence/ Incidence, Relative Risk, Odds Ratio)	Effect of stigmatization on health<br><br>Outcome:	Results Effect of stigmatization on health:<br><br>Relative risk (RR)/ Odds Ratio (OR)/ or descriptive summary	Strengths (+), weaknesses (-), confounding, other bias, over- or under-estimation of potential effects
	atric Association Zone 16) countries,<br>- investigated prevalence/ incidence of psychiatric disorders in general population/ frontline workers/ COVID-19 affected patients as a consequence of COVID-19 pandemic<br>- used study population of adults age-ranged from ≥18 years, and<br>- reported significant differences in the studied parameters<br>**Number of (all) included studies in review:**<br>n=13	**Reference group:**<br>n.a.			See extraction of #Chatterjee et al. 2020				

General	Study	Population	Exposure	Outcome	Results Stigmatization as outcome:<br><br>Descriptive summary (qualitative/ exploratory studies)	Results Stigmatization as outcome:<br><br>Quantitative (meta-analytical) results (prevalence/ Incidence, Relative Risk, Odds Ratio)	Effect of stigmatization on health<br><br>Outcome:	Results Effect of stigmatization on health:<br><br>Relative risk (RR)/ Odds Ratio (OR)/ or descriptive summary	Strengths (+), weaknesses (-), confounding, other bias, over- or underestimation of potential effects
	**Number of studies on stigmatization due to COVID-19 included:** n=1 (cross-sectional)								
#780 Cabarkapa, 2020	**Search period:** 2002 – 21 August 2020<br>**Inclusion/ criteria:** Original research, published in peer-reviewed journals, studies reporting mental health or psychological well-being in HCW -studies with a sample size of at least n=100<br>**Number of (all) included studies in review:** n=55	**Job description:** HCW<br>**Inclusion criteria:** working in close proximity with infected patients<br>**Reference group:** n.a.	Not specified<br><br>(During severe viral outbreaks (i.e. COVID-19, SARS, MERS, Ebola, and Influenza H1N1))	Psychological impact related to working with patients with infectious viral diseases<br><br>**Form of stigmatization** Stigma, not specified	"Stigma was a major factor"<br><br>See extraction of #759 Juan et al. 2020	-	Depression	"Higher risk of depressive symptoms due to stigmatization"<br><br>See extraction of #759 Juan et al. 2020	+ Funding stated (none)<br><br>Unclear risk of bias: the authors declared no conflicts of interest regarding authorship or the publication, but one author received commercial funding

General	Study	Population	Exposure	Outcome	Results Stigmatization as outcome:<br><br>Descriptive summary (qualitative/ exploratory studies)	Results Stigmatization as outcome:<br><br>Quantitative (meta-analytical) results (prevalence/ Incidence, Relative Risk, Odds Ratio)	Effect of stigmatization on health<br><br>Outcome:	Results Effect of stigmatization on health:<br><br>Relative risk (RR)/ Odds Ratio (OR)/ or descriptive summary	Strengths (+), weaknesses (-), confounding, other bias, over- or underestimation of potential effects
	**Number of studies on stigmatization due to COVID-19 included:** n=1 (cross-sectional)								
#7283 Callus, 2020	**Search period:** 2000 until June 2020 **Inclusion/ criteria:** empirical quantitative and qualitative studies in which relaxation techniques of various types implemented on health care providers caring for patients during severe coronavirus pandemics and articles that consider the implementation of mental	**Job description:** Health care providers **Inclusion criteria:** dealing with patients infected with severe coronavirus (SARS, MERS, and COVID-19) **Reference group:** n.a.	Stress reduction technique, model, or recommendation (relaxation techniques of various types) for health care providers dealing with patients infected with severe coronavirus (SARS, MERS, and COVID-19), best practices and interventions	Reduction of psychological distress (e.g., anxiety, depression, PTSD, burnout) and mental health outcomes (additionally, delivery mechanisms of the identified interventions, the instruments used to test their efficacy, the determinants of their effectiveness, and their im-	**Study by Blake et al. 2020:**<br>- reports the development and evaluation of a digital package, which – amongst others –outlines the actions that team leaders can undertake to provide a guide to reduce social stigma	-	-	-	+ partial funding from a public source (Ricerca Corrente funding from the Italian Ministry of Health)<br>+ no conflict of interest declared

General	Study	Population	Exposure	Outcome	Results Stigmatization as outcome:<br><br>Descriptive summary (qualitative/ exploratory studies)	Results Stigmatization as outcome:<br><br>Quantitative (meta-analytical) results (prevalence/ Incidence, Relative Risk, Odds Ratio)	Effect of stigmatization on health<br><br>Outcome:	Results Effect of stigmatization on health:<br><br>Relative risk (RR)/ Odds Ratio (OR)/ or descriptive summary	Strengths (+), weaknesses (-), confounding, other bias, over- or underestimation of potential effects
	health care services considered to be pertinent, such as commentaries<br>**Number of (all) included studies in review:** n=14<br>**Number of studies on stigmatization due to COVID-19 included:** n=1 (total)<br>n=1 (intervention study)			pact on specific psychological variables)<br><br>**Form of stigmatization**<br>Stigma, not specified					
#7274 Joo 2021	**Search period:** until August 31 2020<br>**Inclusion/ criteria:** Qualitative studies on registered nurses caring for COVID-19 patients, published in English, peer-re-	**Job description:** registered nurses<br>**Inclusion criteria:** caring for COVID-19 patients<br>**Reference group:** n.a.	Caring for COVID-19 patients	Experiences/ barriers of nurses caring for COVID-19 patients<br><br>**stigmatization**<br>Stigma, not specified	Not reported in detail<br><br>For details see<br><br>#1033 Kackin et al. 2020<br><br>#7289 Sadati et al. 2021	-	-	-	+ Funding stated (none)<br>+ no conflict of interest declared

General	Study	Population	Exposure	Outcome	Results Stigmatization as outcome:<br><br>Descriptive summary (qualitative/ exploratory studies)	Results Stigmatization as outcome:<br><br>Quantitative (meta-analytical) results (prevalence/ Incidence, Relative Risk, Odds Ratio)	Effect of stigmatization on health<br><br>Outcome:	Results Effect of stigmatization on health:<br><br>Relative risk (RR)/ Odds Ratio (OR)/ or descriptive summary	Strengths (+), weaknesses (-), confounding, other bias, over- or under-estimation of potential effects
	viewed, published/ or in publication between January-August 2020<br>**Number of (all) included studies in review:** n=9<br>**Number of studies on stigmatization due to COVID-19 included:** n=2 (qualitative)								
#7278 Muller, 2020	**Search period:** until May 11 2020<br>**Inclusion/ criteria:** any type of study (quantitative studies examining prevalence of problems and effects of interventions as well as qualitative studies examining experi-	**Job description:** healthcare workers<br>**Inclusion criteria:** any type of healthcare worker during the COVID-19 pandemic<br>**Reference group:** n.a.	- COVID-19 pandemic<br>- interventions aimed at preventing or reducing negative mental health impacts	Mental health impact<br><br>**stigmatization**<br>Perceived stigma	"Healthcare workers also reported that they were stigmatized, because they were potential sources of infection"<br><br>See extraction of #7285 Mohindra et al. 2020	-	-	-	+ no funding<br>+ no conflict of interest declared<br>+ study protocol available online

General	Study	Population	Exposure	Outcome	Results Stigmatization as outcome:<br><br>Descriptive summary (qualitative/ exploratory studies)	Results Stigmatization as outcome:<br><br>Quantitative (meta-analytical) results (prevalence/ Incidence, Relative Risk, Odds Ratio)	Effect of stigmatization on health<br><br>Outcome:	Results Effect of stigmatization on health:<br><br>Relative risk (RR)/ Odds Ratio (OR)/ or descriptive summary	Strengths (+), weaknesses (-), confounding, other bias, over- or underestimation of potential effects
	ences), no restrictions related to study design, methodological quality, or language<br>**Number of (all) included studies in review:** n=59<br>**Number of studies on stigmatization due to COVID-19 included:** n=1 (total)<br>n=1 (cross-sectional)								
#7275 Rahman, 2021	**Search period:** December 2019 - July 2020<br>**Inclusion/ criteria:** empirical studies on human rights violations, social stigma and discriminatory behaviors against people of certain ethnic	**Job description:** HCW<br>**Inclusion criteria:** none<br>**Reference group:** n.a.	COVID-19 pandemic	Mental distress and human rights violations (mobility rights, quarantine, and lockdown, shortage of supplies and equipment for HCWs, child rights, elderly's rights,	Not reported<br><br>Cross-sectional study: Abdel Wahed WY, Hefzy EM, Ahmed MI, Hamed NS. Assessment of Knowledge, Attitudes, and Perception of Health Care	-	Distress, stress and post-traumatic stress symptoms	higher levels of stigmatization were associated with higher levels of mental distress, suggestions to deal with stigma are made	+ funding stated (none)<br>+ no conflict of interest declared<br><br>-no quality assessment of studies

General	Study	Population	Exposure	Outcome	Results Stigmatization as outcome: Descriptive summary (qualitative/ exploratory studies)	Results Stigmatization as outcome: Quantitative (meta-analytical) results (prevalence/ Incidence, Relative Risk, Odds Ratio)	Effect of stigmatization on health Outcome:	Results Effect of stigmatization on health: Relative risk (RR)/ Odds Ratio (OR)/ or descriptive summary	Strengths (+), weaknesses (-), confounding, other bias, over- or underestimation of potential effects
	backgrounds, HCWs, and anyone perceived to have been in contact with the virus. Mental, social, and behavioral health outcomes of the violations according to DSM/ICD classifications diagnostic categories or as ascertained using psychometric instruments. Protocols and reports including guidelines by international agencies vetted by scientific and peer reviewers which address one of the primary outcomes were included.			and disproportionate impacts on minority rights and psychiatric patients.<br><br>**Form of stigmatization**<br>Stigma, not specified	Workers Regarding COVID-19, A Cross-Sectional Study from Egypt. J Community Health. 2020 Dec;45(6):1242-1251. doi: 10.1007/s10900-020-00882-0. PMID: 32638199; PMCID: PMC7340762 Was excluded by us for the reason that HCWs were asked for their opinion/concerns/fear what might happen if getting infected with COVID-19<br><br>Qualitative study: see extraction of #910				

General	Study	Population	Exposure	Outcome	Results Stigmatization as outcome:<br><br>Descriptive summary (qualitative/ exploratory studies)	Results Stigmatization as outcome:<br><br>Quantitative (meta-analytical) results (prevalence/ Incidence, Relative Risk, Odds Ratio)	Effect of stigmatization on health<br><br>Outcome:	Results Effect of stigmatization on health:<br><br>Relative risk (RR)/ Odds Ratio (OR)/ or descriptive summary	Strengths (+), weaknesses (-), confounding, other bias, over- or underestimation of potential effects
	**Number of (all) included studies in review:** n=24 **Number of studies on stigmatization due to COVID-19 included:** n=6 (total), working population: n=3 (cross-sectional n=1, qualitative: n=1, letter-to-the-editor: n=1)				Fawaz et al. 2020<br><br>Letter-to-the-editor[1]: Chew et al. (2020). Psychological and coping responses to COVID-19 amongst residents in training across ACGME-I accredited specialties in Singapore. *Psychiatry Research*, *290*, 113146 -> Same results as published in Chew et al. 2020 (# 2030), see extraction				

n.a. = not applicable, n.r. = not reported

[1] Letter-to-the-editors were excluded in our rapid scoping review. The same study was published in #2030 Chew et al. 2020
