# Supplementary material for: Stigmatization from Work-Related COVID-19 Exposure: A Systematic Review with Meta-Analysis
Source: Int J Environ Res Public Health. 2021 Jun 8;18(12):6183. doi: 10.3390/ijerph18126183 (PMC8226580; doi:10.3390/ijerph18126183)
Supplement: Supplementary file 1 [file ijerph-18-06183-s001.zip › Supplementary_ijerph-1235285/Table_S4.pdf]

<table>
<tr><th>General</th><th>Study</th><th>Population</th><th>Exposure</th><th>Outcome</th><th>Results</th><th>Comments</th></tr>
<tr><td>#
First Author, Year, Country

Risk of bias</td><td>Study design:
Time of Study:
Follow-up mean, range:</td><td>Job description:
Inclusion criteria:
invited:
baseline:
follow -up: Age at baseline:
F : M at baseline:
Response:
Loss-to-follow-up:</td><td>Describe how exposure was measured</td><td>Describe how outcome was measured

Form of stigmatization:</td><td>Prevalence of stigmatization:

Conditions increasing work-related stigmatization:

Measures to prevent/ deal with stigmatization at work:

Unfavourable health consequences associated with stigmatization:

Stigmatization studied in association with return-to-work:</td><td></td></tr>
<tr><td>7288
Chatterjee, 2020,
India

High risk</td><td>Study design: Cross-sectional
Time of Study:
28 March - 6 April 2020
Follow-up mean, range: n.a.</td><td>Job description: Doctors
Inclusion criteria: none
invited: n.a. convenience sample -> link shared via Email, WhatsApp and other social media
baseline: n=152
follow -up: n.a.
Age at baseline: 42.1 (SD ±12.2) years
% females: 21.7%
Response: n.a. (convenience sample)
Loss-to-follow-up: n.a.</td><td>During COVID-19 pandemic (not specified)</td><td>One question: Have you been ostracized? (yes, no)

Form of stigmatization:
<u>Self-stigma (perceived): social exclusion</u>
“ostracized by friends, neighbor, and the society for working in the hospital for being exposed and spreading others“</td><td>Prevalence of stigmatization:
Yes: n=32 (21.1%)
No: n=120 (78.9%)

Conditions increasing work-related stigmatization: was not investigated

Measures to prevent/ deal with stigmatization at work: was not investigated

Unfavourable health consequences associated with stigmatization: Depression, anxiety, and stress
Results are shown in Tab. 2.

Stigmatization studied in association with return-to-work: was not investigated</td><td>+ no conflict of interest
+ funding stated (none)

- recruitment procedure: convenience sample
-outcome: 1 item
- analysis method
- no ethical approval</td></tr>
<tr><td>2040
Chaudhary, 2020,
Pakistan

High risk</td><td>Study design: Cross-sectional
Time of Study:
March - June 2020</td><td>Job description: Clinical Oral HCWs (dentists and dental assistants/ hygienists) (n=392),
Non-clinical oral HCWs (dental laboratory</td><td>During COVID-19 pandemic (not specified)</td><td>Questionnaire with 4 items concerning stigmatization (1=strongly disagree to 6=strongly agree), dichotomized as “agree” (= strongly agree, agree, and probably agree) and “disagree” (= strongly</td><td>Prevalence of stigmatization:

<u>Self-stigma (anticipated):</u>
Table 4: Perceived impact on personal life and work<table><tr><td>Perceveid impact (agree)</td><td>Clinical, N (%)</td><td>Non-clinical (Reference group), N (%)</td><td>Total, N (%)</td><td>Adjusted OR*</td><td>p-value</td></tr></table></td><td>+ ethical approval for the study protocol was obtained from the Dental College, HITEC Institute of Medical Sciences
+ outcome: use of a valid and reliable questionnaire</td></tr>
</table>

<table>
<tr><th>General</th><th>Study</th><th>Population</th><th>Exposure</th><th>Outcome</th><th>Results</th><th>Comments</th></tr>
<tr>
<td></td>
<td>Follow-up mean, range: n.a.</td>
<td>technicians, attendants/ cleaners, managerial/ clerical services) (n=191) from 10 different dental hospitals
Inclusion criteria: none
invited: n.r.
baseline: n=583
follow -up: n.a.
Age at baseline:
20-29 years: n=124 (31.6%, clinical), n=51 (26.7%, non-clinical),
30-39: 120 (30.6% clinical), 69 (36.1% non-clinical),
40-49: 98 (25.0, clinical), 46 (24.0% non-clinical),
50-60: 50 (12.7% clinical), 25 (13.0% non-clinical)
% females:
54.8% clinical
22.5% non-clinical
Response: n.r.
Loss-to-follow-up:
n.a.</td>
<td></td>
<td>disagree, disagree, and probably disagree)

Form of stigmatization:
<u>Self-stigma (anticipated):</u>
“I would be afraid of telling my family/ friends about the risk I am exposed”, “I would avoid telling other people about the nature of my job”, “People would avoid me because of my job”

<u>Associative Stigma: social exclusion</u>
“People would avoid my family members because of my job”</td>
<td>
<table>
<tr><td>I would be afraid of telling my family/ friends about the risk I am exposed</td><td>336 (85.7)</td><td>134 (70.2)</td><td>470 (80.6)</td><td>2.55 (1.67-3.88)</td><td>.001</td></tr>
<tr><td>People would avoid me because of my job</td><td>351 (89.5)</td><td>139 (72.8)</td><td>490 (84.0)</td><td>3.20 (2.03-5.04)</td><td>.001</td></tr>
<tr><td>I would avoid telling other people about the nature of my job</td><td>347 (88.5)</td><td>135 (70.7)</td><td>482 (82.7)</td><td>3.19 (2.06-4.96)</td><td>.001</td></tr>
</table>
*Adjusted for age, gender, marital status, place of work, staying with family/friends.

<u>Associative Stigma (social exclusion):</u>
Table 4: Perceived impact on personal life and work
<table>
<tr><th>Perceveid impact (agree)</th><th>Clinical, N (%)</th><th>Non-clinical (Reference group), N (%)</th><th>Total, N (%)</th><th>Adjusted OR*</th><th>p-value</th></tr>
<tr><td>People would avoid my family members because of my job</td><td>336 (85.7)</td><td>129 (67.5)</td><td>465 (79.8)</td><td>2.88 (1.90-4.36)</td><td>.001</td></tr>
</table>
*Adjusted for age, gender, marital status, place of work, staying with family/friends.
</td>
<td>modified and adopted for the COVID-19 pandemic, stigmatization measured with 4 questions, usage and validation of an adopted translated Urdu questionnaire to assess concerns, perceived impact, and preparedness of OHCWs in the COVID-19 pandemic (Cronbach's α 0.81=satisfactory)
+ Funding stated (none)
+ confounding
+ analysis method

- recruitment procedure: no detailed information on the recruitment procedure (# invited not reported)
- response rate not shown</td>
</tr>
</table>

<table>
<tr><th>General</th><th>Study</th><th>Population</th><th>Exposure</th><th>Outcome</th><th>Results</th><th>Comments</th></tr>
<tr><td></td><td></td><td></td><td></td><td></td><td>**Conditions increasing work-related stigmatization:** was not investigated

Measures to prevent/ deal with stigmatization at work: was not investigated

Unfavourable health consequences associated with stigmatization: was not investigated

Stigmatization studied in association with return-to-work: was not investigated</td><td></td></tr>
<tr><td>761
Chen, 2020, China

High risk</td><td>**Study design:** Longitudinal name: “Public attitude towards the novel coronavirus pandemic in Hubei”
Time of Study: Baseline: 2 - 8 Feb 2020 Follow-up: 23 March - 9 April 2020
Follow-up mean, range: 2 months</td><td>**Job description:** Government/ public institution/ institutions/state-owned Enterprises (SOE): 32.9%
Private enterprise staff or individual business: 53.2%
No work or refuse to disclose: 13.8%
Inclusion criteria: none
 # invited: n. r.
 # baseline: n=5239
 # follow-up: n=2054 -> final sample: n=1902
Age at baseline: Mean 33.5 (SD ± 12.5) years
% of females: 43.9%
Response: n.r.
Loss-to-follow-up: 60.1%</td><td>COVID-19; Baseline: rapid increase in COVID-19 cases and related deaths; Follow-up: authorities relaxed lowdown on 23 March; officially lifted in 8 April</td><td>Perceived discrimination: one item whether they had encountered discrimination because of the COVID-19 pandemic (dichotomized: yes/ no), not validated

Form of stigmatization:
Self-stigma (perceived): discrimination
”…whether they had encountered discrimination because of the COVID-19 pandemic“</td><td>**Prevalence of stigmatization:**

Self-stigma (perceived):
Baseline: 45%
Follow-up: 43%

Tab. 2: Responses toward COVID-19 (“Perceived discrimination”)
<table>
<tr><td></td><td>Wave 1</td><td></td><td>Wave 2</td><td></td></tr>
<tr><td>*Age*</td><td>%</td><td>Chi^2 test</td><td>%</td><td>Chi^2 test</td></tr>
<tr><td>≤ 25 years</td><td>51.6</td><td>23.12</td><td>47.8</td><td>11.39</td></tr>
<tr><td>26-45 years</td><td>42.9</td><td></td><td>41.7</td><td></td></tr>
<tr><td>≥ 46 years</td><td>35.1</td><td></td><td>36.4</td><td></td></tr>
<tr><td>*Sex*</td><td></td><td></td><td></td><td></td></tr>
<tr><td>Male</td><td>50.0</td><td>5.56</td><td>44.2</td><td>1.57</td></tr>
<tr><td>Female</td><td>38.1</td><td></td><td>40.6</td><td></td></tr>
<tr><td>*Education*</td><td></td><td></td><td></td><td></td></tr>
<tr><td>≤ High school</td><td>42.6</td><td>-1.41</td><td>43.7</td><td>0.85</td></tr>
<tr><td>≥ College</td><td>45.9</td><td></td><td>41.8</td><td></td></tr>
<tr><td>*Job*</td><td></td><td></td><td></td><td></td></tr>
<tr><td>Government/ SOE</td><td>48.1</td><td>2.22</td><td>39.0</td><td>-2.05</td></tr>
<tr><td>Others</td><td>42.1</td><td></td><td>44.5</td><td></td></tr>
</table>

Conditions increasing work-related stigmatization: was not investigated</td><td>+ ethical approval from the ethics committee of the Chinese Academy of Social Sciences (CHN-2153, 18/0020)
+ no conflict of interest
+ chronology: follow-up

- recruitment procedure: no description of recruitment (# of invited not reported)
- outcome: only one item to measure stigmatization
- analysis method:
- funding not stated</td></tr>
</table>

<table>
<tr><th>General</th><th>Study</th><th>Population</th><th>Exposure</th><th>Outcome</th><th>Results</th><th>Comments</th></tr>
<tr><td></td><td></td><td></td><td></td><td></td><td>Measures to prevent/ deal with stigmatization at work: was not investigated

Unfavourable health consequences associated with stigmatization: was not investigated

Stigmatization studied in association with return-to-work: was not investigated</td><td></td></tr>
<tr><td>2030
Chew,
2020,
Singapore

High risk</td><td>Study design: Longitudinal
Time of Study:
Baseline: 5 March - 10 April 2020
Follow up: 8 June - 2 July 2020
Follow-up mean, range: 3-4 months</td><td>Job description: 2 groups of medical residents in training:
Medical: Medical specialties, Radiology,Family medicine, Psychiatry;
Surgical: Surgery, Anaesthesia, Emergency medicine
Inclusion criteria: none
invited: n=557 (all residents)
baseline: n=274
follow -up: n=221
Age at baseline: 30.6 (SD ±2.7) years
% of females: 51.5%
Response: 49.2%
Loss-to-follow-up: 19.3%</td><td>During COVID-19 pandemic (not specified)</td><td>Healthcare Workers Stigma Scale (HWSS), 12 items, 4-point Likert scale (1 =strongly disagree, 4= strongly agree)

4 subscales:
1.Self-stigma (Personalized stigma)
2.Disclosure concerns
3.Concerns about public attitudes
4. Negative self-image

Form of stigmatization:
<u>Self-stigma (internalized):</u> negative self-image (feeling inferior to others due to occupation)
<u>Self-stigma (anticipated):</u> own perceived consequences, fear of rejection, disclosure concerns (control the information or keeping occupation a secret), concerns about public attitudes (what most people think of HCWs)</td><td>Prevalence of stigmatization:

<u>Self-stigma (internalized):</u>
Table 1: Demographic and clinical variables of <u>all residents</u> (baseline vs. follow-up)<table><tr><th></th><th>Baseline (mean SD)</th><th>Follow-up (mean SD)</th><th>t-test t</th></tr><tr><td>HWSS Disclosure Concerns subscale</td><td>6.3 (2.29)</td><td>5.8 (2.37)</td><td>2.15</td></tr><tr><td>HWSS Concerns about Public Attitudes subscale</td><td>6.6 (2.28)</td><td>6.0 (2.26)</td><td>3.08</td></tr><tr><td>HWSS Negative Self-image subscale</td><td>4.5 (1.90)</td><td>4.2 (1.71)</td><td>1.72</td></tr></table>
<u>Self-stigma (anticipated):</u>
Table 1: Demographic and clinical variables of <u>all residents</u> (baseline vs. follow-up)<table><tr><th></th><th>Baseline (mean SD)</th><th>Follow-up (mean SD)</th><th>t-test t</th></tr><tr><td>HWSS Personalized Stigma subscale</td><td>5.1 (1.7)</td><td>4.8 (1.7)</td><td>1.68</td></tr></table>
<u>Self-stigma (internalized and anticipated):</u><table><tr><th></th><th>Baseline (mean SD)</th><th>Follow-up (mean SD)</th><th>t-test t</th></tr></table></td><td>+ethical approval by the National Healthcare Group's Institutional Review Board, Singapore (NHG DSRB Ref: 2020/00220).
+ recruitment procedure
+ outcome source and validation: use of validated questionnaires
+ chronology
+ funding stated (none)
+ no conflict of interest
+ all residents invited

- confounding: no adjustment of age
- analysis method</td></tr>
</table>

<table>
<tr><th>General</th><th>Study</th><th>Population</th><th>Exposure</th><th>Outcome</th><th>Results</th><th>Comments</th></tr>
<tr><td></td><td></td><td></td><td></td><td></td><td>
<table>
<tr><td>HWSS total score</td><td>22.5 (6.8)</td><td>20.8 (6.9)</td><td>2.62</td></tr>
</table>

Table 3. Risk factors for HWSS amongst residents in training from national healthcare group residencies at the 2nd time point (linear regression).
<table>
<tr><td>Variable</td><td>B</td><td>ß</td><td>95% CI</td></tr>
<tr><td>Females (vs. males)</td><td>0.500</td><td>0.036</td><td>-1.153- 2.153</td></tr>
<tr><td>Married (vs. single)</td><td>- 1.504</td><td>- 0.109</td><td>- 3.180- 0.172</td></tr>
<tr><td>Living with others (vs. alone)</td><td>1.725</td><td>0.070</td><td>- 1.240- 4.690</td></tr>
</table>

Conditions increasing work-related stigmatization:
Table 3. Risk factors for HWSS amongst residents in training from national healthcare group residencies at the 2nd time point (linear regression).
<table>
<tr><td>Variable</td><td>B</td><td>ß</td><td>95% CI</td></tr>
<tr><td>Seniors (vs. juniors)</td><td>1.016</td><td>0.071</td><td>- 0.676-2.708</td></tr>
<tr><td>Exposed to patients with respiratory illness</td><td>0.451</td><td>0.028</td><td>- 1.544- 2.446</td></tr>
<tr><td>Deployed to National Center for Infectious Disesases</td><td>1.443</td><td>0.105</td><td>- 0.287- 3.174</td></tr>
</table>

Measures to prevent/ deal with stigmatization at work:
Tool used: was not investigated

Unfavourable health consequences associated with stigmatization: Perceived, and traumatic stress. Results are shown in Tab. 2.

Stigmatization studied in association with return-to-work: was not investigated
</td><td></td></tr>
<tr><td>775
Dang,
2020,</td><td>Study design:</td><td>Job description:
HCWs: n=120,
Professional</td><td>Social isolation:
Data</td><td>Disclosure and discrimination related to COVID-19 work: self-</td><td>Prevalence of stigmatization:

<u>Self-stigma (perceived):</u></td><td>+ ethic approval from Review Committee of the Institute for</td></tr>
</table>

<table>
<tr><th>General</th><th>Study</th><th>Population</th><th>Exposure</th><th>Outcome</th><th>Results</th><th>Comments</th></tr>
<tr>
<td>Vietnam

High risk</td>
<td>Cross-sectional
Time of Study: 7 - 14 April 2020
Follow-up mean, range: n.a.</td>
<td>educators: n=146, White collar workers: n=152, Students: n =147, Others: n=97
Inclusion criteria: none
# invited: n.a. snowball technique
# baseline: n=1423 participated
n=662 analysed
# follow-up: n.a.
Age at baseline: Mean: Males: 36.7 (SD 10.7) years
Females: 33.5 (SD ± 10.5) years
% of females: 69.3%
Response: n.a. (convenience sample)
Loss-to-follow-up: n.a.</td>
<td>collection one week after social distancing and lockdown have been ordered by government</td>
<td>developed, non-validated: 4 items from 0 (strongly disagree) to 4 (strongly agree)

Form of stigmatization:
<u>Self-stigma (perceived): social exclusion</u>
Being alienated because employment-related to COVID-19

<u>Self–stigma (anticipated):</u> Afraid of sharing with family about risks of exposure to COVID- 19 at work;
Avoid sharing occupational information

<u>Associative stigma: social exclusion</u>
Relatives being alienated because employment related to COVID-19</td>
<td>Being alienated because employment-related to COVID-19:
Number of participants with maximum score: n = 7
Mean: males: 2.3 (SD 0.9), females: 2.3 (SD 1.0)

<u>Self-stigma (anticipated):</u>
Afraid of sharing with family about risks of exposure to COVID-19 at work:
Number of participants with maximum score: n = 15
Mean: males: 2.3 (SD 1.1), females: 2.0 (SD 1.0)

Avoid sharing occupational information:
Number of participants with maximum score: n = 4
Mean: males: 2.1 (SD 0.9), females: 2.0 (SD 0.9)

<u>Associative stigma:</u>
Number of participants with maximum score: n = 7
Mean: males: 2.1 (SD 1.0), females: 2.1 (SD 0.9)

Table 4: Factors associated with COVID-19
<table>
<tr><td></td><td colspan="2">Disclosure and discrimination related to COVID-19 work exposure</td></tr>
<tr><td></td><td>Coef.</td><td>95% CI</td></tr>
<tr><td>Gender (Female vs male)</td><td>-0.14</td><td>-0.28; 0.00</td></tr>
<tr><td>Region (Central vs. Northern)</td><td>-0.28</td><td>-0.49; -0.06</td></tr>
<tr><td colspan="3">Occupation (vs HCW)</td></tr>
<tr><td>Professional educators</td><td>-0.28</td><td>-0.44; -0.11</td></tr>
<tr><td>White collar workers</td><td>-</td><td>-</td></tr>
<tr><td>Students</td><td>0.30</td><td>-0.06; 0.66</td></tr>
<tr><td colspan="3">Occupational status (vs salaried employees)</td></tr>
<tr><td>Self-employed/ unemployed/ retired</td><td>-0.39</td><td>-0.73; -0.04</td></tr>
<tr><td>Others</td><td>ref</td><td></td></tr>
</table>

Conditions increasing work-related stigmatization: was not investigated</td>
<td>Preventive Medicine and Public Health, Hanoi Medical University
+ no conflict of interest
+ confounding

- recruitment procedure: convenience sample
- outcome: self-developed questionnaire

Funding by a foundation of a joint stock company</td>
</tr>
</table>

<table>
<tr><th>General</th><th>Study</th><th>Population</th><th>Exposure</th><th>Outcome</th><th>Results</th><th>Comments</th></tr>
<tr><td></td><td></td><td></td><td></td><td></td><td>Measures to prevent/ deal with stigmatization at work: was not investigated

Unfavourable health consequences associated with stigmatization: was not investigated

Stigmatization studied in association with return-to-work: was not investigated</td><td></td></tr>
<tr><td>883
Do Duy, 2020, Vietnam

High risk</td><td>Study design: Cross-sectional
Time of Study: 26 - 29 April 2020
Follow-up mean, range: n.a.</td><td>Job description:
Clinicians: n=7
Nurses: n=45
Others: n=9
Inclusion criteria: Healthcare workers after quarantine
invited: n.r.
baseline: 61
follow-up: n.a.
Age at baseline: median 32 years (range 29-36)
% of females: 82%
Response: n.r.
Loss-to-follow-up: n.a.</td><td>Lock-down of workplace because of COVID-19 outbreak-> all HCWs required quarantining for 23 days. Data collection after quarantine</td><td>Stigma scale: self-developed instrument: 12 questions (4-point Likert scale), sum up score 0-36

Items from group discussion with HCWs and investigators, adaption of Berger's HIV Stigma Scale for terms and phrases (validity checked with COSMIN)

Form of stigmatization:
<u>Self-stigma (anticipated):</u> Feel unsafe to be a health worker, Feel blamed by relatives or friends, Try to hide being quarantined, Try to avoid going out

<u>Self-stigma (internalized):</u> Feel guilty because of being isolated, Feel ashamed of being isolated

<u>Self-stigma (perceived): discrimination</u> People talk behind back, People</td><td>Prevalence of stigmatization:
Median 11 (IR 6-15, min: 0, max: 24)

<u>Self-stigma (anticipated):</u>
Table 1: Factor analysis results of Stigma Scale
<table><tr><td>Item</td><td>% agreed</td></tr><tr><td>Feel unsafe to be a health worker</td><td>18.0</td></tr><tr><td>Feel blamed by relatives or friends</td><td>9.8</td></tr><tr><td>Try to hide being quarantined</td><td>6.6</td></tr><tr><td>Try to avoid going out</td><td>65.6</td></tr></table>
<u>Self-stigma (internalized):</u>
Table 1: Factor analysis results of Stigma Scale
<table><tr><td>Item</td><td>% agreed</td></tr><tr><td>Feel guilty because of being isolated</td><td>34.4</td></tr><tr><td>Feel ashamed of being isolated</td><td>3.3</td></tr></table>
<u>Self-stigma (perceived):</u>
Tab. 1: Factor analysis results of Stigma Scale:
<table><tr><td>Item</td><td>% agreed</td></tr><tr><td>People talk behind back</td><td>39.3</td></tr><tr><td>People avoid touching and direct contact</td><td>34.4</td></tr><tr><td>People feel uncomfortable when around</td><td>31.2</td></tr><tr><td>Experienced discrimination actions</td><td>6.6</td></tr></table>

Conditions increasing work-related stigmatization: was not investigated

Measures to prevent/ deal with stigmatization at work: was not investigated</td><td>+ study approved by director board of hospital
+ informed consent
+ all employees invited
+ no conflict of interest
+ outcome: self-developed instrument validated with COSMIN Risk of Bias Checklist

- recruitment procedure (# invited not reported)
- analysis method: statistical methods not appropriate -> only descriptive summary and correlations
- confounders not considered
- not reported which sub-sample was used for analyses
- funding no reported</td></tr>
</table>

<table>
<tr><th>General</th><th>Study</th><th>Population</th><th>Exposure</th><th>Outcome</th><th>Results</th><th>Comments</th></tr>
<tr><td></td><td></td><td></td><td></td><td>avoid touching and direct contact, People feel uncomfortable when around, Experienced discrimination actions</td><td>Unfavourable health consequences associated with stigmatization: Depression, anxiety, and stress. Results are shown in Tab. 2.

Stigmatization studied in association with return-to-work: yes -> data collection after quarantine of all workers in a hospital that was locked-down because of SARS-CoV2 outbreak</td><td></td></tr>
<tr><td>7271
Dye,
USA,
2020

High risk</td><td>Study design: Cross-sectional study with qualitative part
Time of Study: 6 April - 29 May
Follow-up mean, range: n.a.</td><td>Job description: n.r. (-> all)
Inclusion criteria: none
invited: convenience sample -> recruitment via mTURK (access to a multilingual digital workforce) and Facebook, Instragram, facebook Audience Network
baseline: n = 7411 from 173 countries
follow-up: n.a.
Age at baseline: n.r.
% of females: n.r.
Response: n.a. (convenience sample)
Loss-to-follow-up: n.a.</td><td>During COVID-19 pandemic -> Working in health care setting around the world</td><td>1 item, derived from the KFF Corona-virus Poll 26 question: Do you or anyone in your household work in a healthcare delivery setting, such as a doctor's office, clinic, hospital, nursing home or dentist's office?

Have you or a family member been harassed, bullied or hurt because of coronavirus? (yes, no/don't know) –> If yes, participants were asked to explain (open-end question)

Form of stigmatization:
<u>Self-stigma (perceived):</u> <u>aggressive behaviour/mobbing</u>

<u>Associative stigma:</u> <u>aggressive behaviour/mobbing</u></td><td>Prevalence of stigmatization:

<u>Self-stigma (perceived) and associative stigma:</u>

Tab.1: Association of COVID-19-related variables with working in HC-setting and COVID-19 related harassment and bullying
<table>
<tr><th></th><th colspan="2">Harassed, bullied or hurt because of coronavirus</th><th>M1 (crude)</th><th>M2</th></tr>
<tr><td></td><td>Yes
n (%)</td><td>No
n (%)</td><td>OR (95% CI)</td><td>OR (95% CI)</td></tr>
<tr><td>I work in a healthcare setting</td><td>151 (18.1)</td><td>685 (81.9)</td><td rowspan="2">2.9 (2.3 to 3.5)</td><td rowspan="2">1.5 (1.2 to 1.9)</td></tr>
<tr><td>I don't work in a healthcare setting</td><td>444 (7.1)</td><td>5771 (92.9)</td></tr>
</table>
M2 adjusted for: could not work from home, have/have had COVID-19, have not closely followed social-physical distancing, have family/neighbour with COVID-19, have friend/family/neighbour who died from COVID-19, have at least one chronic disease, have child care responsibilities, have elder care responsibilities, reside in Africa/Asia/Northern America/Oceania

<u>Qualitative part:</u>
See table for qualitative studies

Conditions increasing work-related stigmatization: was not investigated</td><td>+ ethic approval from University of Rochester's Research Subjects Review Board
+ no conflict of interest
+ Funding source
+ confounding
+ analysis method

- recruitment procedure: convenience sample
- outcome: only one question for harassment/bullied, not validated</td></tr>
</table>

<table>
<tr><th>General</th><th>Study</th><th>Population</th><th>Exposure</th><th>Outcome</th><th>Results</th><th>Comments</th></tr>
<tr><td></td><td></td><td></td><td></td><td></td><td>Measures to prevent/ deal with stigmatization at work: was not investigated

Unfavourable health consequences associated with stigmatization: was not investigated

Stigmatization studied in association with return-to-work: was not investigated</td><td></td></tr>
<tr><td>859
Elhadi, 2020, Libya

High risk</td><td>Study design: Cross-sectional
Time of Study: 18 - 28 April 2020
Follow-up mean, range: n.a.</td><td>Job description: HCWs (doctors and nurses) from 15 hospitals working during the outbreak period (March and April 2020)
Inclusion criteria: exclusion of participants with a history of mental illness and exclusion of those who did not work during the outbreak period (March and April 2020)
invited: n.a. snowball technique
baseline: n=745
follow-up: n.a.
Age at baseline: mean 33.3 years (SD± 7.4)
% of females: 51.9%
Response: n.a. (convenience sample)Loss-to-follow-up: n.a.</td><td>During COVID-19 pandemic (not specified) and civil war in Libya</td><td>Stigmatization: single item “feeling stigmatized” (yes/no)

Form of stigmatization: <u>Self-stigma (perceived)</u></td><td>Prevalence of stigmatization:

<u>Self-stigma (perceived):</u>

Tab. 1: Feeling stigmatized due to COVID-19 (N=745)<table><tr><th>Healthcare workers</th><th>n</th><th>%</th></tr><tr><td>Feeling stigmatized</td><td>231</td><td>31</td></tr></table>Conditions increasing work-related stigmatization: was not investigated

Measures to prevent/ deal with stigmatization at work: was not investigated

Unfavourable health consequences associated with stigmatization: Depressive symptoms, anxiety symptoms
Results are shown in Tab. 2.

Stigmatization studied in association with return-to-work: was not investigated</td><td>+ informed consent
+ approval by the Bioethics Committee of the Biotechnology Research Center
+ no conflict of interest
+ research received no funding
+ measurement of depression and anxiety with a validated instrument

- recruitment procedure: convenience sample
- outcome: measurement of stigmatization with a single question
- analysis method:
- investigation of the association between stigmatization and depressive and anxiety symptoms only with Pearson's chi-square test
- no consideration of any confounders in the analysis of the association between</td></tr>
</table>

<table>
<tr><th>General</th><th>Study</th><th>Population</th><th>Exposure</th><th>Outcome</th><th>Results</th><th>Comments</th></tr>
<tr><td></td><td></td><td></td><td></td><td></td><td></td><td>stigmatization and depressive and anxiety symptoms
- no investigation of a temporal relationship possible due to the cross-sectional design</td></tr>
<tr><td>7273
Elhadi, 2020, Libya

High risk</td><td>Study design: Cross-sectional
Time of Study: 18 April - 2 May 2020
Follow-up mean, range: n.a.</td><td>Job description: HCWs
Inclusion criteria: working in late March and April, and in either surgery, internal medicine, intensive care, or emergency departments
Exclusion: participants with history of mental illness, having patients with mental illnesses or severe chronic diseases such as advanced diabetes, hypertension, and tuberculosis
invited: n=600
baseline: n=532
follow -up: n.a.
Age at baseline: mean: 33.1 years (SD ±7.3)
% of females: 44.7%
Response: 88.7%
Loss-to-follow-up: n.a.</td><td>During COVID-19 pandemic (not specified) and civil war in Libya</td><td>Stigmatization: single item “feeling stigmatized” (yes/no)

Form of stigmatization:
<u>Self-stigma (perceived)</u></td><td>Prevalence of stigmatization:

<u>Self-stigma (perceived)</u>

Tab. 1: Baseline characteristics of study participants (n = 532)
<table>
<tr><th>Variable</th><th>Total (%) n=532</th><th>Women (%) n=238</th><th>Men (%) n=294</th><th>Phi</th><th>p-value</th></tr>
<tr><td colspan="6">Stigmatization due to COVID-19</td></tr>
<tr><td>Yes</td><td>169 (31.8)</td><td>86 (36.1)</td><td>83 (28.2)</td><td rowspan="2">0.08</td><td rowspan="2">0.052</td></tr>
<tr><td>No</td><td>363 (68.2)</td><td>152 (63.9)</td><td>211 (71.8)</td></tr>
</table>

Conditions increasing work-related stigmatization: was not investigated

Measures to prevent/ deal with stigmatization at work: was not investigated

Unfavourable health consequences associated with stigmatization: Emotional exhaustion, depersonalization
Results are shown in Tab. 2.

Stigmatization studied in association with return-to-work: was not investigated</td><td>+ outcome: usage of a validated measuring tool for burnout
+ re-validation with study sample
+ ethics approval by the Bioethics Committee at the Biotechnology Research Center
+ informed consent
+ recruitment procedure

- outcome: measurement of stigmatization with a single item question
- analysis method: investigation between stigmatization and burnout only with Spearman’s rank-order correlation test
- no consideration of confounders
- cross-sectional study design (no causation between variables possible)
- fear of stigmatization may</td></tr>
</table>

General	Study	Population	Exposure	Outcome	Results	Comments
						have resulted in response bias<br>- funding not clear<br>- no information on conflict of interest
7279<br>Greene, 2020,<br>UK<br><br>High risk	**Study design:** cross-sectional<br>**Time of Study:** 27 May - 23 July 2020<br>**Follow-up mean, range:** n.a.	**Job description:** Frontline health and social care workers working in a variety of healthcare roles in UK hospitals, nursing or care homes, and community settings<br>Allied healthcare: n=136,<br>Carers: n=105<br>Clinical support role: n=204<br>Doctors: n=46<br>Non-clinical staff: n=62<br>Nurses or midwifes: n=504<br>Other roles: n=133<br>**Inclusion criteria:** none<br>**# invited:** n.a. snowball technique<br>**# baseline:** n=1194<br>**# follow-up:** n.a.<br>**Age at baseline:** mean 41.5 years (SD ±11.5)<br>**% of females:** 92.4%<br>**Response:** n.a. (convenience sample)	During COVID-19 pandemic (post-peak phase of the initial COVID-19 wave in the UK)	Feeling stigmatized for being an NHS worker: single Likert scale ranging from 0 (“not at all”) to 4 (“extremely”)<br><br>**Form of stigmatization:** <u>Self-stigma (perceived)</u>	**Prevalence of stigmatization:**<br><br><u>Self-stigma (perceived)</u><br><br>Feeling moderately to extremely stigmatized: n=435 (36.5%)<br><br>**Conditions increasing work-related stigmatization:** was not investigated<br><br>**Measures to prevent/ deal with stigmatization at work:** was not investigated<br><br>**Unfavourable health consequences associated with stigmatization:** Distress, PTDS, anxiety and depression. Results are shown in Tab. 2.<br><br>**Stigmatization studied in association with return-to-work:** was not investigated	+ approval by the UCL Ethics Committee<br>+ measurement of PTDS, depression, and anxiety with validated instruments<br>+ using logistic regression analysis in order to investigate the association between stigmatization and mental health outcomes<br><br>- recruitment procedure: convenience sample<br>- conflict of interest not reported<br>- funding not reported<br>- outcome: measurement of stigmatization with a single question<br>- no consideration of any confounders in the analysis of the association between stigmatization and mental health outcomes<br>- analysis method<br>- no investigation of a temporal relationship

General	Study	Population	Exposure	Outcome	Results	Comments
		**Loss-to-follow-up:** n.a.				possible due to the cross-sectional design
759<br>Juan,<br>2020,<br>China<br><br>High risk	**Study design:** Cross-sectional<br>**Time of Study:** 1 - 14 February 2020<br>**Follow-up mean, range:** n.a.	**Job description:** hospital staff from five national COVID-19 designated hospitals (working in isolation ward: n=96, general ward: n=360)<br>Doctors: n=195<br>Nurses: n=261<br>**Inclusion criteria:** exclusion of those participants who had experienced insomnia or psychiatric disorders prior to COVID-19, and those who had organic diseases<br>**# invited:** n=500<br>**# baseline:** n=456<br>**# follow-up:** n.a.<br>**Age at baseline:** mean 30.7 years (SD ±7.5) (range: 17-64 years)<br>**% of females:** 70.6%<br>**Response:** 91.2%<br>**Loss-to-follow-up:** n.a.	During COVID-19: study period corresponds with the highest point of the COVID-19 epidemic in China	Stigmatization and rejection in neighbourhood because of hospital work: 1 item question (yes, no)<br><br>**Form of stigmatization:** <u>Self-stigma (perceived): discrimination</u>	**Prevalence of stigmatization:** was not investigated<br><br>**Conditions increasing work-related stigmatization:** was not investigated<br><br>**Measures to prevent/ deal with stigmatization at work:** was not investigated<br><br>**Unfavourable health consequences associated with stigmatization:** Psychological reactions, obsessive-compulsive symptoms, somatization symptoms, anxiety disorder, and depression. Results are shown in Tab. 2.<br><br>**Stigmatization studied in association with return-to-work:** was not investigated	+ recruitment procedure: random selection of participants<br>+ high response rate<br>+ approval by the ethics committees of the third affiliated hospital<br>+ measurement of psychological impact due to stigmatization with validated instruments<br>+ analysis method: using multiple logistic regression analysis in order to investigate the association between stigmatization and rejection in neighbourhood and mental health outcomes<br>+ no conflict of interest<br>+ funding from non-profit organizations<br><br>- outcome: measurement of stigmatization with a single question<br>- no consideration of any confounders in the analysis of the association between

General	Study	Population	Exposure	Outcome	Results	Comments
						stigmatization and rejection in neighbourhood and mental health outcomes
749 Khanal, 2020, Nepal<br><br>High risk	**Study design:** Cross-sectional<br>**Time of Study:** 26 April - 12 May 2020<br>**Follow-up mean, range:** n.a.	**Job description:**<br>Nurses: n=167<br>Doctors: n=161<br>Paramedics: n=81<br>Laboratory staff: n=19<br>Pharmacists: n=15<br>Public health professional: n=32<br>**Inclusion criteria:** >18 years, currently working in Nepal in COVID-19 management.<br>**# invited:** n.r.<br>**# baseline:** n=501 responses received<br>n=475 analysed<br>**# follow -up:** n.a.<br>**Age at baseline:** mean 28.2 (SD ±5.8) years<br>**% of females:** 52.8%<br>**Response:** n.r.<br>**Loss-to-follow-up:** n.a.	Nationwide lockdown imposed from March 24, which continued for nearly 10 weeks.	1 item: Stigma faced due to COVID-19 (yes, no, don't want to answer)<br><br>**Form of stigmatization**: Self-stigma (perceived): discrimination, aggressive behaviour/mobbing	**Prevalence of stigmatization:**<br><br>Self-stigma (perceived):<br>Experience of stigma due to occupation from community members<br>• Yes: n=255 (53.7%)<br>• No: n=199 (41.9%)<br>• Do not want to answer: n=21 (4.4%)<br><br>Type of major stigma experienced (n=255)<br>• Stigmatized because of profession: n=127 (49.8%)<br>• Accused of being a carrier of disease: n=102 (40.0%)<br>• Threatened: n=15 (5.9%)<br>• Asked to leave rented place: n=11 (4.3%)<br><br>**Conditions increasing work-related stigmatization:** was not investigated<br><br>**Measures to prevent/ deal with stigmatization at work:** was not investigated<br><br>**Unfavourable health consequences associated with stigmatization:** Anxiety, depression, and insomnia. Results are shown in Tab. 2.<br><br>**Stigmatization studied in association with return-to-work:** was not investigated	+ethical approval by the Nepal Health Research Council (Reference number: 2192, 315/2020)<br>+no conflict of interests<br>+ measurement of psychological impact due to stigmatization with validated instruments<br><br>- no funding reported<br>- recruitment procedure: # invited not reported<br>- outcome: stigmatization only assessed with 1 item
7269 Khanal, Nepal 2020					**Unfavourable health consequences associated with stigmatization:** Fear of COVID-19. Results are shown in Tab. 2.	
7285 Mohindra, 2020,	**Study design:**	**Job description:**<br>Doctors: n=167<br>Nurses: n=178	During the nationwide lockdown	Questionnaire self-developed and based on:	**Prevalence:**<br><br>Self-stigma (perceived and internalized):	+ ethical approval<br>+ questionnaire self-developed based on

General	Study	Population	Exposure	Outcome	Results	Comments
India<br><br>High risk	Cross-sectional<br>**Time of Study:**<br>15 - 26 April 2020<br>**Follow-up mean, range:**<br>n.a.	Hospital attendants: n=109<br>Sanitation attendants: n=109<br>Other: n=12<br>**Inclusion criteria:** any person working in the hospital<br>**# invited:** n.a. convenience sample (invitation via WhatsApp work groups)<br>**# baseline:** n=574<br>**# follow -up:** n.a.<br>**Age at baseline:** 30.2 (SD ±6.5) years<br>**% females:** 63.8%<br>**Response:** n.a..(convenience sample)<br>**Loss-to-follow-up:** n.a.	(started 25 March 2020)	- the domains of experienced changes at workplace/ hospital, neighbourhood/ community, self/ family and emotional state (McMahon et al. 2016)<br>- 19 items (open-end and multiple choice questions)<br>- piloted n=10<br><br>**Form of stigmatization:**<br>Self-stigma (perceived): stereotypes, discrimination, social exclusion, aggressive behaviour/mobbing<br><br>Self-stigma (internalized)	Percentage of frequency of items of perceived change in different settings during the COVID-19 pandemic:<br><br>*Hospital (Workplace)*<br>My colleagues and peers: n=294<br>• Try to avoid me (52.7%)<br>• Do not trust me and worry that I might infect them (50.7%)<br>• Do not sit and eat with me (31.0%)<br><br>*Neighbourhood /Community*<br>My neighbours: n=312<br>• Try to avoid me (49.7%)<br>• Do not trust me and worry that I might infect them (40.7%)<br>• Do not sit and eat with me (28.5%)<br>• Have asked me or my family members to leave and not enter the neighbourhood (13.8%)<br>• Don't trust each other anymore (11.5%)<br>• Have verbally abused me or physically assaulted me or my family members (7.1%)<br><br>*Family and home*<br>My family members: n=137<br>• Do not trust me and worry that I might infect them (32.8%)<br>• Try to avoid me (32.8%)<br>• Do not sit and eat with me (25.5%)<br>• Have asked me to leave or not enter my home (20.4%)<br>• Don't trust each other anymore (13.5%)<br>• Have verbally abused me or physically assaulted me (5.1%)<br>• My intimate partner tries to avoid me (59.1%)<br><br>At times I have felt: n=570<br>• Guilty about possibly exposing family, community and peers to infection (20.9%)<br>• Lonely and ostracized (16.1%)<br><br>**Conditions increasing work-related stigmatization:** was not investigated<br><br>**Measures to prevent/ deal with stigmatization at work:** was not investigated	other questionnaires, piloted<br><br>- funding not stated<br>- conflict of interests not stated<br>- recruitment procedure: convenience sample (the link to questionnaire circulated among groups of employees via WhatsApp)<br>- confounding<br>- analysis method

General	Study	Population	Exposure	Outcome	Results	Comments
					**Unfavourable health consequences associated with stigmatization:** was not investigated<br><br>**Stigmatization studied in association with return-to-work:** was not investigated	
948 Monterrossa-Castro, 2020, Colombia<br><br>High risk	**Study Design:** Cross-sectional **Time of study:** 1 - 5 April 2020 **Follow-up mean, range:** n.a.	**Job description:** General practitioners **Inclusion criteria:** General practitioners who treated patients of any sex, age, or type of pathology or health condition **# invited:** n.r. **# baseline:** N=531 analysed **# follow -up:** n.a. **Age at baseline:** mean 33.0 (SD ±9.3) years **% of females:** 59.5% **Response:** n.r. **Loss-to-follow-up:** n.a.	Responses to "the 24–30 March period, when the country was in a health emergency, in the initial phase of containment".	Social discrimination for working as a general practitioner (questions not described)<br><br>**Forms of stigmatization:** <u>Self-Stigma (perceived): discrimination</u>	**Prevalence of stigmatization:**<br><u>Self-stigma (perceived)</u><br>Social discrimination for working as a general practitioner: n = 207 (38.9%)<br><br>**Conditions increasing work-related stigmatization:** was not investigated<br><br>**Measures to prevent/ deal with stigmatization at work:** was not investigated<br><br>**Unfavourable health consequences associated with stigmatization:** anxiety. Results are shown in Tab. 2.<br><br>**Stigmatization studied in association with return-to-work:** was not investigated	+ ethical approval by the University of Cartagena (Act 064–2019 and Resolution 01 430–2019)<br>+ Conflict of interest stated<br>+ Funding stated (none)<br>+ outcome: measurement of psychological impact due to stigmatization with validated instruments<br><br>- no description of social discrimination for working as a general practitioner<br>- recruitment procedure; no description of recruitment, # invited not reported
7261 Ramaci, Italy, 2020<br><br>High risk	**Study design:** Cross-sectional **Time of Study:** 17 - 26 March	**Job description:** Nurses: n=67 Doctors: n=206 **Inclusion criteria:** none **# invited**: n.a. (convenience sample) **# baseline:** n=273	During COVID-19 pandemic: national lockdown (17 March - 2 April)	"Stigma discrimination" "Stigma fear": no description of definition included; adapted from See et al. 2011 (answer options: 0 = strongly disagree, 1 = disagree, 2 = agree, 3 = strongly agree)	**Prevalence of stigmatization:**<br><br><u>Self-stigma (perceived):</u><br>Males: 1.57 (SD 0.70)<br>Females:1.45 (SD 0.66)<br><br><u>Self-stigma (anticipated):</u><br>Males: 3.07 (SD 0.71)<br>Females: 3.10 (SD 0.97)	+ no conflict of interest<br>+ Funding stated (none)<br>+ outcome: measurement of psychological impact due to stigmatization

<table>
<tr><th>General</th><th>Study</th><th>Population</th><th>Exposure</th><th>Outcome</th><th>Results</th><th>Comments</th></tr>
<tr>
<td></td>
<td>Follow-up mean, range: n.a.</td>
<td># follow-up: n.a.
Age at baseline: 46.7 years (SD ±8.4)
% of females: 50.2%
Response: n.a. (convenience sample)
Loss-to-follow-up: n.a.</td>
<td></td>
<td>Form of stigmatization:
Self-stigma (perceived): discrimination

Self-stigma (anticipated)</td>
<td>Conditions increasing work-related stigmatization: was not investigated

Measures to prevent/ deal with stigmatization at work: was not investigated

Unfavourable health consequences associated with stigmatization: compassion fatigue, compassion satisfaction, and risk of burnout. Results are shown in Tab. 2.

Stigmatization studied in association with return-to-work: was not investigated</td>
<td>with validated instruments

- recruitment procedure: convenience sample
- outcome: no description of how stigmatization was measured
- confounding: age not considered</td>
</tr>
<tr>
<td>7130 Said, Egypt, 2021

High risk</td>
<td>Study design: Cross-sectional (comparative study)
Time of Study: 10 - 24 April 2020
Follow-up mean, range: n.a.</td>
<td>Job description:
A) Nurses from triage hospital (ZFH):
B) Nurses from a hospital with no triage or isolation form (ZGH)
Inclusion criteria: All on-job nurses (excluding: pregnant women and nurses on extended leave > 6 weeks)
invited: convenience sample: invitation through online work group on “WhatsApp” and “Facebook”; online questionnaire (Google Form App); stopped when sample size was achieved</td>
<td>During COVID-19 pandemic (not specified)</td>
<td>Stigma: 2 items (derived from US National Centre for Posttraumatic Stress Disorder 2020 and “MERS-CoV staff questionnaire” Khalid et al. 2016

1.”Others’ fear of contact with those treating patients with COVID-19”
2.”Developing self-stigma about voicing needs and fears (score range 0-8)”

Form of stigmatization:
Self-stigma (perceived): discrimination
“Others’ fear of contact with those treating patients with COVID-19)”,
Self-stigma (internalized):
“Developing self-stigma</td>
<td>Prevalence of stigmatization:

Self-stigma (perceived and anticipated)

ZFH group: n=190 (90.5%)
ZGH group: n=42 (20%)

Tab.S1: Subscale of specific COVID-19 associated stressors
<table>
<tr><th></th><th>ZFH</th><th>ZGH group</th><th>Difference between groups</th></tr>
<tr><td>Stigma (both)</td><td>7.9 (SD 1.4)</td><td>6.6 (SD 1.1)</td><td>p < 0.001</td></tr>
<tr><td>Self-stigma (perceived)</td><td>4.1 (SD 0.8)</td><td>3.4 (SD 0.7)</td><td>p < 0.001</td></tr>
<tr><td>Self-stigma (internalized)</td><td>3.8 (SD 0.8)</td><td>3.2 (SD 0.8)</td><td>p < 0.001</td></tr>
</table>

Conditions increasing work-related stigmatization: was not investigated

Measures to prevent/ deal with stigmatization at work: was not investigated</td>
<td>+ ethic approval from university ethics committee
+ no conflict of interest
+ sample size calculation
+ pilot study for testing (n=40) questionnaire und feasibility of study (results not included)

- recruitment procedure: convenience sample
- outcome measured with 2 items (not validated)
- confounding: effect of age not considered
- analysis method: only basic statistical evaluation</td>
</tr>
</table>

<table>
<tr><th>General</th><th>Study</th><th>Population</th><th>Exposure</th><th>Outcome</th><th>Results</th><th>Comments</th></tr>
<tr><td></td><td></td><td># baseline: n=420
ZFH n=210
ZGH n=210
follow-up: n.a.
Age at baseline:
ZFH: 24.2 years (SD ±5.2)
ZGH: 26.1 years (SD ±4.5)
% of females: 100%
Response: n.a. (convenience sample)
Loss-to-follow-up: n.a.</td><td></td><td>about voicing needs and fears”</td><td>Unfavourable health consequences associated with stigmatization: was not investigated

Stigmatization studied in association with return-to-work: was not investigated</td><td></td></tr>
<tr><td>832
Sharma 2020,
US

High risk</td><td>Study design: Cross-sectional
Time of Study: 23 April - 7 May 2020
Follow-up mean, range: n.a.</td><td>Job description: Healthcare professionals caring for COVID-19 patients (intensive care unit (ICU) providers)
Physicians: 25%
Nurses: 47%
Respiratory therapists: 17%
Advanced practice providers (nurse practitioners, physician assistants, and certified registered nurse anaesthetists): 11%
Inclusion criteria: caring for COVID-19 patients
invited: n.a. snowball technique</td><td>During COVID-19 pandemic (not specified)</td><td>Stigma from community: instrument not reported

Form of stigmatization:
<u>Self-stigma (perceived)</u></td><td>Prevalence of stigmatization:

<u>Self-stigma (perceived)</u>

Figure 2 and Table 2: Stigma from the community
<table><tr><th>Health care professional</th><th>%</th></tr><tr><td>Total sample</td><td>26</td></tr><tr><td>Doctor</td><td>11</td></tr><tr><td>Doctor in training</td><td>18</td></tr><tr><td>Advanced practice provider</td><td>19</td></tr><tr><td>Respiratory therapist</td><td>29</td></tr><tr><td>Nurse</td><td>33</td></tr></table>
Conditions increasing work-related stigmatization: was not investigated

Measures to prevent/ deal with stigmatization at work: was not investigated

Unfavourable health consequences associated with stigmatization: Emotional distress/burnout. Results are shown in Tab. 2.

Stigmatization studied in association with return-to-work: was not investigated</td><td>+ study followed STROBE guidelines for the reporting of cross-sectional studies
+ ethical approval by the University of Washington Institutional Review Board
+ outcome: design of the questionnaire by a research team of physicians, nurses, respiratory therapists, and advanced practice providers
+ pilot-testing of the questionnaire by other healthcare professionals
+ no conflict of interest
+ funding stated (none)</td></tr>
</table>

<table>
<tr><th>General</th><th>Study</th><th>Population</th><th>Exposure</th><th>Outcome</th><th>Results</th><th>Comments</th></tr>
<tr><td></td><td></td><td># baseline: n = 1651
follow-up: n.a.
Age at baseline: n.r.
% of females: 74%
Response: n.a. (convenience sample)
Loss-to-follow-up: n.a.</td><td></td><td></td><td></td><td>- recruitment procedure: convenience sample
- outcome: instruments used to measure stigmatization and burnout not reported
- confounding factors were not reported
- analysis method</td></tr>
<tr><td>7262
Tan,
2020,
China

High risk</td><td>Study design: Cross-sectional
Time of Study: 24 - 25 February 2020
Follow-up mean, range: n.a.</td><td>Job description: Members of the workforce who returned to work: Workers, and technical staff: 81.1%
Executives, sales and marketing, management and others: 18.1%
Inclusion criteria: Members of the workforce, living in Chongqing, full-time employees, exclusion of health care workers, presence of severe psychiatric illnesses (e.g. schizophrenia, bipolar disorder, dementia), life-threatening medical conditions including severe stroke or life-threatening cancer</td><td>Returning to work after lockdown and quarantine in Chongqing, during the peak of the COVID-19 epidemic when strict infection control was in place</td><td>Experience of discrimination during COVID-19 epidemic: measured with a single-item (answer options: no, mild, moderate, serious, very serious discrimination)

Form of stigmatization:
<u>Self-stigma (perceived): discrimination</u></td><td>Prevalence of stigmatization:

<u>Self-stigma (perceived)</u>

Tab.2: Experience of discrimination during COVID-19 epidemic
<table>
<tr><th></th><th>All respondents (n=673)</th><th>Workers and technical staff (n=551)</th><th>Management and executive staff (n=122)</th><th>p</th></tr>
<tr><td>No discrimination</td><td>629 (93.5%)</td><td>511 (92.7%)</td><td>118 (96.7%)</td><td rowspan="5">0.435</td></tr>
<tr><td>Mild discrimination</td><td>24 (3.6%)</td><td>21 (3.8%)</td><td>3 (2.5%)</td></tr>
<tr><td>Moderate discrimination</td><td>10 (1.5%)</td><td>10 (1.8%)</td><td>0 (0.0%)</td></tr>
<tr><td>Serious discrimination</td><td>6 (0.9%)</td><td>5 (0.9%)</td><td>1 (0.8%)</td></tr>
<tr><td>Very serious discrimination</td><td>4 (0.6%)</td><td>4 (0.7%)</td><td>0 (0.0%)</td></tr>
</table>

Conditions increasing work-related stigmatization: was not investigated

Measures to prevent/ deal with stigmatization at work: was not investigated

Unfavourable health consequences associated with stigmatization: was not investigated</td><td>+ ethical approval by the Institutional Review Board of the China-Singapore (Chongqing) Demonstration Initiative on Strategic Connectivity Think Tank
+ no competing Interest

- only few information on the recruitment procedure (recruitment of participants through companies)
- exposure measurement with a single item-question
- confounders (for stigma analysis)
- analysis method (for stigma outcome)

Funding by public institutions and by a</td></tr>
</table>

<table>
<tr><th>General</th><th>Study</th><th>Population</th><th>Exposure</th><th>Outcome</th><th>Results</th><th>Comments</th></tr>
<tr><td></td><td></td><td>and suspected or confirmed cases of COVID-19 that prevented an employee from full-time employment
 # invited: n=1323
 # baseline: n=673
 # follow -up: n.a.
Age at baseline: mean: 30.8 years (SD ±7.4)
% of females: 25.6%
Response: 50.9%
Loss-to-follow-up: n.a.</td><td></td><td></td><td>**Stigmatization studied in association with return-to-work:** *yes*</td><td>foundation of a joint stock company</td></tr>
<tr><td>851
Taylor, 2020, USA, Canada

High risk</td><td>**Study design:** Cross-sectional
Time of Study: 6-19 May 2020
Follow-up mean, range: n.a</td><td>**Job description:** Non-HCWs from the United States and Canada :
92 % employed full- or part-time.
Inclusion criteria: none
 # invited: n.r.
 # baseline: N=3551
n=1716 from the USA
n=1835 from Canada
 # follow -up: n.a.
Age at baseline: mean 54 (SD ±15) years
% of females: 42%
Response: n.r.
Loss-to-follow-up:</td><td>During COVID-19 pandemic (not specified)</td><td>Attitudes from the general population towards HCWs

HCW Stigmatization Survey: self-developed, 8 items, 5-point scale (0=strongly disagree, 4=strongly agree), excellent reliability

Forms of stigmatization:
<u>Public Stigma: stereotypes, discrimination, social exclusion</u></td><td>**Prevalence of stigmatization:**

<u>Public Stigma:</u>

Tab. 2: Stigmatizing attitudes from (1) total respondents and from (2) people who clapped or cheered for HCWs (n=623)
<table>
<tr><th></th><th>**(1)** % agree or strongly agree (total)</th><th>**(2)** % agree or strongly agree (clapped)</th></tr>
<tr><td>Healthcare workers who work in hospitals are likely to have COVID-19</td><td>32</td><td>39</td></tr>
<tr><td>For the safety of the community, healthcare workers should not go out in public</td><td>25</td><td>28</td></tr>
<tr><td>Healthcare workers should have some restrictions on their freedom</td><td>26</td><td>29</td></tr>
<tr><td>Healthcare workers who treat people with COVID-19 should be isolated</td><td>36</td><td>39</td></tr>
</table></td><td>+ funding from: Canadian Institutes of Health Research (#439751) and the University of Regina
+ outcome: Validation of self-developed instrument
+ measurement of psychological impact due to stigmatization with validated instruments
+ declaration of competing interests (support by non-profit organizations and journals)

- no ethical approval (but written informed consent)
- recruitment and sampling technique</td></tr>
</table>

<table>
<tr><th>General</th><th>Study</th><th>Population</th><th>Exposure</th><th>Outcome</th><th>Results</th><th>Comments</th></tr>
<tr><td></td><td></td><td>n.a.</td><td></td><td></td><td><table>
<tr><td>I do not want to be around healthcare workers who treat COVID-19 patients</td><td>47</td><td>42</td></tr>
<tr><td>I do not want to be around someone who works in a healthcare setting</td><td>33</td><td>30</td></tr>
<tr><td>Healthcare workers who treat people with COVID-19 should be separated from their families</td><td>31</td><td>35</td></tr>
<tr><td>I would not be comfortable visiting a healthcare worker for medical reasons because I would be worried I might get COVID-19</td><td>34</td><td>38</td></tr>
</table>Applauding HCWs: “In the past seven days, I have clapped and cheered for healthcare workers”

Conditions increasing work-related stigmatization:
was not investigated

Measures to prevent/ deal with stigmatization at work:
was not investigated

Unfavourable health consequences associated with stigmatization: COVID-19 Stress Syndrome. Results are shown in Tab. 2.

Stigmatization studied in association with return-to-work: was not investigated</td><td>not reported; # invited not reported
- analysis method: only descriptive statistics applied
- confounders</td></tr>
<tr><td>865
Uvais,
India,
2020

High risk</td><td>Study design: Cross-sectional
Time of Study: 25 - 27 April
Follow-up mean, range: n.a.</td><td>Job description: Doctors working in hospitals; 72.4% part of the medical team directly involved in COVID-19 management; 29.2% history of COVID-19 positive patients, 31% history of quarantine
Speciality:</td><td>During COVID-19 pandemic (not specified)</td><td>Perceived stigma: stigma scale with 13 items (each scored on a 5-point Likert scale, total score 0 – 52; total score ≥26: high levels of perceived stigma)

Form of stigmatization:
<u>Self-stigma (perceived): discrimination, social exclusion</u></td><td>Prevalence of stigmatization:

<u>Self-stigma (perceived and anticipated)</u>

n=36 (62.1%) had a score ≥ 26 on the stigma scale
Mean: 28.3 (SD 8.8)
Association stigma & sex: ($\chi 2$ = 8.7)

Conditions increasing work-related stigmatization: was not investigated

Measures to prevent/ deal with stigmatization at work: was not investigated</td><td>+ no conflict of interest
+ funding stated (none)
+ outcome: measurement of psychological impact due to stigmatization with validated instruments

- no ethical approval</td></tr>
</table>

General	Study	Population	Exposure	Outcome	Results	Comments
		Critical care n=10 Internal medicine n=6, Anaesthesia n=5, Paediatrics n=5, Emergency medicine n=2, Gastroenterology n=2, Other specialist n=5, Non-specialist n=23<br>**Inclusion criteria:** none<br>**# invited:** n.a. convenience sample -> snowball technique:<br>**# baseline:** n=58<br>**# follow-up:** n.a.<br>**Age at baseline:**<br>20-30 years: n=28<br>31-40 years: n=25<br>41-50 years: n=4<br>61-70 years: n=1<br>**% of females:** 39.7%<br>**Response:** n.a. (convenience sample)<br>**Loss-to-follow-up:** n.a.		<u>Self-stigma (anticipated)</u>	**Unfavourable health consequences associated with stigmatization:** Stress. Results are shown in Tab. 2.<br><br>**Stigmatization studied in association with return-to-work:** was not investigated	- recruitment procedure: convenience sample<br>- confounders<br>- analysis method
7272<br>Uvais, 2020, India<br><br>High risk	**Study design:** Cross-sectional<br>**Time of Study:** 26. April-07. July 2020	**Job description:** Haemodialysis staff<br>Dialysis technicians: 55.2%<br>Dialysis nurses: 44.8%<br>**Inclusion criteria:** none<br>**# invited:** n.a. snowball technique	Being dialysis staff during COVID-19 pandemic	Perceived stigma: stigma scale with 13 items (each scored on a 5-point Likert scale, total score 0 – 52; total score ≥26: high levels of perceived stigma)<br><br>**Form of stigmatization:** <u>Self-stigma (perceived):</u>	**Prevalence of stigmatization:**<br><br><u>Self-stigma (perceived and anticipated)</u><br><br>High levels of perceived stigma: n=132 (54.6%), mean (SD)= 25.3 (8.1)<br><br>**Conditions increasing work-related stigmatization:** was not investigated	+ informed consent<br>+ ethical approval by the Iqraa International Hospital and Research Centre Institutional Review Board<br>+ no conflict of interest

General	Study	Population	Exposure	Outcome	Results	Comments
	**Follow-up mean, range:** n.a	**# baseline:** n=335<br>**# follow-up:** n.a.<br>**Age at baseline:** 18-30 years: 77%<br>**% of females:** 72.8%<br>**Response:** n.a. (convenience sample)<br>**Loss-to-follow-up:** n.a.		discrimination, social exclusion<br><br>Self-stigma (anticipated)	**Measures to prevent/ deal with stigmatization at work:** was not investigated<br><br>**Unfavourable health consequences associated with stigmatization:** Stress. Results are shown in Tab. 2.<br><br>**Stigmatization studied in association with return-to-work:** was not investigated	+ funding stated (none)<br>+ outcome: measurement of stigma with a 13-item instrument<br>+ measurement of stress with a validated instrument<br><br>- recruitment procedure: convenience sample<br>- analysis method<br>- investigation of the association between stigmatization and stress only with Pearson's chi-square test<br>- no consideration of any confounders in the analysis of the association between stigmatization and stress<br>- no investigation of a temporal relationship possible due to the cross-sectional design
7284<br>Yadav, 2020, India<br><br>High risk	**Study design:** Cross-sectional<br>**Time of Study:** May - July 2020	**Job description:** Health Care Professionals (HCPs)<br>**Designation:** Resident doctors: n=298<br>Faculty/medical officers: n=97<br>Nursing: n=29	During COVID-19 pandemic (not specified)	Adapted Stigma assessment and reduction of impact (SARI) Stigma scale (Adaption to South-East Asia and COVID-19). Experienced stigma = "encounters with stigmatizing attitude and behaviour from general	**Prevalence of stigmatization:**<br><br>Self-stigma (perceived)<br><br>Table 1: Socio-demographic characteristics of the study participants and perceived stigma related to COVID-19 (n=424)<br><br>Socio-demographic characteristics | Perceived Stigma n (%)<br>Gender |	+ ethical approval from institutional ethics committee<br>+ no conflict of interest<br>+ funding stated (none)<br>+ outcome: validated questionnaire used

<table>
<tr><th>General</th><th>Study</th><th>Population</th><th>Exposure</th><th>Outcome</th><th>Results</th><th>Comments</th></tr>
<tr>
<td></td>
<td>Follow-up mean, range: n.a.</td>
<td>Departments: Medicine and Critical care : n=159
Surgical: n=103
Public Health/community medicine: n=80
Paraclinical departments: n=27
Others: n=55
Inclusion criteria: none
invited: n=1160 (participants received questionnaire)
baseline: n=424 analysed
follow -up: n.a.
Age at baseline: median 29 years
% of females: 57.5%
Response: 36.6%
Loss-to-follow-up: n.a.</td>
<td></td>
<td>population -> perceived stigma

<u>Sections of the questionnaire measuring stigma:</u>
1. Perceived stigma by the doctors at residential areas/hostels as well as workplace.
2. Stigma experienced by them at these places
3. Disclosure concerns about getting oneself tested
4. Apprehensions related to positive tested and fears or concerns related to quarantine and isolation

Forms of stigmatization:
<u>Self-stigma (perceived): discrimination</u>
Have you noticed any difference in the behaviour while you were wearing your lab-coat/uniform or hanging a stethoscope? Have you observed any difference in behaviour of your neighbours? Have you perceived any stigma while shopping for grocery or daily items or vegetables in your</td>
<td>
<table>
<tr><td>Male</td><td>123 (68.3%)</td></tr>
<tr><td>Female</td><td>178 (73.0%)</td></tr>
<tr><td>Designation</td><td></td></tr>
<tr><td>Resident doctors</td><td>222(74.5%)</td></tr>
<tr><td>Faculty/Medical officers</td><td>58(59.8%)</td></tr>
<tr><td>Nursing</td><td>21(72.4%)</td></tr>
<tr><td>Departments</td><td></td></tr>
<tr><td>Medicine and critical care</td><td>112 (70.4%)</td></tr>
<tr><td>Surgical</td><td>81 (78.6%)</td></tr>
<tr><td>Public health/ Community health</td><td>52 (65.0%)</td></tr>
<tr><td>Paraclinical departments</td><td>17 (63.0%)</td></tr>
<tr><td>Others</td><td>39 (70.9%)</td></tr>
</table>

Table.: Self-Stigma (perceived) among healthcare professionals due to COVID-19
<table>
<tr><th></th><th>Not at all</th><th>A little</th><th>Moder-ately</th><th>A lot</th></tr>
<tr><td>Have you noticed any difference in the behaviour while you were wearing your lab-coat/uniform or hanging a stethoscope?</td><td>54%</td><td>30.7%</td><td>11.8%</td><td>3.5%</td></tr>
<tr><td>Have you observed any difference in behaviour of your neighbours?</td><td>54.5%</td><td>30.7%</td><td>10.1%</td><td>4.7%</td></tr>
<tr><td>Have you perceived any stigma while shopping for grocery or daily items or vegetables in your nearby store?</td><td>65.3%</td><td>25.7%</td><td>8.7%</td><td>0.2%</td></tr>
<tr><td>Have you perceived any stigma in your residential society/apartment/colony as you are going to the hospital?</td><td>49.1%</td><td>36.8%</td><td>9.9%</td><td>4.2</td></tr>
</table>

Perceived stigmatization: 82 of 424 (19.3%) -> 48.8% in their “residential societies”, 22.6% in the workplace
</td>
<td>- recruitment procedure: low response (36.6%)
- confounders: age not considered
- analysis method: only descriptive statistics used</td>
</tr>
</table>

<table>
<tr><th>General</th><th>Study</th><th>Population</th><th>Exposure</th><th>Outcome</th><th>Results</th><th>Comments</th></tr>
<tr>
<td></td>
<td></td>
<td></td>
<td></td>
<td>nearby store? Have you perceived any stigma in your residential society/apartment/colony as you are going to the hospital?

<u>Self-stigma (perceived):</u> <u>aggressive behaviour/mobbing</u>

<u>Associative stigma</u></td>
<td>Table: Forms of stigma experienced by the HCPs
<table>
<tr><th>Nature of stigma experienced by participants</th><th>Number of participants n (%) n=82</th></tr>
<tr><td>Rude Behaviour</td><td>57 (69.5%)</td></tr>
<tr><td>Harassment by landlord/neighbours/RWA</td><td>24 (29.3%)</td></tr>
<tr><td>Racial/obscene/derogatory remarks</td><td>26 (31.7%)</td></tr>
<tr><td>Harassment by security personal</td><td>11 (13.4%)</td></tr>
</table>
<u>Associative stigma</u>
11.8% (n=50) family members experienced stigmatization

Conditions increasing work-related stigmatization:
was not investigated

Measures to prevent/ deal with stigmatization at work:
was not investigated

Unfavourable health consequences associated with stigmatization: was not investigated

Stigmatization studied in association with return-to-work: was not investigated</td>
<td></td>
</tr>
<tr>
<td>7220
Zandifar 2020,
Iran

High risk</td>
<td>Study design: Cross sectional
Time of Study: 20 March - 3 April 2020
Follow-up mean, range: n.a.</td>
<td>Job description: HCWs engaged in the field of diagnostic and treatment of COVID-19 patients working in 9 general hospitals:
Physicians: n=80
Nurses: n=543
Technicians: n=253
Inclusion criteria: HCWs in internal medicine and infectious wards</td>
<td>During COVID-19 pandemic (not specified)</td>
<td>Stigma: 22-item modified stigma-related questionnaire adopted from the HIV Stigma Scale (5-point Likert scale: strongly disagree to strongly agree)

Form of stigmatization:
<u>Self-stigma (perceived):</u> <u>discrimination</u></td>
<td>Prevalence of stigmatization:

<u>Self-stigma (perceived):</u>

Tab. 3: Median (IQR) of the Total and Subscale PTSS Score According to Demographic Characteristics
<table>
<tr><th colspan="2">Stigma</th><th>Median</th><th>Inter Quartile Range</th><th>p</th></tr>
<tr><td rowspan="2">Sex</td><td>Male</td><td>26</td><td>15.25</td><td rowspan="2">0.01*</td></tr>
<tr><td>Female</td><td>27</td><td>17</td></tr>
<tr><td rowspan="2">Frontline</td><td>Yes</td><td>28</td><td>17</td><td rowspan="2">0.006*</td></tr>
<tr><td>No</td><td>25</td><td>17</td></tr>
<tr><td rowspan="2">Occupation</td><td>Physician</td><td>29</td><td>19.5</td><td rowspan="2">0.04*</td></tr>
<tr><td>Nurse</td><td>26</td><td>17</td></tr>
</table></td>
<td>+ recruitment procedure: multistage sampling method (proportional random sampling)
+ high response
+ outcome: usage of validated instruments to measure PTSD and stigma
+ ethics approval from the ethics committee of Alborz University of Medical Sciences</td>
</tr>
</table>

<table>
<tr><th>General</th><th>Study</th><th>Population</th><th>Exposure</th><th>Outcome</th><th>Results</th><th>Comments</th></tr>
<tr>
<td></td>
<td></td>
<td>and intensive care units, in contact with patients with COVID-19, working full-time or part-time
 # invited: n=971
 # baseline: n=894
 # follow -up: n.a.
Age at baseline:
<30: n=296
31-40: n=374
>40: n=210
% of females: 71.4%
Response: 92%
Loss-to-follow-up: n.a.</td>
<td></td>
<td></td>
<td><table><tr><td></td><td>Technician</td><td>22</td><td>32</td><td></td></tr></table>* Statistically significant

Conditions increasing work-related stigmatization: was not investigated

Measures to prevent/ deal with stigmatization at work: was not investigated

Unfavourable health consequences associated with stigmatization: PTSD, Results are shown in Tab. 2.

Stigmatization studied in association with return-to-work: was not investigated</td>
<td>+ no conflict of interest

- no information on funding
- investigation of the association between PTSD and stigma only with correlation analysis
- confounders: age not considered</td>
</tr>
<tr>
<td>905
Zhu,
2020,
China

High risk</td>
<td>**Study design:** Cross-sectional
Time of Study: 8 - 10 February 2020
Follow-up mean, range: n.a.</td>
<td>**Job description:** HCWs employed in a hospital directly providing services to confirmed or suspected COVID-19 patients
Doctors: n=1004
Nurses: n=3417
Medical technicians: n=641
Inclusion criteria: none
 # invited: n=6568
 # baseline: n=5062
 # follow-up: n.a.
Age at baseline:
19-29: 40.1%
30-49: 56.4%,
>49 years: 3.5%
% of females: 85.0%</td>
<td>COVID-19 outbreak (2 weeks after the authority in Wuhan suspended all public transport)</td>
<td>Feeling that family and friends avoid contact because of work: 1 item question (answer options: agree, disagree, unsure)

Form of stigmatization: <u>Self-stigma (perceived): social exclusion</u></td>
<td>**Prevalence of stigmatization:**
Table 2: Do you feel that family members and friends have avoided contact with you because of your work?<table><tr><th>Answer options</th><th>n</th><th>%</th></tr><tr><td>Disagree</td><td>2,513</td><td>49.6</td></tr><tr><td>Agree</td><td>987</td><td>19.5</td></tr><tr><td>Unsure</td><td>1,562</td><td>30.9</td></tr></table>**Conditions increasing work-related stigmatization:** was not investigated

Measures to prevent/ deal with stigmatization at work: was not investigated

Unfavourable health consequences associated with stigmatization: Depression, anxiety, and psychological stress. Results are shown in Tab. 2.

Stigmatization studied in association with return-to-work: was not investigated</td>
<td>+ study followed STROBE guidelines
+ ethical approval by the institutional ethics board of Tongji Hospital
+ no conflict of interest
+ funding from non-profit organizations
+ census sampling (all healthcare workers of the hospital invited)
+ recruitment procedure: high response
+ pilot-testing of the questionnaire
+ measurement of depression, anxiety, and stress with validated instruments</td>
</tr>
</table>

General	Study	Population	Exposure	Outcome	Results	Comments
		**Response:** 77.1%<br>**Loss-to-follow-up:** n.a.				+ using multivariate logistic regression analysis in order to investigate the association between stigmatization and mental health outcomes<br><br>- outcome: measurement of stigmatization with a single question<br>- no consideration of any confounders in the analysis of the association between stigmatization and mental health outcomes<br>- analysis method<br>- no investigation of a temporal relationship possible due to the cross-sectional design

n = sample size, n.a. = not applicable, n.r. = not reported, SD = standard deviation
