# Supplementary material for: Stigmatization from Work-Related COVID-19 Exposure: A Systematic Review with Meta-Analysis
Source: Int J Environ Res Public Health. 2021 Jun 8;18(12):6183. doi: 10.3390/ijerph18126183 (PMC8226580; doi:10.3390/ijerph18126183)
Supplement: Supplementary file 1 [file ijerph-18-06183-s001.zip › Supplementary_ijerph-1235285/Table_S5.pdf]

General	Study	Population	Exposure	Outcome	Results	Comments
**#<br>First Author, Year, Country**	**Study design:<br>Time of Study:<br>Follow-up mean, range:**	**Job description:<br>Inclusion criteria:<br># invited:<br># baseline:<br># follow -up: Age at baseline:<br>F : M at baseline:<br>Response:<br>Loss-to-follow-up:**	*Describe how exposure was measured*	*Describe how outcome was measured*<br><br>**Form of stigmatization:**	**Prevalence of stigmatization:**<br><br>**Conditions increasing work-related stigmatization:**<br><br>**Measures to prevent/ deal with stigmatization at work:**<br><br>**Unfavourable health consequences associated with stigmatization:**<br><br>**Stigmatization studied in association with return-to-work:**	
7270<br>Bhatt, 2020, Nepal	**Study design:** Qualitative phenomenological approach with focus group discussions (n=8) and in-depth-interviews (n=40)<br>**Time of Study:** March - June 2020<br>**Follow-up mean, range:** n.a.	**Job description:**<br>Teachers: n=7<br>Students: n=9<br>Security personnel: n=9<br>Head of household: n=9<br>Leaders: n=14<br>Health workers: n=15<br>Homemaker: n=9<br>Others: n=16<br>**Inclusion criteria:** 18-60 years old<br>**Interviews:** individuals who were at the forefront in the community (e.g. Healthcare workers, police, school teachers)<br>**Focus groups:** 1 participant from a house<br>Exclusion: <18 years or >60 years, being pregnant, or suffering from infectious disease or having underlying conditions	During COVID-19 pandemic (not specified)	Social discrimination and stigma: emerged as one subtheme in interviews and focus group discussions (overarching theme: personal and societal consequences of CI was social discrimination and stigma<br><br>**Form of stigmatization:**<br>Self-stigma (perceived): discrimination<br><br>Associative stigma: discrimination	**Prevalence of stigmatization:**<br>- Especially health care workers, and their family members were discriminated<br>- Healthcare workers were expelled from their rent houses; moreover, they were denied proper food and lodging in the hotels<br>- One health care worker reported: *“The house owner has told me to leave the rented room as soon as possible, blaming me to be a major source of COVID-19 spread as I am a health worker. Where should I go all of a sudden? I feel regretful; I made a wrong decision by choosing this profession.”*<br><br>**Conditions increasing work-related stigmatization:** was not investigated<br><br>**Measures to prevent/ deal with stigmatization at work:** was not investigated<br><br>**Unfavourable health consequences associated with stigmatization:** was not investigated<br><br>**Stigmatization studied in association with return-to-work:** was not investigated	+ informed consent<br>+ ethical approval from the Ethical Review Board of the Nepal Health Research Council<br>+ no conflict of interest<br>+ funding stated (none)<br>+ validation of the tool was ensured by the index of item-objective congruence and consultation with experts<br>+ usage of two different interview styles<br>+ pretesting prior to the study<br>+ use of a maximum variation sample method, thus, representation of participants from diverse professions

General	Study	Population	Exposure	Outcome	Results	Comments
		**# invited:** n.a. convenience sampling (usage of a maximum variation sampling method)<br>**# baseline:** n=88<br>**# follow -up:** n.a.<br>**Age at baseline:**<br>**Focus group discussions (FGD):**<br>18–30 years: n=12<br>31–40 years: n=18<br>41–50 years: n=10<br>51–60 years: n=8<br>**In-depth-Interviews (IDI):**<br>18–30 years: n=12<br>31–40 years: n=14<br>41–50 years: n=8<br>51–60 years: n=6<br>**% of females:**<br>FDG: 50%<br>IDI*:* 45%<br>**Response:** n.a. convenience sampling<br>**Loss-to-follow-up:** n.a.				- convenience sampling of the municipalities and participants (but random selection of wards)<br>- no generalizable results
7281 Crowe, 2020, Canada	**Study design:** Convergent parallel mixed method study (questionnaire survey, semi-structured qualitative interviews), only interview data contain information on stigma	**Job description:** Critical Care Registered Nurses (CCRN) providing direct patient care in the intensive care and high acuity units in an academic teaching hospital<br>**Inclusion criteria:** registered nurses working in intensive	During the initial phase of the COVID-19 pandemic	Social discrimination and stigma emerged as one topic in the interviews<br><br>**Form of stigmatization:**<br><u>Self-stigma (perceived): discrimination, social exclusion,</u>	**Prevalence of stigmatization:**<br>- Some even reported feeling stigmatized by friends and family for working in the COVID unit<br>- They reported feeling isolated and shunned by family and community members who feared that the nurses would infect them with COVID-19<br>- One participant stated *"We've had people yelling at us for being too close and uh, cause we're out walking the dog, right."*<br>- Others reported that they were not living with family during the pandemic, or had stopped sharing bedrooms with their spouse due to the family's fear of them *"bringing COVID home"*.	+ ethical approval from the Fraser Health Authority Research Ethics Board and the University of British Columbia harmonized ethics board<br>+ high response of the survey<br>+ funding stated (none)

General	Study	Population	Exposure	Outcome	Results	Comments
	**Time of Study:** May 2020<br>**Follow-up mean, range:** n.a.	care unit or high acuity unit<br>**# invited:** n=242<br>**# baseline:** Interviews: n=15<br>**# follow -up:** n.a.<br>**Age at baseline:** Interviews: mean: 38.8 years (range: 26–61 years)<br>**% of females:** Interviews: 100%<br>**Response:** 45%<br>**Loss-to-follow-up:** n.a.		aggressive behaviour/mobbing	**Conditions increasing work-related stigmatization:** was not investigated<br><br>**Measures to prevent/ deal with stigmatization at work:** was not investigated<br><br>**Unfavourable health consequences associated with stigmatization:** was not investigated<br><br>**Stigmatization studied in association with return-to-work:** was not investigated	+ no conflict of interest<br><br>- convenience sampling of the interviews
7271 Dye, Worldwide, 2020	**Study design:** Cross-sectional study with qualitative part, conducted around the world<br>**Time of Study:** 6 April - 29 May 2020<br>**Follow-up mean, range:** n.a.	**Job description:** n.r.; all<br>**Inclusion criteria:** none<br>**# invited:** convenience sample -> recruitment via mTURK (access to a multilingual digital workforce) and Facebook, Instragram, facebook Audience Network;<br>**# baseline:** n=7411 from 173 countries<br>**# follow-up:** n.a.<br>**Age at baseline:** not reported<br>**% of females:** not reported<br>**Response:** n.a. convenience sample<br>**Loss-to-follow-up:** n.a.	During COVID-19 pandemic -><br>Working in health care setting	1 item, derived from the: KFF Corona-virus Poll 26 question: Do you or anyone in your household work in a healthcare delivery setting, such as a doctor's office, clinic, hospital, nursing home or dentist's office?<br><br>Have you or a family member been harassed, bullied or hurt because of coronavirus? (yes, no/don't know) –> If yes, participants were asked to explain (open-end question)	**Prevalence of stigmatization:**<br><br>Qualitative part:<br>Tab. 3: harassment/bullied/hurt excerpts related to COVID-19 related to work/ happened in work: n=21<br><br>Self-stigma (perceived):<br>"My sister-in- law made the mistake of going out in the street wearing surgical clothes (she is neither a doctor nor a nurse but works in a hospital…) and people attacked her on the street (only insults, but he was afraid". (Female participant, in her 30s, Central America)<br><br>"My niece, who is a doctor, is harassed at her home by the neighbours telling her to get out of there …". (South American participant)<br><br>"My relatives are engaged in the health sector, and they suffered harassment for requesting labor supplies". (Male participant, in his 50s, Central America)<br><br>"Unable to enter a supermarket because of being a nurse". (Male participant, in her 30s, Southeast Asia)<br><br>"I've been ridiculed for trying to enforce physical distancing at my place of work. I've been purposely sneezed on at very close	+ ethic approval from University of Rochester's Research Subjects Review Board<br>+ no conflict of interest<br>+ Funding stated<br><br>- convenience sample<br>- only one question for harassment, bully, not validated

General	Study	Population	Exposure	Outcome	Results	Comments
				**Form of stigmatization:**<br>Self-stigma (perceived): aggressive behaviour/mobbing<br><br>Associative stigma: aggressive behaviour/mobbing	distance. There have been many rude and impatient customers that have been difficult to deal with.” (Female participant, in her 50s, Northern America)<br><br>“Going out with a medical uniform is complicated, people are very ignorant and attack us”. (Female participant, in her 40s, Central America)<br><br>“There have been isolated reports from some parts of the country that doctors have been harassed and asked to leave their apartment buildings”. (Female participant, in her 30s, South Asia)<br><br>Associative stigma:<br>“My son is a doctor and they have insulted me on the street accusing him of bringing the virus”. (Male participant, in his 50s, Central America)<br><br>**Conditions increasing work-related stigmatization:** was not investigated<br><br>**Measures to prevent/ deal with stigmatization at work:** was not investigated<br><br>**Unfavourable health consequences associated with stigmatization:** was not investigated<br><br>**Stigmatization studied in association with return-to-work:** was not investigated	
910 Fawaz 2020, Lebanon	**Study design:** Explorative qualitative, phenomenological data analysis<br>**Time of Study:** n.r.<br>**Follow-up mean, range:** n.a	**Job description:** HCWs working at various COVID-19 units, and who were quarantined due to the occupational exposure to COVID-19 cases<br>Nurses: n=9<br>Physicians: n=4<br>**Inclusion criteria:**	Being quarantined following exposure to COVID-19	“Stigma of being infected” was 1 of 4 topics that emerged during the interviews semi-structured interviews, until data saturation was reached	**Prevalence of stigmatization:**<br><br>- Participants reported that working in a hospital in the time of the outbreak gave rise to negative reactions from people at first, and when they knew they were working in the coronavirus unit, it was even more exaggerated<br><br>**Quote from a nurse:** *“Even before working in the COVID-19 unit, my friends and some family members refused to see me because I might be carrying the virus. When I volunteered, the situation got worse especially that I was quarantined. Everyone was perceiving*	+ informed consent<br>+ approval by the Institutional Review Board<br>+ no conflict of interest<br>+ research received no funding

General	Study	Population	Exposure	Outcome	Results	Comments
		**# invited:** n.a. convenience sample (snowball technique)<br>**# baseline:** n=13<br>**# follow-up:** n.a.<br>**Age at baseline:** n.r.<br>**% of females:** n.r.<br>**Response:** n.a. convenience sample (snowball technique)<br>**Loss-to-follow-up:** n.a.		**Form of stigmatization:** <u>Self-stigma (perceived): discrimination, social exclusion</u>	*me as infected. I don't know how they would receive me after the outbreak . . . would they still have the same fear?"*<br><br>**Quote from another nurse:** *"At the start of the outbreak, I didn't even allow myself to get in contact with other people especially my old family members like grandparents; they are vulnerable and one never knows . . . but like at a certain point, my aunt was standing way far from me when I saw her in the street . . . she felt like I was infected . . . treated me as if I am the virus . . . it was weird. After working in the COVID-19 unit . . . man it was bad! I kept getting WhatsApp messages joking about me being infected with corona."*<br><br>**Quote from a physician:** *"Before quarantine, it was very hard to get a lift . . . no one accepted to take me to the hospital they were all concerned and thought that the area surrounding the hospital is infested and they would even catch the virus from the air surrounding the hospital. Now that I am quarantined for working in the COVID-19 unit . . . my friends and family do have an inclination to treat me as I am contagious . . . it brings frustration and sometimes even anger really."*<br><br>**Conditions increasing work-related stigmatization:** was not investigated<br><br>**Measures to prevent/ deal with stigmatization at work:** was not investigated<br><br>**Unfavourable health consequences associated with stigmatization:** was not investigated<br><br>**Stigmatization studied in association with return-to-work:** was not investigated	+ explorative character of the study design<br><br>- convenience sample
7286 Feroz, 2020, Pakistan	**Study design:** Qualitative study<br>**Time of Study:** April - May 2020<br>**Follow-up mean, range: n.r**	**Job description:** Key informants KIIs (senior management and hospital leadership, directly or indirectly involved with the management of	During COVID-19 pandemic (not specified)	Self-developed semi-structured interviews and a purposive sampling approach:<br>-interviews: KIIs, IDIs<br>-piloted (KIIs: n=2, IDIs: n=2)	**Prevalence of stigma:**<br><br>Theme: Challenges faced by frontline healthcare providers working in the COVID-19 wards<br><br>Subtheme: Stigma associated with healthcare providers working in COVID-19 wards<br><br><u>Self-stigma (anticipated):</u>	+ethic approval (Aga Khan University Ethical Review Committee (AKUERC) – [2020-3694-9056])<br>+ no conflict of interest

General	Study	Population	Exposure	Outcome	Results	Comments
		COVID-19 patients): n=19<br>In-depth interviews IDIs (frontline healthcare providers, directly involved in the care of COVID-19 patients): n=12<br>**Inclusion criteria:** n.r<br>**# invited:** n.r<br>**# baseline:** Total: n=31<br>Key informants: n=19<br>In-depth interviews: n=12<br>**# follow -up:** n.a<br>**Age at baseline:** n.r<br>**% of females:** 71%<br>**Response:** n.r.<br>**Loss-to-follow-up: n.a**		Interview involved a discussion on perceptions about COVID-19 outbreak<br><br>**Form of stigmatization:** Self-stigma (anticipated)	*"I know that in some cases health care workers do not tell their families and communities that they are working with COVID-19 patients. They fear that this will cause unnecessary panic and people may view them differently" (KII-19, Associate Professor)*<br><br>**Conditions increasing work-related stigmatization:** was not investigated<br><br>**Measures to prevent/ deal with stigmatization at work:**<br><br>was not investigated<br><br>**Unfavourable health consequences associated with stigmatization:** was not investigated<br><br>**Stigmatization studied in association with return-to-work:** was not investigated	+ funding state (none)<br><br>-no information on recruitment procedure
7268 Hien 2020, Germany	**Study design:** Qualitative study (interviews, grounded theory)<br>**Time of Study:** May - July 2020<br>**Follow-up mean, range:** n.a.	**Job description:** Nurses in clinics and retirement homes<br>**Inclusion criteria:** n.r.<br>**# invited:** n.a. convenience sampling<br>**# baseline:** n=26<br>**# follow -up:** n.a.<br>**Age at baseline:** 30-50 years: n=16, >50 years: n=10<br>**% of females:** 73.1%	During COVID-19 pandemic (not specified)	Discrimination because of COVID-19 using interviews<br><br>**Form of stigmatization:** Self-stigma (perceived): discrimination, social exclusion<br><br>Associative stigma: discrimination	**Prevalence of stigmatization:**<br>- Experiences of discrimination because nurses are perceived as a possible risk of infection<br>- Nurses experienced – depending on region and social environment – irritation, stigmatization, and in specific cases even exclusion<br>- A hospital nurse had to experience that the mere fact that she works in a corona ward can result in massive discrimination against her entire family (husband was no longer allowed in the office)<br><br>**Conditions increasing work-related stigmatization:** was not investigated<br><br>**Measures to prevent/ deal with stigmatization at work:** was not investigated	+ public funding<br>+ ethics approval from the German Social Accident Insurance Institution for the Health and Welfare Services<br>+ informed consent<br><br>- convenience sampling<br>- no information on conflict of interest

General	Study	Population	Exposure	Outcome	Results	Comments
		**Response:** n.a. convenience sampling<br>**Loss-to-follow-up:** n.a.			**Unfavourable health consequences associated with stigmatization:** was not investigated<br><br>**Stigmatization studied in association with return-to-work:** *yes (describe results) or no*<br>No	
1033<br>Kackin, Turkey, 2020	**Study design:** Qualitative study<br>**Time of Study:** 9 - 12 May<br>**Follow-up mean, range:** n.a.	**Job description:** Nurses<br>**Inclusion criteria:** Having cared for patients diagnosed with COVID-19<br>**# invited:** convenience sample (snowball technique)<br>**# baseline:** 10<br>**# follow-up:** n.a.<br>**Age at baseline:** 46.7 (8.4) years<br>**% of females:** 50.2%<br>**Response:** n.a. convenience sample (snowball technique)<br>**Loss-to-follow-up:** n.a.	During COVID-19 pandemic (not specified)	Semi-structured interview<br>1. What experiences did you have while caring for a patient diagnosed with COVID-19?<br>2. What psychosocial problems have you experienced when caring for a patient diagnosed with COVID-19?<br>3. Could you please explain your views on your coping strategies when caring for a patient diagnosed with COVID-19?<br><br>**Form of stigmatization:**<br><u>Self-stigma (perceived): social exclusion</u><br><br><u>Associative stigma</u>	**Prevalence of stigmatization:**<br>Nurse 9: '*My social relationships have decreased a lot, I cannot see my friends, my best friend was supposed come visit me after a month, but those in the unit, where my friend worked, said that he/ she could not visit*<br>Nurse 9: *My friend came to me really demoralized . . . He/she did not tell anyone about his/her visit . . . When he/she returned, he/she acted as if he/she had not visited me . . . This situation wears me down emotionally*<br><br>**Conditions increasing work-related stigmatization:** was not investigated<br><br>**Measures to prevent/ deal with stigmatization at work:** was not investigated<br><br>**Unfavourable health consequences associated with stigmatization:** The study investigates experiences and psychological consequences of nurses caring for COVID-19 patients in general.<br><br>**Stigmatization studied in association with return-to-work:** was not investigated	+ no conflict of interest<br>+ funding stated (none)<br><br>- convenience sampling
7289<br>Kalateh Sadati 2021,	**Study design:** Qualitative study (semi-structured interviews via	**Job description:** Nurses working in hospitals specified	During COVID-19 pandemic (not specified)	Question related to stigma: What problems did you	**Prevalence of stigmatization:**<br><br><u>Perceived stigma</u>	+ ethic approval from ethics committee of Shiraz University of

General	Study	Population	Exposure	Outcome	Results	Comments
Iran	phone or face-to-face)<br>**Time of Study:** March 2020<br>**Follow-up mean, range:** n.a.	for COVID-19 treatment<br>**Inclusion criteria:** None<br>**# invited:** n.r.<br>**# baseline:** n=24<br>**# follow-up:** n.a.<br>**Age at baseline:** n.r.<br>**% of females:** n.r.<br>**Response:** n.r.<br>**Loss-to-follow-up:** n.a.		have in the ward or the hospital?<br><br>Stigma emerged as one topic in the interviews<br><br>**Form of stigmatization:**<br>Self-stigma (perceived): discrimination, social exclusion	*"My kids run away from me. They say you have Corona and I get stress, I tell myself not to get sick. They put me away. My kids run away from me. They give me something while keeping off"* (Mahin, Qazvin)<br><br>"*My brother could have met me while keeping off, but he didn't come and I was upset.*" (Samira)<br><br>*„Tow of our colleague were going to the hospital. One of them got in a taxi and the driver asked him where she was going, When she told him to get her to the hospital, he asked her to get off. The same happened to another colleague when a driver, after knowing her destination –our hospital- did not allow her to get in the taxi"* (a head Nurse, Shiraz)<br><br>**Conditions increasing work-related stigmatization:** was not investigated<br><br>**Measures to prevent/ deal with stigmatization at work:** was not investigated<br><br>**Unfavourable health consequences associated with stigmatization:** was not investigated<br><br>**Stigmatization studied in association with return-to-work:** was not investigated	Medical Sciences (IR.SUMS. REC.1395.S1249)<br><br>- no information about recruitment and study population<br>- funding not stated<br>- conflict of interest not stated
7276<br>Lee 2020, South Korea	**Study design:** Qualitative study<br>**Time of Study:** 8 June - 25 September 2020<br>**Follow-up mean, range:** n.a	**Job description:** COVID-19-designated hospital nurses who provided care for patients<br>**Inclusion criteria:** Having at least one year of patient care experience, having at least 2 months of work experience in a COVID-19 isolation ward (nurse managers who were	During COVID-19 pandemic (not specified)	"Being Treated Like a Virus" (avoidance, discrimination, stigmatization) was one subtheme and "Strict Social Standards" another subtheme of the overarching theme "low morale" (which was 1 of 9 overarching themes that emerged	**Prevalence of stigmatization:**<br><br>**1. Subtheme "Being Treated Like a Virus":**<br>- Participants shared their experiences of feeling like they had become people whom others avoided because they were nurses at a COVID-19-designated hospital, which led to discrimination in their daily lives<br>- in addition, the participants' families had a similar experience even though they were not infected<br>- the stigma broke the hearts of the participants, and made them hide the fact that they were nurses at a COVID-19-designated hospital and restricted their own radius of action	+ approval by the Institutional board of Catholic University of Pusan<br>+ informed consent<br>+ no conflict of interest<br>+ funding from non-profit organizations<br>+ explorative character of the study design

General	Study	Population	Exposure	Outcome	Results	Comments
		not directly involved in patient care were excluded)<br>**# invited:** n.a. snowball technique<br>**# baseline:** n=18<br>**# follow-up:** n.a.<br>**Age at baseline:**<br>20-29 years: n=8<br>30-39 years: n=8<br>40-49 years: n=2<br>**% of females:** 100%<br>**Response:** n.a. convenience sample (snowball technique)<br>**Loss-to-follow-up:** n.a.		during the in-depth interviews (undertaken until data saturation was reached))<br><br>**Form of stigmatization:**<br><u>Self-stigma (perceived): Stereotypes, discrimination, social exclusion</u><br><br><u>Associative stigma: social exclusion</u>	**Quote from a nurse:** *"I can't tell others about working at this hospital because when I asked the taxi driver to take me to the hospital, he asked me if I worked there, and then he told me to get out. They don't even deliver food to the hospital. I wasn't infected with COVID-19, and I didn't do anything wrong, but I had to stay at home. Because people don't want contact with me."*<br><br>**Quote from another nurse:** *"My child goes to preschool. I told the teacher at preschool that I won't be sending my child there for a while, just in case my child might get infected because of me. The teacher seemed relieved and happy when I mentioned that."*<br><br>**2. Subtheme "Strict Social Standards":**<br><br>- Participants felt burdened and even angry at times due to the unusually strict social standards directed only at nurses<br><br>**Quote from a nurse:** *"When a nurse was diagnosed with COVID-19, it was from taking care of patients. However, she became a target of criticism, questioning whether she took off her PPE properly. Seeing that made me really angry. If I were put in that position, I would quit …"*<br><br>**Conditions increasing work-related stigmatization:**<br>- in particular, they mentioned that they thought about quitting after seeing critical posts flooding social networks and the attention received by a nurse colleague nurse diagnosed with COVID-19<br><br>**Quote from another nurse:** *"When I read the news, there were comments about why do healthcare professionals who deal with confirmed patients go out to eat or go to the gym … Honestly, we can go out to eat. We really get hurt when we see malicious comments like that."*<br><br>**Measures to prevent/ deal with stigmatization at work:** was not investigated<br><br>**Unfavourable health consequences associated with stigmatization:** was not investigated	- convenience sample

General	Study	Population	Exposure	Outcome	Results	Comments
					**Stigmatization studied in association with return-to-work:** was not investigated	
7277<br>Reazee,<br>Iran, 2020	**Study design:** Qualitative study (phone interviews via WhatsApp)<br>**Time of Study:** September - October 2020<br>**Follow-up mean, range:** n.a.	**Job description:** Nurses<br>**Inclusion criteria:** Full time employment in the COVID-19 wards, having at least one month of experience in caring for COVID-19 patients. Selection of participants by maximum variation in age, gender, education, work experience, history of caring for COVID-19 patients, and ward<br>**# invited:** n.r.<br>**# baseline:** n=24<br>**# follow-up:** n.a.<br>**Age at baseline:** range 27-49 years<br>**% of females:** 62.5%<br>**Response:** n.r.<br>**Loss-to-follow-up:** n.a.	During COVID-19 pandemic (not specified)	Main questions: How would you describe caring for people with COVID-19? What are the ethical challenges of providing care in your opinion?<br><br>**Form of stigmatization:**<br><u>Self-stigma (perceived): stereotypes, discrimination</u><br><br><u>Associative stigma: stereotypes, discrimination</u>	**Prevalence of stigmatization:**<br><br><u>Self-stigma (perceived)</u><br>"after providing care to COVID-19 patients, their professional dignity has been endangered by family, friends, and relatives. This ultimately leads to a threat to their social respect by the community:<br><br>Theme: A: The treats to professional values<br>Subtheme: A.2: A stigmatized public image about COVID-19<br><br>"*It's interesting that my father asks me to get away from these patients and recommends me to leave my job. He asks me if I am short in money that I have to care for these dying patients in the deathward. He says I would lose my life. For these stigmata, all my colleagues want to change their workplace and go to another ward*". [Participant No. 22]<br><br><u>Self-stigma (perceived)/Associative stigma</u><br>"*In the community, we have been stigmatized as corona-infected nurses, and we are known by this name among friends and relatives. Neighbors think that I have been infected with coronavirus because of working with COVID patients, so our family is also regarded as a corona-infected family. I think there has been a kind of cultural sensitivity towards nursing*". [Participant No. 11]<br><br>**Conditions increasing work-related stigmatization:** was not investigated<br><br>**Measures to prevent/ deal with stigmatization at work:** was not investigated<br><br>**Unfavourable health consequences associated with stigmatization:** was not investigated<br><br>**Stigmatization studied in association with return-to-work:** was not investigated	+ ethic approval<br><br>- no information on number of invited workers<br>- conflict of interest not stated<br>- funding not stated

General	Study	Population	Exposure	Outcome	Results	Comments
5388<br>Rizvi Jafree, 2020, Pakistan	**Study design:** Qualitative study<br><br>**Time of Study:** May 2020<br><br>**Follow-up mean, range:**<br>**n.a**	**Job description:** n.r.<br>**Inclusion criteria:** COVID-19 negative persons at the time of the study with COVID-19 positive relatives<br>Persons with relatives being in currently in quarantine, admitted to hospital or discharged from hospital in the last 3 months<br>**# invited:** n=72 **(**all families being listed).<br>**# baseline:** n = 20<br>**# follow -up: n.a**<br>**Age at baseline:**<br>Age group (years)<br>21–26: n=4<br>32–36:n=6<br>40–45:n=5<br>50–55:n=3<br>61–62:n=2<br>**F : M at baseline:** 50%<br>**Response:** 41.6%<br>**Loss-to-follow-up: n.a**	COVID-affected families admitted at three government-allocated hospital quarantine wards (February - April 2020)	Self-developed, semi-structured open-end survey, 9 items<br><br>**Form of stigmatization:**<br>Self-stigma (perceived): discrimination	**Prevalence of stigmatization:**<br><br>Stigma related themes and subthemes (in association with the workplace):<br><br>Theme: Doctors facing stigma from other doctors<br>*"My colleagues are suspicious of me, despite my negative test results and strict observance of a PPE [personal protective equipment] kit. This has been painful since my colleagues know that I would not jeopardize them or my patients....We are doctors. I am upset that this behavior has been shown to me by the medical fraternity"*<br><br>Theme: Discrimination by employer<br>*"I am worried about my husband's job and our household income. My husband's boss at work is not accepting the fact that his reports are negative now"*<br><br>*"As a security officer at a bank, my son got it (coronavirus) on duty. Now that he has recovered, his contract has not been resumed. We cannot complain to the government as the employers draft 3-monthcontracts. This way they can dismiss us without explanations when itis convenient for them."*<br><br>**Conditions increasing work-related stigmatization:**<br>Not reported<br><br>**Measures to prevent/ deal with stigmatization at work:** not reported<br><br>**Unfavourable health consequences associated with stigmatization:** not reported<br><br>**Stigmatization studied in association with return-to-work:** *NO*	+ ethic approval**:** From Institutional Review Board of Forman Christian College University (Lahore, Pakistan)<br><br>- conflict of interests not stated<br>- funding not stated<br>- response 41.6%
5386<br>Zolnikov 2020, US, Kenya,	**Study design:** Qualitative phenomenological study	**Job description:** First responders, and HCW<br>Nurses: n=14	During COVID-19 pandemic (not specified)	Stigma semi-structured interviews	**Prevalence of stigmatization:**<br>- Many participants discussed aspects of stigma, although never directly associated themselves with stigma or being stigmatized	+ study protocol and ethics review approved by

General	Study	Population	Exposure	Outcome	Results	Comments
Ireland and Canada	**Time of Study:** n.r.<br>**Follow-up mean, range:** n.a.	Physicians: n=3<br>Firefighters and paramedics: n=3<br>Police officers: n=3<br>Nurse tech, behavioral therapist, orthodontist, dialysis technician, technician in medical surgery, data specialist, and emergency medical technician: each n=1<br>**Inclusion criteria:** above the age of 18 years, being health care worker or first responder, and worked during the COVID-19 pandemic<br>**# invited:** n.a., convenience sampling<br>**# baseline:** n=31<br>**# follow -up:** n.a.<br>**Age at baseline:** mean: 36.1 years (range: 23-57 years)<br>**% of females:** females: n=18, males: n=13<br>**Response:** n.a. convenience sampling<br>**Loss-to-follow-up:** n.a.		**Form of stigmatization:**<br>Self-stigma (perceived); discrimination, social exclusion, aggressive behaviour/mobbing<br><br>Self-Stigma (internalized)	→ Quote: *"I haven´t had any [stigma] …I [did] stay at the abandoned other house that we have … for about 2 weeks and, you know tried not to come home"*<br><br>- Participants used various negative words to describe how they felt during the pandemic<br>→ Quote 1*: "like I was infected, … life I was dirty"*<br>→ Quote 2*: "contaminated"*<br>- Participants used various negative words to describe how they were regarded<br>→ Quote 1*: "They treat me like I had the plaque"*<br>→ Quote 2*: "[I feel] "dirty." My clothes are "dirty", my hair is "dirty", my shoes are "dirty," . . . everything with me has been "stained" with COVID 19 . . . including my body. I know that the "dirtiness" of it all isn't me personally, but it is hard to turn away and not take it personal and feel helpless....I guess I just do not want to feel dirty anymore; it's draining"*<br><br>- Participants believed that being isolated was justified<br>→ Quote: "*Well, I mean, . . . it's understandable. People were just scared, I think"*<br>- Being isolated negatively affected participants<br>→ Quote: *"Well, initially I was quite sad to [be treated differently] because it was all of a sudden. But then . . . it becomes the norm"*<br>- Isolation and directed fear came from different layers of people, including the general public, friends, and family<br>- Reactions from the pubic ranged and included arm's-length support<br>→ Quote : *"[I received] cards, like kindness cards, words written to us. Loved ones sending food to us. . . . And just encouragement from the community"*<br><br>- But public reactions also included to aggressive behaviour<br>→ Quote: *". . . She pulled down her mask and coughed at us"*<br>- these situations contributed to first responders not wanting to declare their occupations and place of work<br>→ Quote: *"If people would ask me what I did for work, I was kind of proud to say, you know, I work at the hospital.... [Now] I don't make a habit of telling anybody I work at the hospital, just because most people get kind of freaked out."*	California Southern University.<br>+ informed consent<br>+ the research and the manuscript did not receive funding<br>+ provision of measures to ensure validity of the data in the research (credibility, multiple participant perspectives, peer debriefing and review, reflexive journaling, and field notes)<br><br>- convenience sampling using the Facebook platform<br>- no information about conflict of interest<br>- possibility of non-transferable results to other first responders in the world<br>- researcher personal bias (e.g., mental health researcher)<br>- research participant bias

General	Study	Population	Exposure	Outcome	Results	Comments
					- First responders most often experienced stigma from the people closest to them – friends and family members<br>→ Quote: *"Usually people start to become very cognizant and aware exactly where you're standing so they don't touch those things. Yeah, I'm not allowed at my parent's house. [Friends] call me, but nobody wants me over [and] of course, nobody's coming over"*<br><br>- Friends created a physical separation between first responders and themselves<br>→ Quote: *stay away from me. When I did come home, . . . I'd be carrying on a conversation with [my mom], and if I took a step forward to like pick something up, she would take a step back, kind of like reflexively, like jumping back [and keep] this imaginary bubble. And in my house, I wasn't [even] allowed in the kitchen area."*<br><br>- Alienation frequently occurred because first responders found it difficult to find safe places to go and often found comfort in solitude<br>→ Quote: *"I don't enjoy people as much anymore; . . . like even my family will get together and I sit there but then I'm really annoyed with all of them....I don't want to engage. And I don't want to have conversations and I sometimes just want to go home and be like—where's the one place that nobody will be that I can go? I want to grab my wine and be alone. I don't want to talk to anybody. . . . On my ride home, which is like a 40-min ride, no radio, no, it was just silent."*<br><br>- Others were convinced or coerced into quarantine or separation from immediate family<br>→ Quote: *"Nobody wanted to be around you.... When you have [a] hard day or, you know, there's a lot going on, you have a lot of stress, and then you come home and then you're treated like you're, you know, a leper; . . . it doesn't make you feel very good; . . . there's nobody to share [anything] with."*<br><br>**Conditions increasing work-related stigmatization:**<br>- Distribution of misinformation (via social media)<br><br>**Measures to prevent/ deal with stigmatization at work:**	

General	Study	Population	Exposure	Outcome	Results	Comments
					- Participants described several solutions related to the stigma that was faced during the COVID-19 pandemic<br>- Participants mentioned how communication was an important component to consider<br>- Communication among colleagues<br>→ Quote: "*Talk to your colleagues or talk to somebody that you trust about what you're going through mentally, emotionally"*<br>- Communication among those in supervisor or managerial roles<br>→ Quote: *"I think there needs to be a lot more communication and honesty"*<br>- Participants also described the importance of health care workers and first responders maintaining a connection with those outside their professional roles, including other members of the general population and those in their communities<br>→ Quote: *"Just keep in touch with these people, like they're not diseased, . . . continue to call them and keep talking with them; . . . people should make an effort to continue to speak to these people"*<br>- Many participants described improvements related to education and dissemination of science-based information related to the COVID-19 pandemic as an important potential solution<br>→ Quote1: *"More real education and less completely false news would be great"*<br>→ Quote 2: *"I think education could definitely help next time"*<br>- participants stated that *"more facts"* would be helpful<br>→ Quote 1: "*The biggest thing is misinformation"*<br>→ Quote 2: *"Public awareness campaigns and things like that could do a lot of good"*<br>→ Quote 3: *"Public health education, educating the public on the modes of transmission of some conditions like this one"*<br>→ Quote 4: *"I don't think there really is anything you can do aside from educating the public"*<br><br>- Even when discussing potential solutions, participants still acknowledged the challenges and difficulties that came to stigma faced by health care workers and first responders<br>→ Quote: *"How do you simultaneously convey the message that this is something that needs to be taken seriously as well as then downplay the effect of people who are most exposed?"*	

General	Study	Population	Exposure	Outcome	Results	Comments
					- the messages conveyed how difficult it would be tackling stigma in first responders<br>*→ Quote: "You're always gonna have people that are gonna be rude; . . . there's always gonna be the people that think that you're icky and infected, and I don't think we'll ever get away from that"*<br><br>**Unfavourable health consequences associated with stigmatization:** Negative feelings, stress, alcohol usage. Results are shown in Tab. 2.<br><br>**Stigmatization studied in association with return-to-work:** *yes* was not investigated	

n = sample size, n.a. = not applicable, n.r. = not reported, SD = standard deviation
