# Supplementary material for: Stigmatization from Work-Related COVID-19 Exposure: A Systematic Review with Meta-Analysis
Source: Int J Environ Res Public Health. 2021 Jun 8;18(12):6183. doi: 10.3390/ijerph18126183 (PMC8226580; doi:10.3390/ijerph18126183)
Supplement: Supplementary file 1 [file ijerph-18-06183-s001.zip › Supplementary_ijerph-1235285/Table_S6.pdf]

<table>
<tr><th>General</th><th>Exposure</th><th>Outcome</th><th>Results</th></tr>
<tr><td>#<br>First Author, Year, Country</td><td>Describe how exposure was measured<br><br>Form of stigmatization:</td><td>Describe how outcome was measured</td><td>Prevalence:<br><br>Association with stigmatization:</td></tr>
<tr><td>7288<br>Chatterjee<br>2020, India</td><td>One question: Have you been ostracized?<br><br>Form of stigmatization:<br><u>Self-stigma (perceived): social exclusion</u><br>“ostracized by friends, neighbor, and the society for working in the hospital for being exposed and spreading<br>others“</td><td>Depression, Anxiety, and Stress Scale (DASS-21): depression, anxiety, stress (4-point Likert scale, 0 = “didn’t apply to me at all”, 3 = “much or mostly applied to me”) in the past 1 week</td><td>Prevalence:<br>Tab.: Prevalence of depression, anxiety and stress
<table>
<tr><th></th><th>Yes<br>n (%)</th><th>No<br>n (%)</th></tr>
<tr><td>Depression</td><td>53 (34.9)</td><td>99 (65.1)</td></tr>
<tr><td>Anxiety</td><td>60 (39.5)</td><td>92 (60.5)</td></tr>
<tr><td>Stress</td><td>50 (32.9)</td><td>102 (67.1)</td></tr>
</table>
<br>Association with stigmatization:<br>Tab1-3: Association between mental state and stigma experience
<table>
<tr><th colspan="2"></th><th colspan="2">Have you been ostracized?</th><th>$X^{2/t}$</th><th>p</th></tr>
<tr><th colspan="2"></th><th>Yes<br>n (%)</th><th>No<br>n (%)</th><th></th><th></th></tr>
<tr><td rowspan="2">Depression</td><td>Yes</td><td>20 (37.7)</td><td>33 (62.3)</td><td rowspan="2">16.6</td><td rowspan="2">0.002</td></tr>
<tr><td>No</td><td>12 (12.1)</td><td>87 (87.9)</td></tr>
<tr><td rowspan="2">Anxiety</td><td>Yes</td><td>23 (38.3)</td><td>37 (61.7)</td><td rowspan="2">25.6</td><td rowspan="2">0.001</td></tr>
<tr><td>No</td><td>9 (9.8)</td><td>83 (90.2)</td></tr>
<tr><td rowspan="2">Stress</td><td>Yes</td><td>21 (42.0)</td><td>29 (58.0)</td><td rowspan="2">23.2</td><td rowspan="2">0.001</td></tr>
<tr><td>No</td><td>11 (10.8)</td><td>91 (89.2)</td></tr>
</table></td></tr>
<tr><td>2030<br>Chew ,<br>2020,<br>Singapore</td><td>Healthcare Workers Stigma Scale (HWSS), 12 items, 4-point Likert scale (1 =strongly disagree, 4= strongly agree)<br><br>4 subscales:<br>1.Self-stigma (Personalized stigma)<br>2.Disclosure concerns<br>3.Concerns about public attitudes<br>4. Negative self-image<br><br>Form of stigmatization:</td><td>Perceived Stress: Perceived Stress Scale (PSS), 10 items, 5-point Likert scale (0=never, 4=very often)<br><br>Traumatic Stress: Impact of Event Scale-Revised (IES-R); 22 items, 5-point Likert scale (0=never, 4=very often)</td><td>Prevalence:<br>Tab. Prevalence of PSS and IES-R at baseline and follow-up
<table>
<tr><th></th><th>Baseline<br>(mean SD)</th><th>Follow-up<br>(mean SD)</th><th>t-test (t)</th></tr>
<tr><td>PSS</td><td>28.1 (6.63)</td><td>27.0 (5.87)</td><td>2.084</td></tr>
<tr><td>IES-R total score</td><td>15.0 (14.6)</td><td>14.5 (14.4)</td><td>0.404</td></tr>
<tr><td>IES-R Intrusion subscale</td><td>0.72 (0.74)</td><td>0.67 (0.66)</td><td>0.765</td></tr>
<tr><td>IES-R Avoidance subscale</td><td>0.70 (0.70)</td><td>0.70 (0.70)</td><td>-0.133</td></tr>
<tr><td>IES-R Hyperarousal subscale</td><td>0.61 (0.70)</td><td>0.57 (0.73)</td><td>0.539</td></tr>
</table>
<br>Association with stigmatization:<br><br>Tab. Risk factors for mental health outcomes amongst residents in training from national healthcare group residencies at the 2nd time point (linear regression)</td></tr>
</table>

<table>
<tr><th>General</th><th>Exposure</th><th>Outcome</th><th>Results</th></tr>
<tr>
<td></td>
<td><u>Self-stigma (internalized):</u> negative self-image (feeling inferior to others due to occupation)<br><u>Self-stigma (anticipated):</u> own perceived consequences, fear of rejection, disclosure concerns (control the information or keeping occupation a secret), concerns about public attitudes (what most people think of HCWs)</td>
<td></td>
<td>
<table>
<tr><td>Variable</td><td>B</td><td>ß</td><td>95% CI</td></tr>
<tr><td>PSS</td><td>0.134</td><td>0.157</td><td>0.024–0.244</td></tr>
<tr><td>IES-R, PTS symptoms</td><td>0.477</td><td>0.228</td><td>0.241–0.714</td></tr>
</table>
</td>
</tr>
<tr>
<td>883<br>Do Duy,<br>2020,<br>Vietnam</td>
<td>Stigma scale: self-developed instrument: 12 questions (4-point Likert scale), sum up score 0-36<br><br>Items from group discussion with HCW and investigators, adaption of Berger’s HIV Stigma Scale for terms and phrases (validity checked with COSMIN)<br><br>Form of stigmatization:<br><u>Self-stigma (anticipated):</u> Feel unsafe to be a health worker, Feel blamed by relatives or friends, Try to hide being quarantined, Try to avoid going out<br><br><u>Self-stigma (internalized):</u> Feel guilty because of being isolated, Feel ashamed of being isolated<br><br><u>Self-stigma (perceived): discrimination</u> People talk</td>
<td>DASS-21: depression, anxiety, stress (4-point Likert scale, 0 = never, 3 = almost always)<br><br>Depression (≥ 4): mild: 5-6; moderate: 7-10; severe: 11-13; extremely severe: ≥ 14<br><br>Anxiety (≥ 3): mild: 4-5; moderate: 6-7; severe: 8-9; extremely severe: ≥ 10<br><br>Stress (≥ 4): mild: 8-9; moderate: 10-12; severe: 13-16; extremely severe: ≥ 17</td>
<td>
Prevalence:<br><br>
Table: S1: Descriptive statistics
<table>
<tr><td>DASS-21</td><td>Male (n= 11)<br>Median (IQR)</td><td>Female (n= 60)<br>Median (IQR)</td></tr>
<tr><td>Depression</td><td>2 (0-3)</td><td>1 (0-3)</td></tr>
<tr><td>Anxiety</td><td>1 (0-2)</td><td>1 (0-2)</td></tr>
<tr><td>Stress</td><td>3 (1-5)</td><td>2 (1-5)</td></tr>
</table>
No significant difference between males and females<br><br>
Figure S1: Prevalence of depression, anxiety and stress (in %)
<table>
<tr><td>DASS-21</td><td>no</td><td>mild</td><td>moderate</td><td>severe</td><td>Extremely severe</td></tr>
<tr><td>Depression</td><td>86.9</td><td>8.2</td><td>3.2</td><td>1.6</td><td>0</td></tr>
<tr><td>Anxiety</td><td>85.3</td><td>8.2</td><td>3.7</td><td>1.6</td><td>1.2</td></tr>
<tr><td>Stress</td><td>95.1</td><td>1.6</td><td>1.6</td><td>1.6</td><td>0</td></tr>
</table>
<br>
Association with stigmatization:<br>
Correlation between stigmatization and DASS-21 depression, anxiety and stress<br><br>
Table 1: Spearman rank correlation r-value (95% CI)
<table>
<tr><td>DAS-21</td><td><u>Self-stigma</u><br>Negative self-image</td><td><u>Public stigma</u><br>Disclosure & personalized stigma</td><td><u>Public stigma</u><br>Concerns about public attitudes</td></tr>
<tr><td>Depression</td><td>0.35<br>(0.10-0.55)</td><td>0.32<br>(0.08-0.53)</td><td>0.33<br>(0.09-0.54)</td></tr>
</table>
</td>
</tr>
</table>

<table>
<tr><th>General</th><th>Exposure</th><th>Outcome</th><th>Results</th></tr>
<tr><td></td><td>behind back, People avoid touching and direct contact, People feel uncomfortable when around, Experienced discrimination actions</td><td></td><td>
<table>
<tr><td>Anxiety</td><td>0.45<br>(0.23-0.63)</td><td>0.36<br>(0.12-0.56)</td><td>0.39<br>(0.15-0.58)</td></tr>
<tr><td>Stress</td><td>0.32<br>(0.07-0.53)</td><td>0.16<br>(-0.01-0.40)</td><td>0.24<br>(-0.01-46)</td></tr>
</table>
</td></tr>
<tr><td>859 Elhadi 2020,<br>Libya</td><td>Stigmatization: single item “feeling stigmatized” (yes/no)<br><br>Form of stigmatization:<br><u>Self-stigma (perceived)</u></td><td>Depressive symptoms: Hospital Anxiety and Depression Scale (HADS, borderline abnormal: score 8-10, abnormal: score 11-21)<br><br>Anxiety symptoms: Hospital Anxiety and Depression Scale (HADS, borderline abnormal: score 8-10, abnormal: score 11-21)</td><td>
Prevalence:<br><br>
Table 2: Anxiety and depressive symptom levels grades among participants (in %)
<table>
<tr><th colspan="2">Outcome</th><th>n</th><th>%</th></tr>
<tr><td rowspan="2">Depressive symptoms</td><td>Borderline abnormal</td><td>236</td><td>31.7</td></tr>
<tr><td>Abnormal</td><td>420</td><td>56.4</td></tr>
<tr><td rowspan="2">Anxiety symptoms</td><td>Borderline abnormal</td><td>237</td><td>31.8</td></tr>
<tr><td>Abnormal</td><td>348</td><td>46.7</td></tr>
</table>
<br>Association with stigmatization:<br><br>
Table 3: Prevalence of depressive symptoms and association with feeling stigmatized
<table>
<tr><th>Variable</th><th>No depressive symptoms<br>n (%)</th><th>Depressive symptoms<br>n (%)</th><th>χ2 (Pearson's chi-square)</th><th>p-value</th></tr>
<tr><td>Feeling stigmatized</td><td></td><td></td><td></td><td></td></tr>
<tr><td>Yes</td><td>77 (23.7)</td><td>154 (36.7)</td><td rowspan="2">14.41</td><td rowspan="2">< 0.001</td></tr>
<tr><td>No</td><td>248 (76.3)</td><td>266 (63.3)</td></tr>
</table>
<br>Table 4: Prevalence of anxiety symptoms and association with feeling stigmatized
<table>
<tr><th>Variable</th><th>No depressive symptoms<br>n (%)</th><th>Depressive symptoms<br>n (%)</th><th>χ2 (Pearson's chi-square)</th><th>p-value</th></tr>
<tr><td>Feeling stigmatized</td><td></td><td></td><td></td><td></td></tr>
<tr><td>Yes</td><td>95 (23.9)</td><td>136 (39.1)</td><td rowspan="2">19.89</td><td rowspan="2">< 0.001</td></tr>
<tr><td>No</td><td>302 (76.1)</td><td>212 (60.9)</td></tr>
</table>
</td></tr>
<tr><td>7273<br>Elhadi, 2020,<br>Libya</td><td>Stigmatization: single item “feeling stigmatized” (yes/no)<br><br>Form of stigmatization:<br><u>Self-stigma (perceived)</u></td><td>Burnout: abbreviated Maslach Burnout Inventory (aMBI) (comprising three subscales: emotional exhaustion (EE), depersonalization (DP), and personal accomplishment (PA))</td><td>
Prevalence:<br>
Text and Supplementary Material
<table>
<tr><th>Variable</th><th>Total sample<br>n (%)</th><th>Men<br>n (%)</th><th>Women<br>n (%)</th></tr>
<tr><td>High emotional exhaustion</td><td>357 (67.1)</td><td>209 (71.1)</td><td>148 (62.2)</td></tr>
</table>
</td></tr>
</table>

<table>
<tr><th>General</th><th>Exposure</th><th>Outcome</th><th>Results</th></tr>
<tr><td></td><td></td><td></td><td>
<table>
<tr><td>High depersonalization</td><td>252 (47.4)</td><td>152 (51.7)</td><td>100 (42.0)</td></tr>
<tr><td>Decreased personal accomplishment</td><td>121 (22.7)</td><td>58 (19.7)</td><td>63 (26.5)</td></tr>
</table>
<br>
Association with stigmatization:<br>
Correlation analysis (Spearman’s rank-order correlation test)<br>
- significant positive correlation was identified between emotional exhaustion (rs (530)=0.174; p≤0.001) and feeling stigmatised<br>
- no statistically significant correlation between depersonalization and feeling stigmatized<br>
- no statistically significant correlation between personal accomplishment and feeling stigmatized
</td></tr>
<tr><td>7279 Greene 2020, UK</td><td>Being worried about rejection or stigmatization for being an NHS worker: single Likert scale ranging from 0 (“not at all”) to 4 (“extremely”)<br><br>Form of stigmatization:<br><u>Self-stigma (perceived)</u></td><td>Distress: Having clinically significant PTDS, depression, and/or anxiety<br><br>PTDS: PTSD subscale of the International Trauma Questionnaire (ITQ)<br><br>Depression: Patient Health Questionnaire-9 (PHQ-9)<br><br>Anxiety: Generalized Anxiety Disorder Scale (GAD-7)</td><td>
Prevalence:<br>
Tab: Prevalence of distress, PTSD, depression and anxiety
<table>
<tr><th></th><th>n</th><th>%</th></tr>
<tr><td>Distress</td><td>572</td><td>57.9</td></tr>
<tr><td>PTDS</td><td>246</td><td>22.5</td></tr>
<tr><td>Depression</td><td>477</td><td>46.9</td></tr>
<tr><td>Anxiety</td><td>470</td><td>47.3</td></tr>
</table>
<br>
Association with stigmatization:<br><br>
Table 3: Distress
<table>
<tr><th></th><th>Total sample OR (95% CI)</th><th>p-value</th></tr>
<tr><td>Perceived stigma</td><td></td><td></td></tr>
<tr><td>no</td><td>1.0 (Reference)</td><td></td></tr>
<tr><td>yes</td><td>1.25 (1.01-1.43)</td><td>< 0.0005</td></tr>
</table>
<br>
Table 3: PTDS
<table>
<tr><th></th><th>Total sample OR (95% CI)</th><th>p-value</th></tr>
<tr><td>Perceived stigma</td><td></td><td></td></tr>
<tr><td>no</td><td>1.0 (Reference)</td><td></td></tr>
<tr><td>yes</td><td>1.37 (1.20-1.58)</td><td>< 0.0005</td></tr>
</table>
<br>
Table 3: Depression
<table>
<tr><th></th><th>Total sample OR (95% CI)</th><th>p-value</th></tr>
</table>
</td></tr>
</table>

<table>
<tr><th>General</th><th>Exposure</th><th>Outcome</th><th>Results</th></tr>
<tr><td></td><td></td><td></td><td>
<table>
<tr><td>Perceived stigma</td><td></td><td></td></tr>
<tr><td>no</td><td>1.0 (Reference)</td><td></td></tr>
<tr><td>yes</td><td>1.30 (1.15-1.48)</td><td>< 0.0005</td></tr>
</table>
<br>
Table 3: Anxiety
<table>
<tr><th></th><th>Total sample<br>OR (95% CI)</th><th>p-value</th></tr>
<tr><td>Perceived stigma</td><td></td><td></td></tr>
<tr><td>no</td><td>1.0 (Reference)</td><td></td></tr>
<tr><td>yes</td><td>1.23 (1.09-1.40)</td><td>< 0.005</td></tr>
</table>
<br>
*all adjusted for predictors: age, gender, ethnicity, annual household income, relationship, caring for children at home, work-related and COVID-19-related predictors
</td></tr>
<tr><td>759 Juan<br>2020,<br>China</td><td>Stigmatization and rejection in neighbourhood because of hospital work: 1 item question (yes, no)<br><br>Form of stigmatization:<br><u>Self-stigma (perceived): discrimination</u></td><td>Psychological reactions: Impact of Event Scale-Revised (IES-R, normal: score 0−23, mild: score 24–32, moderate: score 33–36, severe: score >37)<br><br>Anxiety disorder: Generalized Anxiety Disorder 7 (GAD-7, normal: score 0–4, mild: score 5–9, moderate: score 10–14, severe: score 15–21)<br><br>Depression: Patient Health Questionnaire 9 (PHQ-9,normal: score 0–4, mild: score 5–9, moderate: score 10–14, severe: score 15–21)<br><br>Obsessive-compulsive symptoms: Yale-Brown Obsessive-Compulsive Scale (Y-BOCS, normal: score 0–5, mild: score 6–15, moderate: score 16–25, severe: score 26–40)<br><br>Somatization symptoms: Patient Health Questionnaire 15 (PHQ-15, normal: score 0–4, mild: score 5–9, moderate: score 10–14, severe: score 15–30)</td><td>
Prevalence:<br><br>
Figure 1 and text: Prevalence of psychological distress, obsessive-compulsive symptoms, somatization symptoms, anxiety disorder, or depression among all participants (in %)
<table>
<tr><th>Outcome</th><th>%</th></tr>
<tr><td>Psychological distress (obsessive-compulsive symptoms, somatization symptoms, anxiety disorder, or depression)</td><td>43.2</td></tr>
<tr><td>Obsessive-compulsive symptoms</td><td>37.5</td></tr>
<tr><td>Somatization symptoms</td><td>33.3</td></tr>
<tr><td>Anxiety disorder</td><td>31.6</td></tr>
<tr><td>Depression</td><td>29.6</td></tr>
</table>
n not reported<br><br>
Association with stigmatization:<br><br>
Table 4: Multiple logistic regression analysis for the factor “stigmatization and rejection in neighbourhood because of hospital work?” associated with psychological distress related to the COVID-19 crisis (in AOR (95% CI))
<table>
<tr><th>Outcome</th><th>AOR (95% CI)</th></tr>
<tr><td>Psychological reaction</td><td>1.705 (0.827–3.516)</td></tr>
<tr><td>Anxiety symptoms</td><td>1.329 (0.652–2.709)</td></tr>
<tr><td>Depression symptoms</td><td>2.297 (1.138–4.637)</td></tr>
<tr><td>Obsessive-compulsive symptoms</td><td>1.842 (0.912–3.72)</td></tr>
<tr><td>Somatization symptoms</td><td>1.418(0.697–2.881)</td></tr>
</table>
<br>
*use of AOR, but not details on adjustment
</td></tr>
</table>

<table>
<tr><th>General</th><th>Exposure</th><th>Outcome</th><th>Results</th></tr>
<tr>
<td>749 Khanal 2020, Nepal</td>
<td>1 item: Stigma faced due to COVID-19 (yes, no, don’t want to answer)<br><br>Form of stigmatization:<br><u>Self-stigma (perceived): discrimination, aggressive behaviour/mobbing</u></td>
<td>Anxiety and Depression: Hospital Anxiety and Depression Scale (HADS), 14-items, total scores: normal: 0–7, borderline abnormal: 8–10 and abnormal: 11–21<br><br>Insomia: Insomia Severity Index (ISI), 7 items; no clinically significant insomnia (0–7), subthreshold insomnia (8–14), moderate clinical insomnia (15–21) and severe clinical insomnia (22–28)</td>
<td>Prevalence:<br>Tab. Prevalence of anxiety, depression and insomnia
<table>
<tr><td>Mental Health Condition</td><td>Categories</td><td>Total n (%)</td></tr>
<tr><td>Anxiety</td><td>Normal</td><td>276 (58.1%)</td></tr>
<tr><td></td><td>Borderline</td><td>112 (23.6)</td></tr>
<tr><td></td><td>Abnormal</td><td>87 (18.3)</td></tr>
<tr><td>Depression</td><td>Normal</td><td>297 (62.5)</td></tr>
<tr><td></td><td>Borderline</td><td>114 (24.0)</td></tr>
<tr><td></td><td>Abnormal</td><td>64 (13.5)</td></tr>
<tr><td>Insomnia</td><td>No clinical significant</td><td>314 (66.1)</td></tr>
<tr><td></td><td>Sub threshold</td><td>127 (26.7)</td></tr>
<tr><td></td><td>Moderate</td><td>27 (5.7)</td></tr>
<tr><td></td><td>Severe</td><td>7 (1.5)</td></tr>
</table>
-> Nurses have significantly higher scores (borderline, abnormal) in anxiety, depression and insomnia as compared to doctors and other health workers<br><br>
Association with Stigmatization:<br><br>
Tab.: Factors associated with anxiety, depression and insomnia (multivariate logistic regression)
<table>
<tr><td rowspan="2"></td><td colspan="2">Faced Stigma</td><td rowspan="2">Unadjusted OR (95%CI)</td><td rowspan="2">Adjusted OR (95%)</td></tr>
<tr><td>No<br>n (%)</td><td>Yes<br>n (%)</td></tr>
<tr><td>Anxiety</td><td>68 (34.2)</td><td>131 (65.8</td><td>2.36 (1.62-3.44)</td><td>2.47(1.62-3.76)</td></tr>
<tr><td>Depression</td><td>57 (34.8)</td><td>116 (65.2)</td><td>2.13 (1.45–3.12)</td><td>2.05 (1.34–3.11)</td></tr>
<tr><td>Insomnia</td><td>37 (31.6)</td><td>80 (68.4)</td><td>2.26 (1.45–3.52)</td><td>2.37 (1.46–3.84)</td></tr>
</table>
*adjusted for gender, ethnicity, profession, education, living with elderly, family member with chronic disease, precautionary measures in the workplace, faced stigma, worked overtime, awareness about government incentive and history of medication for mental health problem</td>
</tr>
<tr>
<td>948 Monterrosa-Castro, 2020, Colombia</td>
<td>Social discrimination for working as a general practitioner (questions not described)<br><br>Forms of stigmatization:<br><u>Self-Stigma (perceived): discrimination</u></td>
<td>Generalized Anxiety Disorder (GAD): Generalized Anxiety Disorder Scale (GAD-7), 7 Items, total score of 0–21, ≥ 10 indicates GAD symptoms</td>
<td>Prevalence:<br>Tab. Prevalence of GAD
<table>
<tr><td>Variable</td><td>Total (n/%)</td><td>With symptoms of generalized anxiety disorder, n=209 (39.3%)</td><td>Without symptoms of generalized anxiety disorder, n=322 (60.6%)</td></tr>
<tr><td>Social discrimination for working as a general practitioner</td><td>207 (38.9)</td><td>106 (50.7)</td><td>101 (31.3))</td></tr>
</table></td>
</tr>
</table>

<table>
<tr><th>General</th><th>Exposure</th><th>Outcome</th><th>Results</th></tr>
<tr><td></td><td></td><td></td><td>
<table>
<tr><td></td><td></td><td>($chi^2$) p</td><td><0.001</td></tr>
</table>
<br>
Association with stigmatization:<br><br>
Tab.: Psychosocial and demographic factors associated with symptoms of generalized anxiety disorder, not adjusted logistic regression (N=531)
<table>
<tr><td>Variables</td><td>crude OR (95% CI)</td></tr>
<tr><td>Social discrimination for working as a general practitioner</td><td>2.25 (1.57 to 3.22)</td></tr>
</table>
</td></tr>
<tr><td>7261<br>Ramaci,<br>Italy, 2020</td><td>“Stigma discrimination”<br>“Stigma fear”: no description of definition included; adapted from See et al. 2011 (answer options: 0 = strongly disagree, 1 = disagree, 2 = agree, 3 = strongly agree)<br><br>Form of stigmatization:<br><u>Self-stigma (perceived): discrimination</u><br><br><u>Self-stigma (anticipated)</u></td><td>Professional Quality of Life Scale (ProQOL): 3 dimensions: compassion fatigue (7 items), compassion satisfaction (8 items), risk of burnout (7 items); 5-point Likert scale</td><td>
Prevalence:<br>
Tab: Prevalence of compassion fatigue, satisfaction and risk of burnout with regard to sex
<table>
<tr><td>Sex</td><td>Compassion fatigue<br>Mean (SD)</td><td>Compassion satisfaction<br>Mean (SD)</td><td>Risk of burnout<br>Mean (SD)</td></tr>
<tr><td>Males</td><td>2.12 (1.3)</td><td>3.81 (1.0)</td><td>1.62 (1.0)</td></tr>
<tr><td>Females</td><td>2.52 (SD 1.2)</td><td>3.56 (0.97)</td><td>2.02 (0.99)</td></tr>
</table>
<br>
Tab.: Association with stigmatization (zero-order correlation):
<table>
<tr><td>Stigma</td><td>Compassion fatigue<br>Mean (SD)</td><td>Compassion satisfaction<br>Mean (SD)</td><td>Risk of burnout<br>Mean (SD)</td></tr>
<tr><td>Perceived stigma</td><td>0.307</td><td>-0.263</td><td>0.348</td></tr>
<tr><td>Anticipated stigma</td><td>0.341</td><td>-0.173</td><td>0.226</td></tr>
</table>
<br>
Multiple regression
<table>
<tr><td>Stigma</td><td colspan="2">Perceived stigma (discrimination)</td><td colspan="2">Anticipated stigma (Stigma fear)</td></tr>
<tr><td></td><td>Beta</td><td>t</td><td>Beta</td><td>T</td></tr>
<tr><td>Compassion fatigue</td><td>0.348</td><td>4.50</td><td>0.291</td><td>5.41</td></tr>
<tr><td>Compassion satisfaction</td><td>-0.231</td><td>-3.73</td><td>-0.141</td><td>-2.54</td></tr>
<tr><td>Risk of burnout</td><td>0.317</td><td>5.54</td><td>0.171</td><td>3.08</td></tr>
</table>
</td></tr>
<tr><td>832 Sharma 2020,<br>USA</td><td>Stigma from community: instrument not reported<br><br>Form of stigmatization:<br><u>Self-stigma (perceived)</u></td><td>Emotional distress/burnout (dichotomous variable): instrument not reported</td><td>
Prevalence:<br><br>
Figure 2 and Table 2: Emotional distress/Burnout
<table>
<tr><th>Health care professional</th><th>%</th></tr>
<tr><td>Total sample</td><td>58</td></tr>
<tr><td>Doctor</td><td>49</td></tr>
</table>
</td></tr>
</table>

<table>
<tr><th>General</th><th>Exposure</th><th>Outcome</th><th>Results</th></tr>
<tr><td></td><td></td><td></td><td>
<table>
<tr><td>Doctor in training</td><td>48</td></tr>
<tr><td>Advanced practice provider</td><td>56</td></tr>
<tr><td>Respiratory therapist</td><td>55</td></tr>
<tr><td>Nurse</td><td>64</td></tr>
</table>
n not reported<br><br>
Association with stigmatization:<br><br>
Table 3
<table>
<tr><td>Outcome</td><td>RR (95% CI)</td></tr>
<tr><td>Emotional distress/burnout</td><td>1.32 (1.24–1.41)</td></tr>
</table>
Adjusted for: n.r.</td></tr>
<tr><td>851<br>Taylor,<br>2020<br>Canada</td><td>Attitudes from the general population towards HCW<br><br>HCW Stigmatization Survey: self-developed, 8 items, 5-point scale (0=strongly disagree, 4=strongly agree), excellent reliability<br><br><br>Forms of stigmatization:<br><u>Public Stigma: stereotypes, discrimination, social exclusion</u></td><td>Covid Stress Syndrome (CSS): 5 items 1. Fears about COVID-19 dangerousness and contamination, 2. Fears about socio-economic effects of COVID-19, 3. Fears that foreigners are spreading COVID-19, 4. COVID-related traumatic stress symptoms, 5. COVID-related compulsive checking and reassurance-seeking</td><td>Prevalence:<br>not reported<br><br>
Association with stigmatization:<br>
Tab.: Correlation between CSS and HCW stigmatization survey
<table>
<tr><td>Covid Stress Scale (CSS</td><td>R</td></tr>
<tr><td>Fears about COVID-19 dangerousness and contamination</td><td>0.45</td></tr>
<tr><td>Fears about socio-economic effects of COVID-19</td><td>0.41</td></tr>
<tr><td>Fears that foreigners are spreading COVID-19</td><td>0.32</td></tr>
<tr><td>COVID-related traumatic stress symptoms</td><td>0.30</td></tr>
<tr><td>COVID-related compulsive checking and reassurance-seeking</td><td>0.24</td></tr>
</table>
<br>
Tab: Factor loadings for measures of stigma, avoidance, and COVID Stress Syndrome.
<table>
<tr><td></td><td>Factor 1</td><td>Factor 2</td></tr>
<tr><td>CSS: Fears about COVID-19 dangerousness and contamination</td><td>.81</td><td>.07</td></tr>
<tr><td>CSS: Fears about socio-economic effects of COVID-19</td><td>.80</td><td>-.04</td></tr>
<tr><td>CSS: Fears that foreigners are spreading COVID-19</td><td>.69</td><td>-.06</td></tr>
<tr><td>CSS: COVID-related traumatic stress symptoms</td><td>.68</td><td>.03</td></tr>
<tr><td>CSS: COVID-related compulsive checking and reassurance-seeking</td><td>.69</td><td>-.13</td></tr>
<tr><td>Avoidance of supermarkets and drug stores</td><td>.43</td><td>.13</td></tr>
<tr><td>Healthcare Worker Stigmatization Survey</td><td>.42</td><td>.19</td></tr>
<tr><td>Preference for staying home</td><td>-.03</td><td>.90</td></tr>
<tr><td>Preference for avoiding people</td><td>.08</td><td>.76</td></tr>
</table>
*maximum likelihood factor analysis of the COVID Stress Scales, avoidance measures, and HCW Stigmatization Survey</td></tr>
</table>

<table>
<tr><th>General</th><th>Exposure</th><th>Outcome</th><th>Results</th></tr>
<tr><td>865 Uvais, India, 2020</td><td>Perceived stigma: stigma scale with 13 items (each scored on a 5-point Likert scale, total score 0 - 52 total score ≥26: high levels of perceived stigma)<br><br>Form of stigmatization:<br><u>Self-stigma (perceived): discrimination, social exclusion</u><br><br><u>Self-stigma (anticipated)</u></td><td>Perceived Stress Scale (PSS-10): 10 items, 5-point Likert scale, total score 0– 40</td><td>Prevalence:<br>Mean: 20.6 (SD 6.76)<br><br>Association with stigmatization:<br>Correlation stigma and Stress: Pearson correlation coefficient r = 0.604</td></tr>
<tr><td>7220 Zandifar 2020, Iran</td><td>Stigma: 22-item modified stigma-related questionnaire adopted from the HIV Stigma Scale (5-point Likert scale: strongly disagree to strongly agree)<br><br>Form of stigmatization:<br><u>Self-stigma (perceived): discrimination</u></td><td>PTDS: Posttraumatic Stress Disorder 8-Item Questionnaire (PTSD-8)</td><td>Prevalence:<br><br>Table 1 and Table 2: Prevalence of intrusion, avoidance, and hypervigilance (in %)
<table>
<tr><th></th><th>Male</th><th>Female</th><th>Total sample</th></tr>
<tr><td>Intrusion</td><td>37.4</td><td>46.9</td><td>42.2</td></tr>
<tr><td>Avoidance</td><td>23.5</td><td>35.3</td><td>31.4</td></tr>
<tr><td>Hypervigilance</td><td>33.1</td><td>39.7</td><td>37.2</td></tr>
</table>
- Significant difference between males and females for intrusion and avoidance, but not for hypervigilance<br><br>Association with stigmatization:<br>Table 4: Correlation between PTSD and stigma (with Spearman correlation)
<table>
<tr><th></th><th>PTDS total score</th><th>Intrusion score</th><th>Avoidance score</th><th>Hypervigilance score</th></tr>
<tr><td>Stigma score</td><td>0.836*</td><td>0.724*</td><td>0.693*</td><td>0.767*</td></tr>
</table>
* Statistically significant</td></tr>
<tr><td>905 Zhu 2020, China</td><td>Feeling that family and friends avoid contact because of work: 1 item question (answer options: agree, disagree, unsure)<br><br>Form of stigmatization:<br><u>Self-stigma (perceived): social exclusion</u></td><td>Depression: Patient Health Questionnaire 9 (PHQ-9, major depression: score ≥10)<br><br>Anxiety: Generalized Anxiety Disorder 7 (GAD-7, anxiety disorder: score ≥8)<br><br>Psychological stress: Impact of Event Scale-Revised (IES-R, stress: score: >33)</td><td>Prevalence:<br><br>Table 1 and Figure 2
<table>
<tr><th>Outcome</th><th>n</th><th>%</th></tr>
<tr><td>No symptoms</td><td>n.r.</td><td>63.4</td></tr>
<tr><td>Acute stress</td><td>1509</td><td>29.8</td></tr>
<tr><td>Depression</td><td>381</td><td>13.5</td></tr>
<tr><td>Anxiety</td><td>1218</td><td>24.1</td></tr>
</table></td></tr>
</table>

| General | Exposure | Outcome | Results |
|---|---|---|---|

| | | |
|---|---|---|
| Acute stress and depression | n.r. | 1.5 |
| Acute stress and anxiety | n.r. | 7.5 |
| Depression and anxiety | n.r. | 0.9 |
| Acute stress and depression and anxiety | n.r. | 10.4 |

n not reported

**Association with stigmatization:**
**Table 2: Depression**

| | Depression (n = 681) | No depression (n = 4,381) | p |
|---|---|---|---|
| Do you feel that family members and friends have avoided contact with you because of your work? | | | |
| Disagree | 215 (31.6%) | 2298 (52.5%) | <0.001 |
| Agree | 240 (35.2%) | 747 (17.1%) | |
| Unsure | 226 (33.2%) | 1336 (30.5%) | |

**Table 2: Anxiety**

| | Anxiety (n = 1,218) | No anxiety (n = 3,844) | p |
|---|---|---|---|
| Do you feel that family members and friends have avoided contact with you because of your work? | | | |
| Disagree | 414 (34.0%) | 2099 (54.6%) | <0.001 |
| Agree | 389 (31.9%) | 598 (15.6%) | |
| Unsure | 415 (34.1%) | 1147 (29.8%) | |

**Table 2: Psychological stress**

| | Psychological stress (n = 1,509) | No psychological stress (n = 3,553) | p |
|---|---|---|---|
| Do you feel that family members and friends have avoided contact with you because of your work? | | | |
| Disagree | 510 (33.8%) | 2003 (56.4%) | <0.001 |
| Agree | 463 (30.7%) | 524 (14.8%) | |
| Unsure | 536 (35.5%) | 1026 (28.9%) | |

**Table 3 and Supplementary Material (ST1 and ST2): Depression**

| | Total sample OR (95% CI) | Men OR (95% CI) | Women OR (95% CI) |
|---|---|---|---|

<table>
<tr><th>General</th><th>Exposure</th><th>Outcome</th><th>Results</th></tr>
<tr><td></td><td></td><td></td><td>
<table>
<tr><td>Feeling that family and friends avoid contact because of work</td><td></td><td></td><td></td></tr>
<tr><td>Don´t avoid</td><td>1.0 (Reference)</td><td>1.0 (Reference)</td><td>1.0 (Reference)</td></tr>
<tr><td>Avoid</td><td>1.94 (1.55-2.42)</td><td>n.r.</td><td>1.99 (1.57-2.52)</td></tr>
<tr><td>Unsure</td><td>1.21 (0.97-1.50)</td><td>n.r.</td><td>1.20 (0.95-1.51)</td></tr>
</table>
<br>
Table 3 and Supplementary Material (ST1 and ST2): Anxiety
<table>
<tr><td></td><td>Total sample<br>OR (95% CI)</td><td>Men<br>OR (95% CI)</td><td>Women<br>OR (95% CI)</td></tr>
<tr><td>Feeling that family and friends avoid contact because of work</td><td></td><td></td><td></td></tr>
<tr><td>Don´t avoid</td><td>1.0 (Reference)</td><td>1.0 (Reference)</td><td>1.0 (Reference)</td></tr>
<tr><td>Avoid</td><td>1.83 (1.52-2.21)</td><td>n.r.</td><td>2.03 (1.42-2.90)</td></tr>
<tr><td>Unsure</td><td>1.22 (1.03-1.45)</td><td>n.r.</td><td>1.19 (0.85-1.67)</td></tr>
</table>
<br>
Table 3 and Supplementary Material (ST1 and ST2): Psychological stress
<table>
<tr><td></td><td>Total sample<br>OR (95% CI)</td><td>Men<br>OR (95% CI)</td><td>Women<br>OR (95% CI)</td></tr>
<tr><td>Feeling that family and friends avoid contact because of work</td><td></td><td></td><td></td></tr>
<tr><td>Don´t avoid</td><td>1.0 (Reference)</td><td>1.0 (Reference)</td><td>1.0 (Reference)</td></tr>
<tr><td>Avoid</td><td>2.01 (1.69-2.40)</td><td>n.r.</td><td>1.45 (1.23-1.71)</td></tr>
<tr><td>Unsure</td><td>1.44 (1.23-1.69)</td><td>n.r.</td><td>0.70 (0.54-0.91)</td></tr>
</table>
<br>
*all adjusted for: age, occupation, years of working, past medical history, parent status, work-related and COVID-19-related predictors
</td></tr>
<tr><td>5386<br>Zolnikov<br>2020,<br>worldwide</td><td>Stigma<br><br>Form of stigmatization:<br><u>Self-stigma (perceived); discrimination, social exclusion, aggressive behaviour/mobbing</u><br><br><u>Self-Stigma (internalized)</u></td><td>Negative feelings, stress, alcohol usage</td><td>Prevalence:<br>n.a.<br>Association with stigmatization:<br>- common words to describe emotions and feelings as a result of being stigmatized, included “sadness”, feeling “blue” and “extremely stress[ed]” while living in a situation that “is so demoralizing”<br>- reactions to these feelings included alcohol use<br>→ quote: “I feel very isolated, lonely, depressed even. I found my alcohol intake increased”</td></tr>
</table>

n = sample size, n.a. = not applicable, n.r. = not reported, OR = odds ratio, RR = relative risk
